# Systematic identification and expression analysis of *bHLH* gene family reveal their relevance to abiotic stress response and anthocyanin biosynthesis in sweetpotato

**DOI:** 10.1186/s12870-024-04788-0

**Published:** 2024-03-01

**Authors:** Fen Guo, Xiaoqing Meng, Haiting Hong, Siyuan Liu, Jing Yu, Can Huang, Tingting Dong, Huixue Geng, Zongyun Li, Mingku Zhu

**Affiliations:** Institute of Integrative Plant Biology, School of Life Sciences, Jiangsu Normal University, 101 Shanghai Road, Xuzhou, Jiangsu Province 221116 China

**Keywords:** Abiotic stress, Anthocyanin biosynthesis, bHLH transcription factor, Expression profile, Sweetpotato

## Abstract

**Background:**

bHLH transcription factors play significant roles in regulating plant growth and development, stress response, and anthocyanin biosynthesis. Sweetpotato is a pivotal food and industry crop, but little information is available on sweetpotato *bHLH* genes.

**Results:**

Herein, 227 putative *IbbHLH* genes were defined on sweetpotato chromosomes, and fragment duplications were identified as the dominant driving force for *IbbHLH* expansion. These IbbHLHs were divided into 26 subfamilies through phylogenetic analysis, as supported by further analysis of exon-intron structure and conserved motif composition. The syntenic analysis between *IbbHLHs* and their orthologs from other plants depicted evolutionary relationships of *IbbHLHs*. Based on the transcriptome data under salt stress, the expression of 12 *IbbHLHs* was screened for validation by qRT-PCR, and differential and significant transcriptions under abiotic stress were detected. Moreover, IbbHLH123 and IbbHLH215, which were remarkably upregulated by stress treatments, had obvious transactivation activity in yeasts. Protein interaction detections and yeast two-hybrid assays suggested an intricate interaction correlation between IbbHLHs. Besides, transcriptome screening revealed that multiple *IbbHLHs* may be closely related to anthocyanin biosynthesis based on the phenotype (purple vs. white tissues), which was confirmed by subsequent qRT-PCR analysis.

**Conclusions:**

These results shed light on the promising functions of sweetpotato *IbbHLHs* in abiotic stress response and anthocyanin biosynthesis.

**Supplementary Information:**

The online version contains supplementary material available at 10.1186/s12870-024-04788-0.

## Background

Transcription factors (TFs) play a critical role in regulating the response of plants to adverse environmental conditions through recognizing and binding specific promoter elements to regulate the expression of associated genes [1, 2]. Presently, more than 60 TF families have been found in plants, such as NAC, WRKY, bHLH (basic helix-loop-helix), MYB, and bZIP TFs [3–5]. Among them, bHLH is the second-largest TF family [6], which generally contains a conserved bHLH domain consisting of about 60 aa, including two conserved motifs, one basic region and one HLH region [7, 8]. The N-terminal basic region is made up of about 10–15 aa, which acts as a DNA binding motif to help bHLH TFs combine with E-box or G-box sequences [7, 9]. The C-terminal HLH region contains two amphipathic α-helices separated by the variable-length loop, which is involved in forming homodimeric or heterodimeric complexes to alter the expression of downstream genes [9, 10].

To date, comprehensive documents have shown that bHLH TFs are crucial regulators in signal transduction networks, which modulate diverse developmental and metabolic processes of plants, including photomorphogenesis, flowering, and biosynthesis of secondary metabolites, and are of great significance for promoting plant tolerance or adaptation to adverse conditions [8, 11, 12]. Many studies have possessed that genetically modified plants overexpressing a *bHLH* gene displayed enhanced stress resistance. For instance, Arabidopsis AtbHLH112 TF was reported to regulate the transcription of stress-related genes to modulate physiological response to enhance salt and drought tolerance [13]. The transgenic expressions of *bHLH55* from maize, *MfbHLH38* from *Myrothamnus flabellifolia*, and *CsbHLH041* from cucumber, respectively, all significantly enhanced salt resistance of Arabidopsis [14–16]. And overexpression of *SlbHLH22* or *SlbHLH96* in tomato both confers increased drought tolerance by enhancing antioxidant capacity [17, 18]. These studies exhibit that bHLHs are potential candidates for crop genetic engineering under harsh environments.

Furthermore, bHLH TFs have also been found to participate the regulation of metabolic pathways including anthocyanin synthesis in many plants [19, 20]. The first bHLH TF isolated in maize was shown to regulate anthocyanin biosynthesis [21]. Other *bHLH* genes related to anthocyanin synthesis include *EGL3*, *GL3*, and *TT8* in Arabidopsis [22]; *PdTT8* in poplar [23]; and *MdbHLH3* in apple [24]. Besides, it has been found that bHLH TFs can interact with MYB and WD40 proteins to form the MYB–bHLH–WD40 (MBW) complex [25, 26]. For instance, FaEGL3 was found to interact with FaMYB5 and FaLWD1-like to regulate related promoters, thus committing to flavonoid accumulation in strawberry fruits [27].

Sweetpotato is one of the most widely cultivated crops worldwide and the only crop with starch storage roots in Convolvulaceae [28]. Sweetpotato has a wide range of applications, including human food, animal feed and industrial raw materials. In addition, it is of great significance in ensuring food safety in many developing countries due to its adaptability to constantly changing environments. However, sweetpotato production is still limited by various biotic and abiotic stresses [29]. The genome sequencing of sweetpotato has been completed [30], while information about the sweetpotato bHLH TFs is still scarce. Previously, 110 *IbbHLHs* were identified in the sweetpotato genome, and improved cold endurance was observed in *IbbHLH79*-overexpressing sweetpotato [31]. Similarly, transgenic tobacco plants overexpressing *IbbHLH33* displayed enhanced chilling resistance [32]. Moreover, the sweetpotato IbPYL8-IbbHLH66-IbbHLH118 complex was reported to mediate the ABA-dependent drought response [33]. And IbERF71 and IbMYB340-IbbHLH2 (JQ337863) could form the regulatory complex that coregulated anthocyanin accumulation via binding to the *IbANS1* promoter [34]. Herein, some discrepancies were detected when we intended to give complete overviews of *IbbHLH*s in sweetpotato, then 227 *IbbHLHs* were identified. And their molecular characteristics, phylogenetic relationships, gene structures, conserved domains, syntenic relationships and protein interactions were systematically studied. Furthermore, in order to screen new *IbbHLHs* related to stress response and anthocyanin biosynthesis, their expression patterns under different stress treatments and in various purple sweetpotato cultivars were also surveyed through transcriptome and qRT-PCR analysis. These data laid a foundation for further exploring the functions and regulatory mechanisms of IbbHLH TFs, and identifying promising members for stress tolerance and anthocyanin biosynthesis in sweetpotato.

## Results

### Identification and characterization of *IbbHLHs* in sweetpotato genomes

Previously, only 110 candidate *IbbHLH* genes were isolated in sweetpotato genomes using the HMM profile of bHLH [31]. In this study, some discrepancies were detected in the comprehensive overview of *IbbHLHs* in the sweetpotato genome based on the HMM profile and all bHLH members in Arabidopsis and rice, and a total of 227 putative *IbbHLH* genes were identified. Whereafter, they were named from *IbbHLH1* to *IbbHLH227* based on their location on the 15 chromosomes of sweetpotato (Additional file 1), and we encourage to use this nomenclature in future reports concerning bHLH TFs.

Subsequently, the physicochemical properties including molecular weights (Mw), isoelectric points (PI), and phosphorylation sites of 227 IbbHLHs were detected. The number of amino acid residues ranged from 91 to 1161, and Mw changed from 9780.1 to 128668.66, and PI distributed from 4.68 to 11.82. Additionally, the potential phosphorylation site predictions displayed that IbbHLHs contained 8 (IbbHLH15) to 148 (IbbHLH147) possible phosphorylation sites (Additional file 2).

Chromosome mapping showed that *IbbHLHs* were mapped to all 15 sweetpotato chromosomes. Chr 11, Chr 2 and Chr 3 contain the largest number of *IbbHLHs*, with 31, 23 and 22 members, respectively, while Chr 8 has only 3 members. In addition, the two sets of chromosomes, Chr 10 and Chr 11, Chr 14 and Chr 15, contained the same number of genes, with 10 and 16 members, respectively. The data suggest that the distributions of *IbbHLHs* are disproportionate among sweetpotato chromosomes (Additional file 1).

### Phylogenetic analysis of IbbHLHs in sweetpotato

**Fig. 1:**
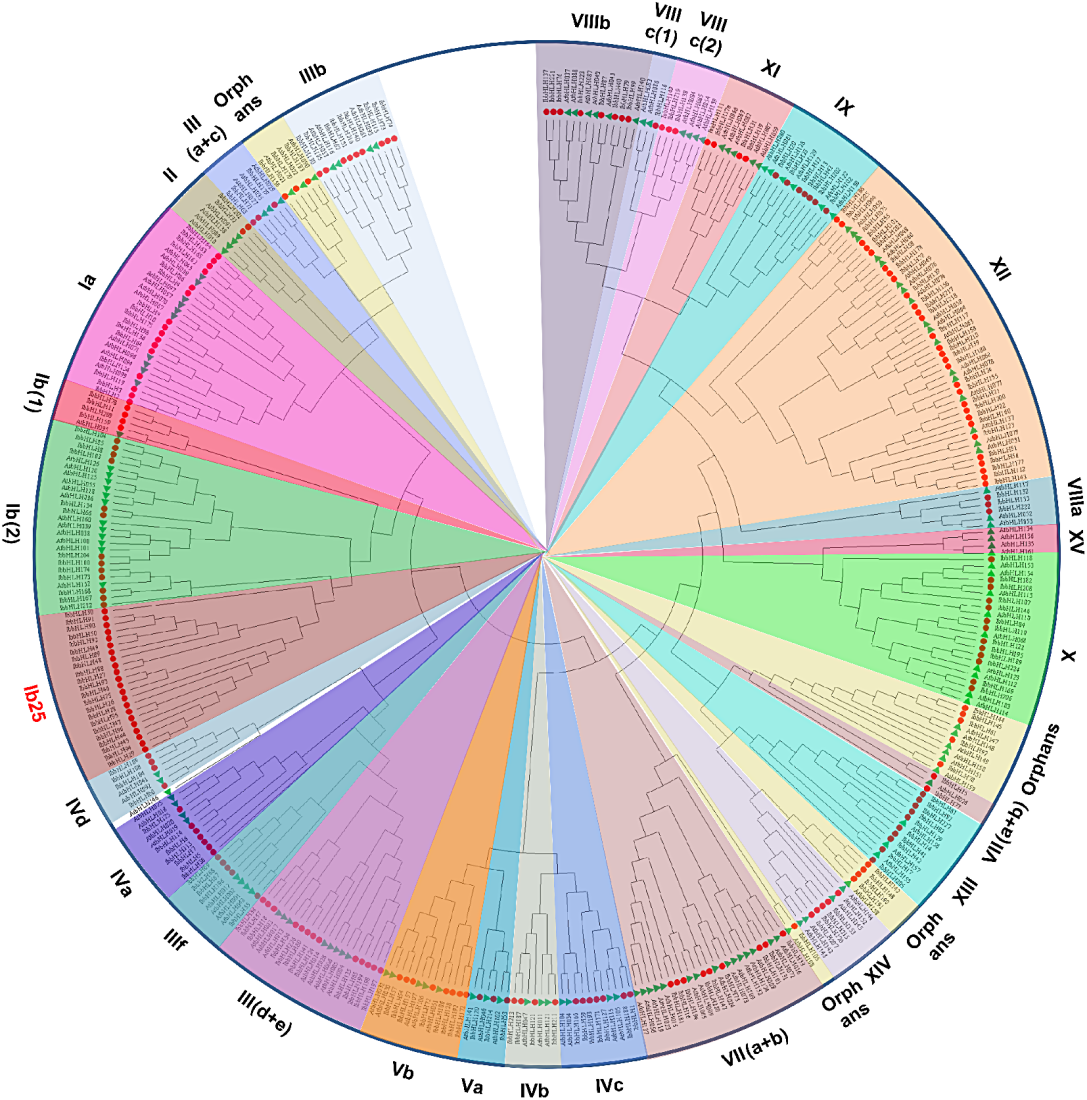
Phylogenetic tree representing the relationships between sweetpotato IbbHLHs and Arabidopsis AtbHLHs. The phylogenetic relationships were derived through the Maximum Likelihood method and the best evolutionary model JTT + G + F was employed with the bootstrap value of 1000. Different subgroups are named based on the reports in Arabidopsis, and are distinguished with different colors. The red circles and green triangles represent the sweetpotato IbbHLHs and Arabidopsis AtbHLHs, respectively

To study the evolutionary connection of IbbHLHs, a un-rooted phylogenetic tree was obtained by the complete aa information of 227 IbbHLHs and 162 Arabidopsis AtbHLHs (Additional file 3). In the previous study, 162 AtbHLHs were divided into 26 subfamilies [35]. Based on the report, a total of 27 subgroups were detected, and 227 sweetpotato IbbHLHs were divided into 26 subgroups. No sweetpotato IbbHLH members were found in the subfamily XV previously reported in Arabidopsis. And 22 IbbHLH members such as IbbHLH25, IbbHLH45 and IbbHLH95 were divided into a new sweetpotato-specific subfamily named Ib25. The results show that the distribution of IbbHLHs in different subfamilies has considerable diversity and inhomogeneity (Fig. 1).

### Exon-intron structure and conserved domain analyses of *IbbHLHs*

**Fig. 2:**
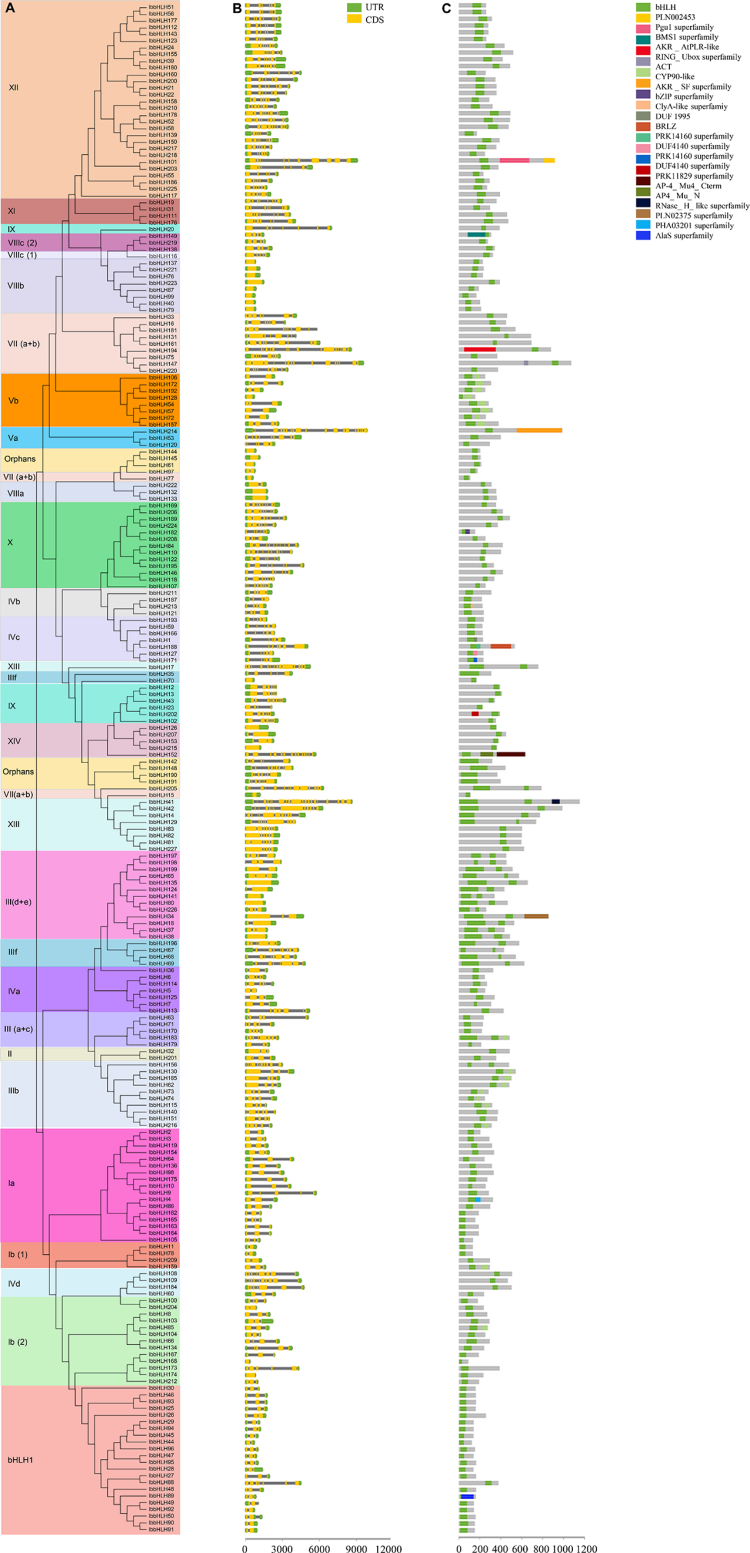
Phylogenetic relationships, gene structures and conserved domains in sweetpotato IbbHLHs. (**A)**. The phylogenetic tree of 227 IbbHLHs was constructed by MEGA X based on the same parameters used in Fig. 1. Different subgroups and their IbbHLH members are displayed in different colors. (**B**) Sketch map of gene structures of 227 *IbbHLH*s. CDS and UTR are indicated using yellow and green bars, respectively. (**C)** Distributions of conserved domains in the IbbHLHs based on the phylogenetic relationships. Boxes of different colors present different domains, and the green box contained in each IbbHLH represents the bHLH domain

Batch CD-Search results of 227 IbbHLH proteins revealed that all IbbHLHs contained a highly conserved bHLH domain as expected. In addition, eight IbbHLH members of the Vb subfamily contain the additional CYP90-like domain except the conserved bHLH domain (Fig. 2 and Additional file 4). Exon-intron structure detection revealed that the exon numbers of *IbbHLH* genes varied from 1 to 20, of which 25 *IbbHLHs* lacked introns, and most *IbbHLHs* had one to nine exons. Among them, *IbbHLH41* has the highest number of exons with 20, followed by *IbbHLH214* and *IbbHLH194* with 19 and 17, respectively. Moreover, our results illustrated that the number and length of exons of most *IbbHLHs* in the same subgroup are generally similar. For instance, all members of the VIIIb subgroup contain only one exon (no introns), and most members of the Ia subfamily contain three exons, with the exception of *IbbHLH-2/-9/-105/-165* (Fig. 2).

To analyze the similarities and differences in protein structure of sweetpotato IbbHLHs, the conserved motifs of 227 bHLHs were established by MEME software. Then 20 motifs were doped out, and the results suggested that motif 1 represented the loop and the second helix region, existing in 95% of the IbbHLH proteins. Moreover, most IbbHLHs in the same subgroup generally exhibited similar motif compositions. For instance, motifs 1, 7, 3, 10, and 18 coexisted in all members of Ib25 subfamily. Most members of the XIII subfamily contained motif 5. Motifs 1 and 2 form the bHLH domain that is present in most of the IbbHLH. The data suggest that there are obvious differences in motif composition between different bHLH subfamilies, and the specific motifs in some subgroups may imply the unique and different roles of related *IbbHLH* genes (Additional file 5).

### Gene duplications and collinearity analyses of sweetpotato *IbbHLHs*

**Fig. 3:**
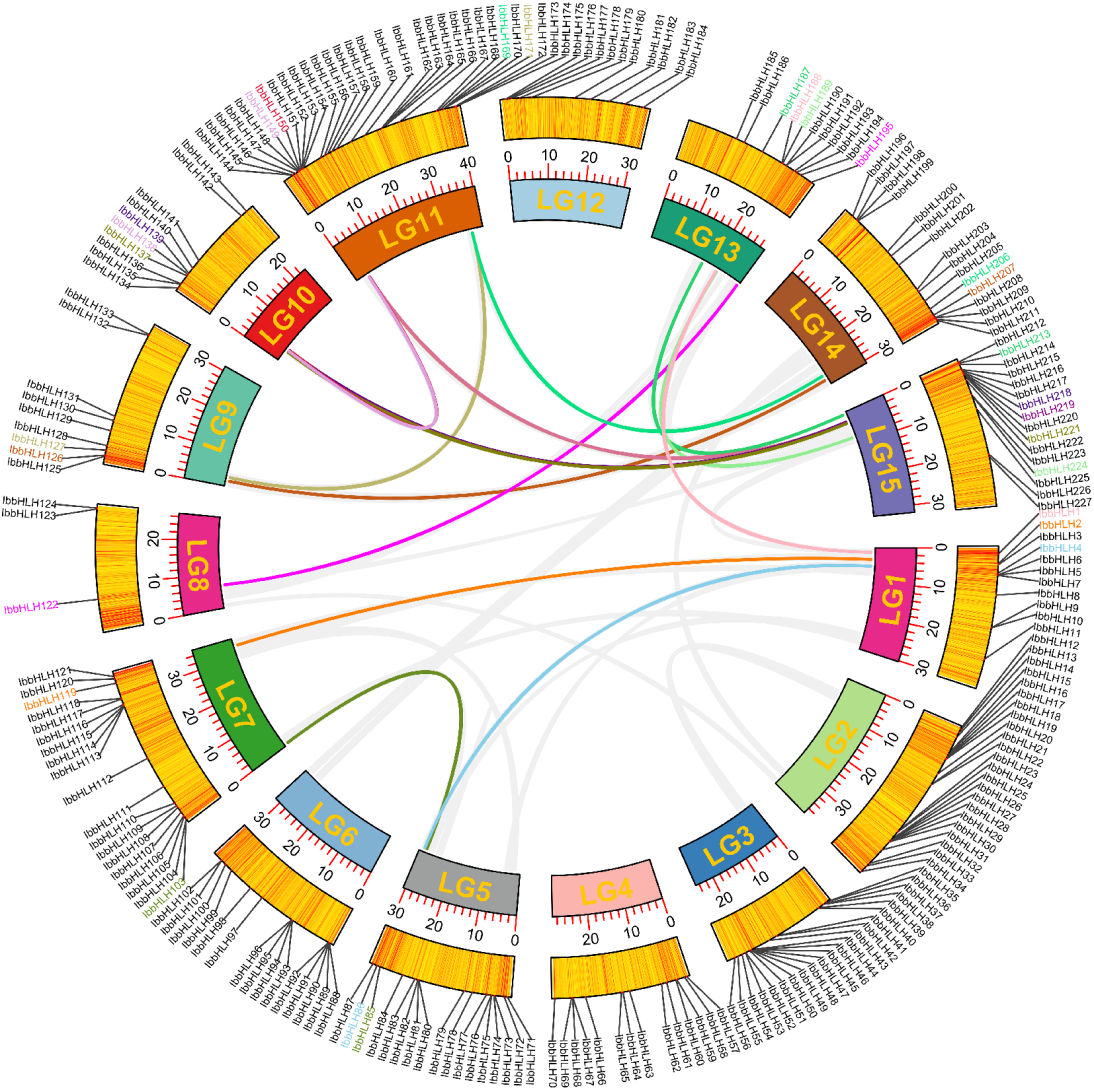
Localizations and segmental duplications of *IbbHLH* genes in the sweetpotato chromosomes. Circular visualizations of *IbbHLH* genes mapped to the LG1-LG15 chromosomes are indicated by colored rectangles. The gene densities on each chromosome are depicted by the polyline along each rectangle. Duplicated *IbbHLH* gene groups in sweetpotato chromosomes are represented by colored lines, and these genes are also marked with different colors

Genome duplications promote the evolution and expansion of many genes [36]. To explore the potential duplication events among the 227 *IbbHLH* genes, gene duplication and collinear analysis were conducted. The data showed that seven pairs of tandem duplicated *IbbHLHs* were detected, including *IbbHLH5*-*IbbHLH6*, *IbbHLH25*-*IbbHLH26*, *IbbHLH49*-*IbbHLH50*, *IbbHLH103*-*IbbHLH104*, *IbbHLH162*-*IbbHLH163*, *IbbHLH164*-*IbbHLH165*, *IbbHLH173*-*IbbHLH174* (Additional file 1). Additionally, 17 segmental duplicate gene pairs were identified using the BlastP and MCScanX programs on 10 of the 15 chromosomes as follows: *IbbHLH1*-*IbbHLH118*, *IbbHLH2*-*IbbHLH119*, *IbbHLH4*- *IbbHLH86*, *IbbHLH85*-*IbbHLH103*, *IbbHLH122*-*IbbHLH195*, *IbbHLH126*-*IbbHLH207*, *IbbHLH127*-*IbbHLH171*, *IbbHLH137*-*IbbHLH221*, *IbbHLH138*-*IbbHLH-149*/-*219*, *IbbHLH150*-*IbbHLH-139*/-*218*, *IbbHLH169*-*IbbHLH206*, *IbbHLH187*-*IbbHLH213* (Fig. 3 and Additional file 6).

### Synteny analysis of *bHLH* genes between sweetpotato and other plants

**Fig. 4:**
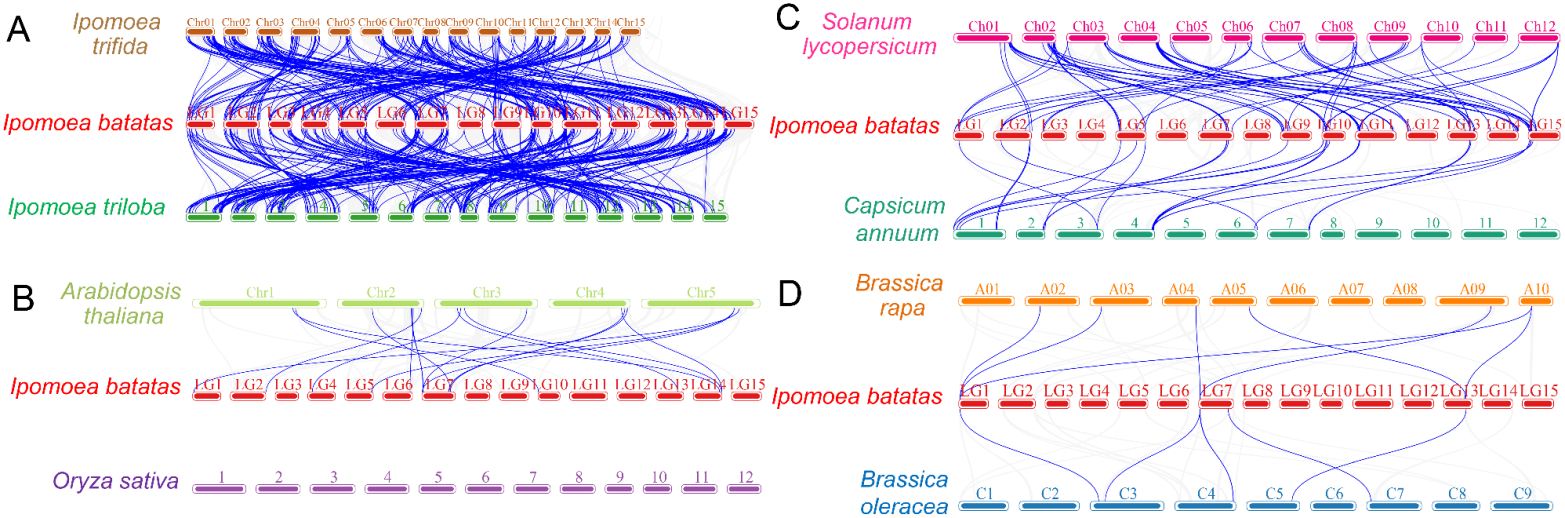
Collinearity analyses of bHLH genes between sweetpotato and the eight representative plant species from *I. trifida* and *I. triloba* (**A**), Arabidopsis and rice (**B**), tomato and pepper (**C**), cabbage and *Brassica oleracea* (**D**). The chromosomes of different plants are distinguished with differential colors. The blue lines connecting two different chromosomes indicate all the syntenic bHLH gene pairs within sweetpotato and other plant genomes

Synteny connections between sweetpotato *IbbHLHs* and orthologous genes from eight plants including *I. trifida*, *I. triloba*, *Arabidopsis, Oryza sayiva*, *Brassica rapa*, *Brassica oleracea*, *Solanum lycopersicum*, and *Capsicum annuum*, were analyzed to diagnose the evolution of *IbbHLHs*. The results showed that there were 174 and 178 homologous genes between sweetpotato and *I. trifida* and *I. triloba*, respectively. And 16, 5, 7, 65 and 26 syntenic relationships were found between sweetpotato and *Arabidopsis thaliana*, *Brassica oleracea*, *Brassica rapa*, *Solanum lycopersicum*, and *Capsicum annuum*, respectively. However, there was no homologous gene was detected between sweetpotato and rice. Additionally, we also found that 89 genes from *I. triloba* and 86 genes from *I. trifida* were collinear with two or three *IbbHLHs*, such as: *itb02g11420.t1*-*IbbHLH-1*/-188, *itb06g15350.t1*-*IbbHLH-224/-18* (Fig. 4 and Additional file 7). The data suggest that sweetpotato has the most homologous genes with *I. trifida* and *I. triloba*, probably because they are the likely diploid wild relatives of sweetpotato.

### Screening of salt-responsive *IbbHLHs* by transcriptome analysis and their expressions under abiotic stress

To explore the possible roles of *IbbHLHs* in stress response, their transcription patterns under salt stress were analyzed using our previous transcriptome data [37]. The results displayed that many of the screened *IbbHLHs* were salt-responsive (Additional file 8). In order to confirm the expression data, the transcription levels of 12 selected *IbbHLHs* that displayed significant change in the RNA-seq data were detected under different abiotic stresses using qRT-PCR assays. It is worth noting that sweetpotato has complex transcripts as a hexaploid crop, the genome sequences of different varieties are diverse. The protein sequences of stress-related *IbbHLHs* screened in XuShu22 are not exactly the same as the 227 identified *IbbHLHs* in Taizhong6. Consequently, for the homologous bHLH sequences between the two sweetpotato varieties, if their aa sequence similarity is less than 95%, it will be named as the corresponding bHLH-like gene. In order to rigorously identify the biologically significant *IbbHLH* genes, the two-fold threshold was used [38].

**Fig. 5:**
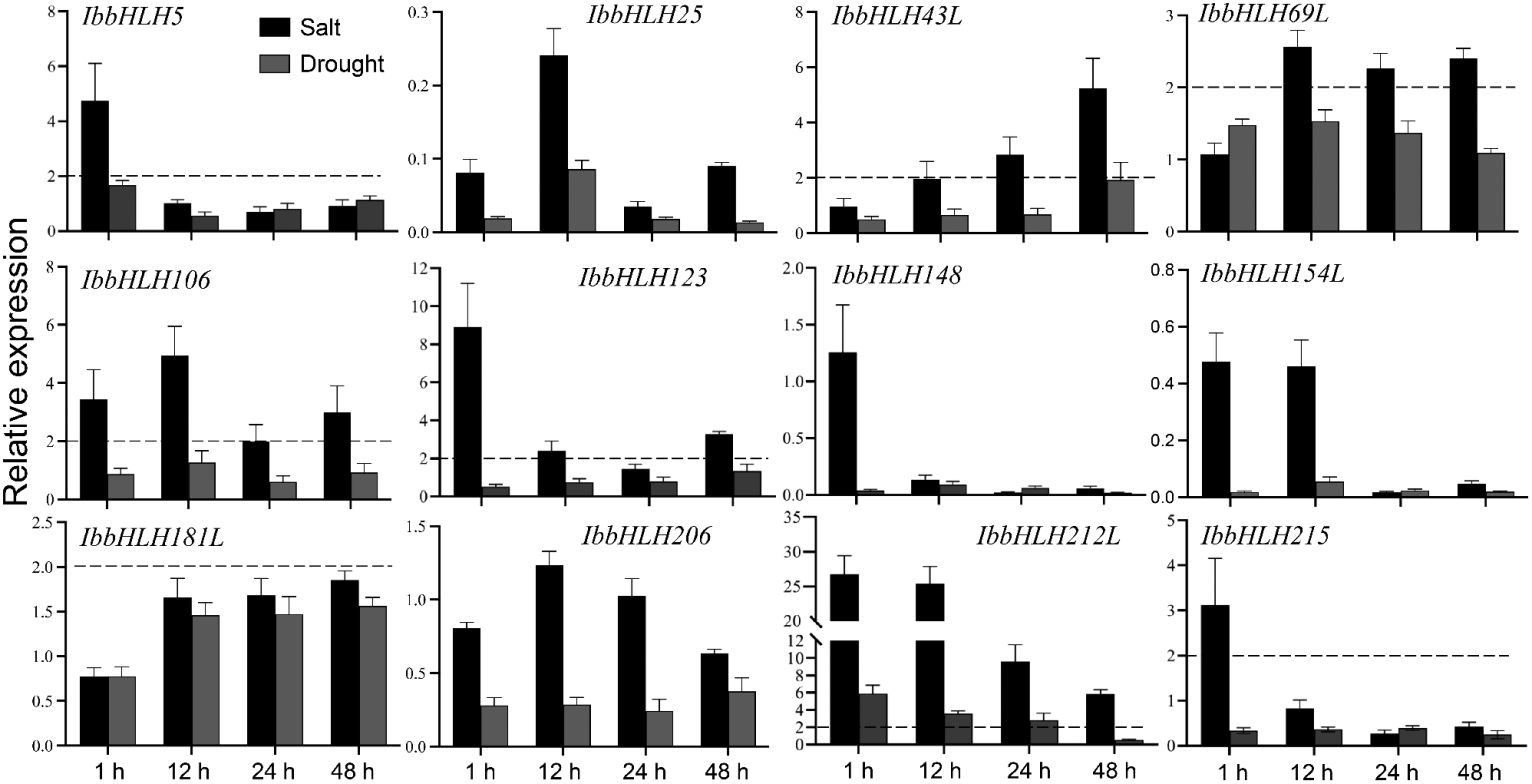
Relative expression levels of 12 *IbbHLHs* after abiotic stress treatments were detected by qRT-PCR. The abiotic stress treatments include salt (150 mM NaCl) and drought (20% PEG6000). The Y-axis delineates the fold changes of relative expression comparing with 0 h (data was normalized to 1). Bars represent the mean of three biological replicates ± SE. The two-fold threshold is presented by a dotted line

The qRT-PCR results have good consistency with the transcriptome data, most of the detected *IbbHLHs*, except *IbbHLH-25/-148/-154 L/-181 L/-206*, showed significant and differential expression levels after salt or PEG6000 treatments. The expression of *IbbHLH-5/-123/-215* reached the highest after salt treatment for 1 h, which was 3-9-fold changes. The transcription of *IbbHLH43L* showed an upward trend with the increase of time under salt treatment, and the expression of *IbbHLH69L* and *IbbHLH106* reached the highest level after 12 h of salt treatment. The expression of *IbbHLH212L* displayed the highest upregulation level under salt and drought treatments, proximately 27- and 6-fold, respectively (Fig. 5).

### Detection of cis-elements in the *IbbHLH* gene promoters

To analyze the possible regulatory mechanisms of *IbbHLHs* under abiotic stress, the cis-elements in the promoter sequences of *IbbHLHs* were predicted. The results exhibited that about 80% of the sequences contained one or more stress-related elements, such as drought responsive element (MBS) and defense and stress responsive element (TC-rich repeats) (Additional file 9). These cis-elements might be associated with the upregulated expression of *IbbHLHs* under abiotic stress. For instance, the expression levels of *IbbHLH-106/-123/-212 L* were increased under salt or PEG6000 stresses, and correspondingly, MBS and TC-rich repeats elements appeared in their promoters (Additional file 10).

Besides, 98% of the promoter regions of *IbbHLHs* also have one or more hormone-associated cis-elements, such as abscisic acid responsive elements (ABRE), auxin responsive elements, and salicylic acid responsive elements (Additional file 9). The results showed that *IbbHLH-43*/-*123*/-*212* contained three or more ABREs, indicating that they might participate in abiotic stress response. Additionally, the promoters of 18 *IbbHLHs* were also found to contain flavonoid biosynthetic response elements (MBSI), suggesting that they may be related to flavonoid synthesis (Additional file 10).

### Detection of transactivation activity and protein interaction of selected IbbHLHs

**Fig. 6:**
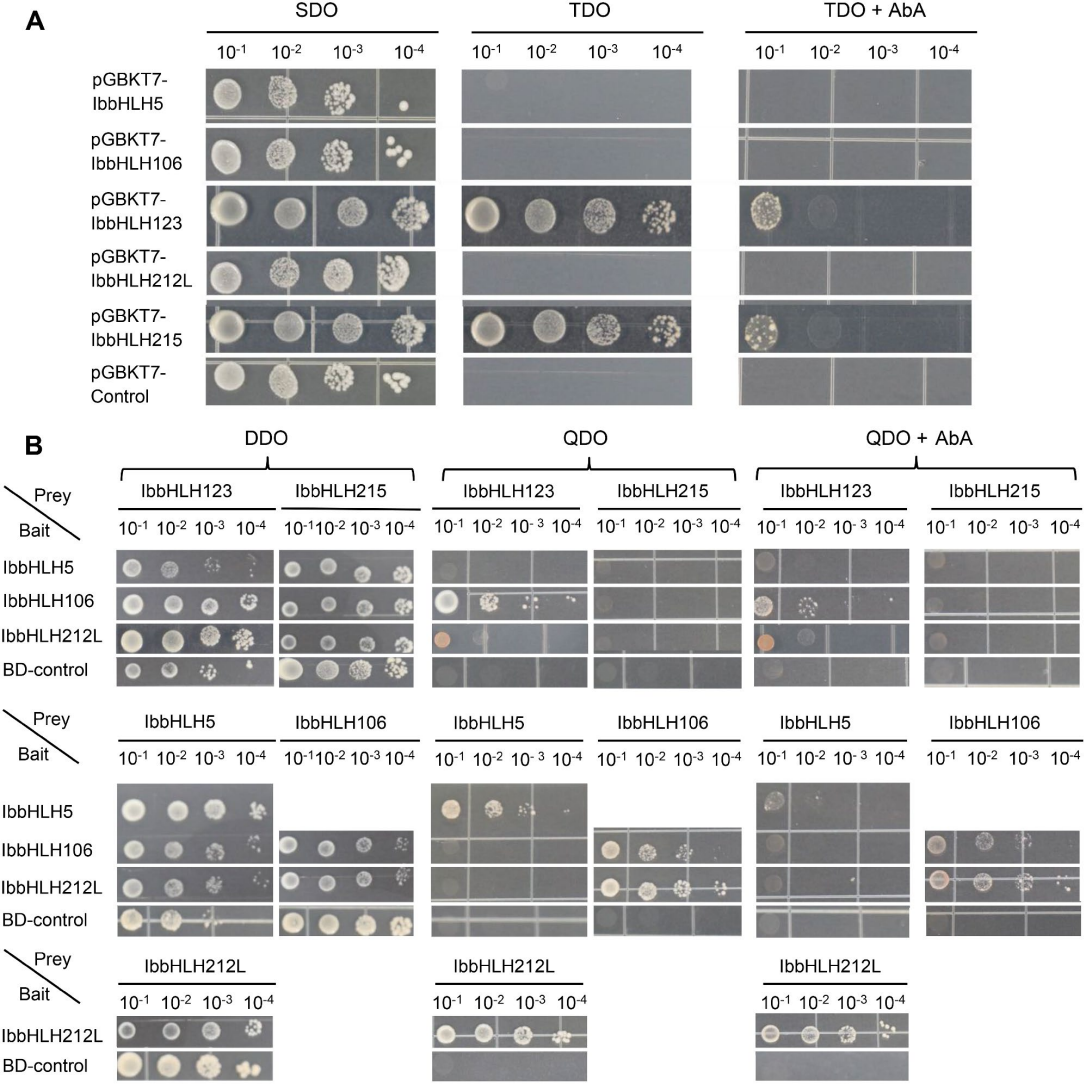
Analysis of transactivation activity and protein interaction of IbbHLH proteins. (**A)** Yeasts containing pGBKT7-IbbHLH-5/-106/-123/-212 L/-215 or pGBKT7 empty vector were streaked on the SDO (SD medium lacking Trp); TDO (SD medium lacking Trp, His, Ade) and TDO medium with 200 ng/mL AbA. (**B)** Yeasts containing both the indicated recombined pGBKT7 and pGADT7 plasmids were streaked on DDO (SD/-Trp-Leu) medium, QDO (SD/-Trp-Leu-His-Ade) medium with or without 200 ng/mL AbA. All the plates were recorded 3 d after 30° of incubation

The expression of *IbbHLH-5/-106/-123/-212 L/-215* was significantly upregulated by abiotic stress, thus these were selected to further examine the molecular characteristics. First, the five recombinant pGBKT7-*IbbHLH* plasmids were transformed into yeast cells to detect their potential transactivation activities. The results exhibited that only the transformed yeasts containing recombinant IbbHLH123 or IbbHLH215 plasmids can normally grow on TDO and TDO + AbA medium, suggesting that IbbHLH123 and IbbHLH215 had transactivation activities (Fig. 6A).

Besides, it has been reported that bHLH TFs often participate in stress responses by forming complexes [8, 10]. Subsequently, the potential interactions between any two of the five IbbHLHs were evaluated by yeast two-hybrid assays (Y2H), except IbbHLH123 and IbbHLH215 due to their transactivation activities. The data confirmed that IbbHLH5 could interact with itself, IbbHLH106 could interact with IbbHLH-123/-212 L in addition to itself, and IbbHLH212L could interact with IbbHLH123 and itself, while no visible interactions were observed in other combinations (Fig. 6B).

Moreover, to systematically explore the complex relationships among the IbbHLHs, the potential interaction networks of IbbHLHs were established according to the orthologs of Arabidopsis AtbHLH proteins. The findings also suggested that the complicated complexes could be formed between multiple IbbHLHs, such as bHLH104 (IbbHLH-59/-166) and bHLH115 (IbbHLH127), EGL3 (IbbHLH67) and GL3 (IbbHLH-68/-69/-196) (Additional file 11). The data suggest a promising protein interaction connection, indicating the potential way for sweetpotato IbbHLHs in regulating the response to abiotic stress.

### Transcriptome-wide identification of *IbbHLHs* related to anthocyanin synthesis and their expression profiles in different sweetpotato cultivars

bHLH TFs were widely reported to participate in the modulation of anthocyanin synthesis in many plants [20]. IbbHLH2 (JQ337863) was reported to coordinate with IbERF71 and IbMYB340 to regulate anthocyanin synthesis in sweetpotato [34], and our previous expression quantitative trait locus (eQTL) and coexpression analysis also revealed that the homology gene (itf14g18730.t1) of *IbbHLH2* in *I. trifida* might be involved in flavonoid biosynthesis [39]. It is worth mentioning that we did not identify a protein highly consistent with the IbbHLH2 in the genome of Taizhong6 (non-purple cultivar), which may be one of the reasons for its lack of anthocyanin accumulation. However, the protein sequences encoded by *IbbHLH35* and *IbbHLH36* were found to be the N-terminal and C-terminal of IbbHLH2, respectively, and in order to distinguish, we have marked the accession number for the previously reported IbbHLH2.

To explore new *IbbHLH* genes that may be related to anthocyanin accumulation, the tuberous roots of purple-fleshed (LZ4) and white-fleshed (QS12) cultivars were used for transcriptome screening. Then 34 differentially expressed *IbbHLH* genes were identified in LZ4 vs. QS12 analysis (Additional file 12). Moreover, for a more comprehensive identification of potential anthocyanin synthesis-related *IbbHLHs*, our previous eQTL analysis and previously published available RNA-seq data were also referenced [39, 40]. Subsequently, the expression of 12 *IbbHLHs* between the purple flesh and white skin of three sweetpotato cultivars was detected by qRT-PCR, and *IbbHLH2* (JQ337863) was used as a reference gene to assist in evaluating the expression data.

**Fig. 7:**
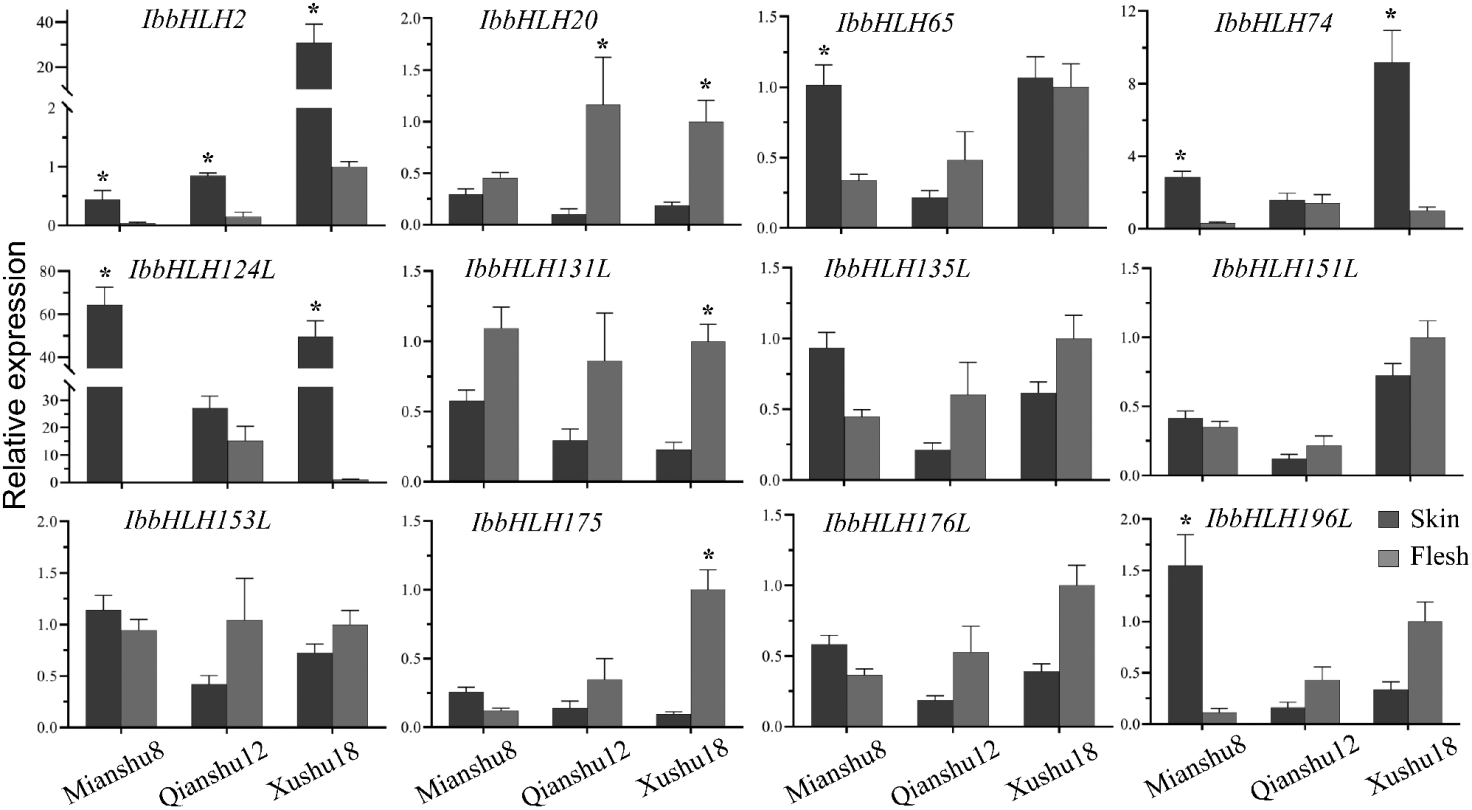
The relative expression levels of 11 *IbbHLHs* between flesh and skin tissues of three sweetpotato cultivars were detected by qRT-PCR. The Y-axis depicts the change in the relative expression compared to the flesh of XuShu18 (data normalized to 1). The bars represent the average of the three biological replicates ± SE. The three-fold threshold between flesh and skin tissues is represented by an asterisk

To strictly screen the *IbbHLHs* associated with anthocyanin synthesis, a threshold of three-fold for differential gene expression was employed. The data displayed that the transcriptions of the reference gene *IbbHLH2* (JQ337863) were specifically accumulated in purple skin tissues as expected, similar expression profiles were also detected in the transcription levels of *IbbHLH74* and *IbbHLH124L*, indicating that they may participate in the regulation of anthocyanin synthesis. Additionally, *IbbHLH65* and *IbbHLH196L* were also mainly expressed in purple skin tissues, contrarily, *IbbHLH20*, *IbbHLH131L*, and *IbbHLH175* were mainly accumulated in the white flesh, although their expression levels were different among sweetpotato varieties (Fig. 7).

## Discussion

Massive documents have demonstrated that bHLH TFs play important roles in signal transduction networks that modulate diverse physiological and biological processes of plants, including growth and stress tolerance [6, 8, 11]. Sweetpotato has significant advantages in adapting to the environment, which is of great significance to ensure food safety in many countries [29]. However, comprehensive information on the sweetpotato *bHLH* gene family is still scarce. Previously, 110 *IbbHLHs* were identified in sweetpotato genomes [31]. In this study, a more systematic and comprehensive identification of *IbbHLHs* in sweetpotato was carried out from various aspects. A total of 227 *IbbHLHs* with obvious variabilities were isolated in sweetpotato, and the potential *IbbHLHs* involved in abiotic stress response and anthocyanin biosynthesis were screened and identified by transcriptome sequencing, qRT-PCR, and yeast two-hybrid experiments.

The number of 227 *IbbHLHs* was higher than that of many other plants including the 142 in cucumber [15], 161 in tomato [41], 162 in Arabidopsis [42], 167 in rice [7], and 208 in maize [43], but less than in the 230 in Chinese cabbage [44], and 602 in *Brassica napus* [45]. This illustrates that there is a clear difference in the size of bHLH genes between monocots and dicots. Additionally, 227 *IbbHLHs* were found to map on all the 15 chromosomes of sweetpotato, while the number of *IbbHLHs* did not correspond to the chromosome sizes. Similar distributions in cucumber [15], sweet cherry [46], orchardgrass [47], and sweet osmanthus [48] were also found.

Based on the phylogenetic tree analysis, 227 IbbHLHs were divided into 26 subgroups including a new sweetpotato-specific subfamily, indicating a potential specific function of these IbbHLHs. The analysis of protein conserved motifs and exon-intron structures showed that members of the same subgroup still shared similar conserved motifs and gene structures, indicating that different members of the same subgroup might have a common evolutionary origin, which provided basic references for their phylogenetic relationships and functional relevances [49]. Gene duplication events promote gene expansion and plant evolution [36]. Seven groups of tandem duplicated *IbbHLHs* and 12 groups of fragment duplicated *IbbHLHs* were found by collinear analysis, suggesting that fragment duplications were the dominant driving force for their expansions. The results were consistent with the *bHLH* genes in pear [50], orchardgrass [47], *Cinnamomum camphora* [51], and *Ficus carica* [52]. The data further confirm the common mechanism by which segmental duplication or genome duplication contributes to *bHLH* gene expansions reported in other plants.

Many documents exhibited that bHLH TFs play an inestimable role in plant stress resistance. For instance, Arabidopsis AtbHLH112 could regulate the expression of stress-related genes to enhance salt and drought tolerance [13]. Heterologous of *MfbHLH38* in Arabidopsis improved salt and drought tolerance by enhancing ROS scavenging ability and promoting the sensitivity of stomatal closure [14]. And overexpression of *SlbHLH22* improved the salt and drought tolerance in tomato [18]. At present, the function of bHLH TFs in sweetpotato was poorly studied, our RNA-seq and qRT-PCR results showed that the transcriptions of many *IbbHLHs* were remarkably induced by salt and drought stress, including *IbbHLH-5/-43 L/-106/-123/-206/-212 L*, implying that sweetpotato IbbHLH TFs might participate in the response to abiotic stress. Besides, lots of stress- and hormone-related cis-elements were found in the promoter sequences of *IbbHLH* genes, further consolidating that *IbbHLHs* are potential candidates for sweetpotato genetic engineering under environmental stresses, while the specific functions of these *IbbHLHs* remain to be revealed.

Heterodimers can be formed between different bHLH TFs, and bHLH proteins can also heterodimerize with other TFs, including MYB/BZR1-BES1 and other signal transduction factors [8]. Previous results have exhibited that bHLH29 could interact with bHLH-38/-39/-100/-101 to form heterodimers, and bHLH34, bHLH104, bHLH105, and bHLH115 could form dimers to modulate Fe homeostasis [9]. Herein, our assays showed that sweetpotato IbbHLH TFs might also be involved in the stress response via the intricate protein interactions. Collectively, our results suggest that stress-related IbbHLH proteins form an involute complex through protein-protein interactions, which plays a critical role in integrating abiotic stress signals.

Besides, massive documents have shown that bHLH TFs function as key regulators of anthocyanin synthesis in many plants, and anthocyanin synthesis is regulated by the MBW complex [25, 26]. Previously, sweetpotato *IbbHLH2* (JQ337863) was reported to regulate anthocyanin accumulation by the IbMYB340-IbbHLH2-IbNAC56 and IbERF71-IbMYB340-IbbHLH2 transcriptional complex [34, 53]. Our qRT-PCR data showed that *IbbHLH2* was significantly accumulated in the purple tissues, which was consistent with the previous reports. And *IbbHLH74*, *IbbHLH124L* and *IbbHLH196L* were also specifically accumulated in purple skin tissues. In addition, the phylogenetic analysis indicates that IbbHLH196 belongs to the subfamily IIIf, and most members of the subfamily IIIf are closely related to the synthesis of anthocyanins, such as the *Freesia hybrida FhGL3L* and *FhTT8L* of IIIf subfamily are associated with flavonoid biosynthesis [54]. The *Chrysanthemums CmbHLH2* of subfamily IIIf binds to the promoter of *CmDFR* and interacts with CmMYB6 to regulate anthocyanin synthesis, giving flowers a variety of colors [55]. The results suggest that these *IbbHLHs* may be closely related to the regulation of anthocyanin synthesis, while additional experimental confirmation is needed.

## Conclusions

In this study, 227 *IbbHLHs* were defined in sweetpotato genomes, and their chromosome mapping, phylogenetic relationship, as well as exon-intron structure, conserved motif, and syntenic analysis were conducted. Segmental duplications were identified as the dominant driving force for the expansion of *IbbHLHs*, and syntenic analysis between sweetpotato and eight plants supplied valuable clues to the evolution of *IbbHLHs*. The transcription of many *IbbHLHs* was evidently upregulated by abiotic stress according to the transcriptome data and qRT-PCR analysis, supporting the prospect that multiple *IbbHLHs* possess promising regulators in enhancing sweetpotato stress tolerance. In addition, IbbHLH123 and IbbHLH215 have obvious transactivation activities in yeasts, and a complex protein interaction network among IbbHLHs was identified by Y2H and STRING analysis, suggesting the complicated connections and regulatory mechanisms for IbbHLHs in regulating stress response. Besides, transcriptome screening and qRT-PCR detection revealed that multiple *IbbHLHs* may be closely related to the regulation of anthocyanin biosynthesis. Collectively, these results can promote the understanding of the complexity of sweetpotato *bHLH* gene family, and shed light on the promising functions of *IbbHLHs* in abiotic stress response and anthocyanin biosynthesis.

## Methods

### Isolation of *IbbHLHs* in the sweetpotato genome

The genome and the corresponding annotation files of sweetpotato (*Ipomoea batatas* L.) were received in the *Ipomoea* Genome Hub database (https://ipomoea-genome.org) [30]. To obtain all the potential bHLH genes, two ways were carried out. Frist, the HMM file (PF00010) with 141 seed sequences (Additional file 13) was downloaded from Pfam database (http://pfam.xfam.org/) as queries to search the entire protein database of sweetpotato by the BLASTP program with a threshold E-value of 1e^− 5^. Second, the reported bHLH protein sequences of Arabidopsis and rice (Additional file 3) were downloaded from the TAIR (https://www.arabidopsis.org/) and Rice Genome Annotation Project (http://rice.plantbiology.msu.edu/), then they were also employed as queries to retrieve the possible bHLH proteins using the BLASTP program with a threshold E-value of 1e^− 5^. Afterwards, the redundant protein IDs and sequences were eliminated using the remove duplicate tool of Excel, and all the obtained members were further examined by NCBI (https://www.ncbi.nlm.nih.gov/) and Pfam databases. Finally, 227 non-redundant sequences were confirmed as putative sweetpotato bHLH proteins and used for the downstream analysis. And 10 sequences were identified as IbbHLH-related proteins. The sequence information of 227 IbbHLHs and 10 IbbHLHs-related sequences is presented in Additional file 14.

### Multiple alignment and phylogenetic relationship analysis of IbbHLHs

Complete protein sequences of 227 sweetpotato IbbHLHs and 162 Arabidopsis AtbHLHs were used to construct the phylogenetic relationship. Sequence alignment was carried out by the ClustalW software using the default parameters, and the generated information was applied to perform the phylogenetic analysis through the Maximum Likelihood method by the MEGA software (X version) [56] with the best JTT + G + F model, and the bootstrap value was set to 1000. Simultaneously, the same parameters were used for the phylogenetic analysis of 227 sweetpotato IbbHLH proteins, and bHLHs was classified according to the report of *Arabidopsis* AtbHLHs [35].

### Physicochemical properties and characterization of IbbHLHs

The ExPASy database (http://expasy.org/) was applied to calculate the physicochemical properties of 227 IbbHLH proteins with the default parameters. The WoLF PSORT online website (https://wolfpsort.hgc.jp/) was employed to forecaste their subcellular localizations.

### Analyses of gene structure, conserved domain and protein interaction network

The exon-intron structure was plotted by the GFF annotations for 227 *IbbHLHs*, then the results were demonstrated by the TBtools software (v1.0971) [57]. Conservative motifs were showed by MEME 5.4.1 program (https://meme-suite.org/meme/tools/meme) using the following parameters: maximum number of motif: 20, maximum motif width: 100, minimum motif width: 6, other parameters were default. Moreover, the potential protein interaction network was forecasted by STRING 11.0 (https://string-db.org/) with the parameters left at their default values.

### Chromosome localization and syntenic analysis of *IbbHLHs*

The *IbbHLHs* were mapped onto sweetpotato chromosomes based on the GFF annotations. The genome and annotation information of *I. trifida*, *I. triloba*, *Arabidopsis*, *Oryza sativa*, *Solanum lycopersicum*, *Capsicum annum*, *Brassica rapa*, and *Brassica oleracea* were obtained from different databases such as TAIR, Phytozome (https://phytozome.jgi.doe.gov/pz/portal.html), and Ensembl (http://plants.ensembl.org/index.html). The collinearity detection between 227 *IbbHLHs* and the orthologous genes from these plants were calculated through the MCScanX software with the default parameters, and the results were rendered using Circos and TBtools softwares with a minimun block size set to 30 [58, 59].

### Transcriptome screening and qRT-PCR verification of *IbbHLH* genes related to salt stress response and search for cis-elements in promoters

Differential *IbbHLH* expression analysis under salt stress was performed using the transcriptome data as the descriptions in our previous publication [37]. Gene expression was estimated by read counts by false discovery rate (FDR) [60] and Log2 (fold change). The differentially expressed genes were annotated based on multiple databases, such as Nr, Pfam, and SwissProt databases. The abiotic stress treatments were conducted as described before [61]. Briefly, salt stress and drought stress were performed by immersing the root of Xushu22 seedlings (obtained from the Xuzhou Sweetpotato Research Center, China) into 150 mM NaCl and 20% PEG6000, respectively, and adventitious roots at specified time points, including 1, 12, 24 and 48 h were collected, respectively. For each stress, three independent biological replicates were sampled. No permission was necessary to collect the plant materials.

Total RNA of each sample was isolated by the RNA Extraction Kit (TianGen, Beijing, China) followed the manufacturers’ instruction. 1 ug RNA of each sample was reverse transcribed by PrimeScript reverse transcriptase with gDNA Eraser (TaKaRa). The qRT-PCR experiment was carried out using the CFX96™ system (Bio-Rad, USA) with the processes described earlier [61], and sweetpotato *ARF* gene was applied as an internal reference [62]. The related primers are found in Additional file 15. In addition, to investigate the cis-elements in the promoter regions of 227 *IbbHLHs*, 2 kb sequence of each promoter was uploaded to the plant CARE database (http://bioinformatics.psb.ugent.be/webtools/plantcare/html/) for detection.

### Detection of transactivation activity and protein interaction of IbbHLHs

The open reading frames of selected *IbbHLH-5/-25/-69 L/-106/-123/-43 L/-148/-154 L/-181 L/-206/-212 L/-215* were cloned and then fused into pGBKT7 or pGADT7 vectors by ClonExpress II (Vazyme, Nanjing, China) recombination reaction. The empty pGBKT7, each recombinant pGBKT7-IbbHLH, and recombinant pGBKT7-IbbHLH and pGADT7-IbbHLH plasmids were then subjected to Y2HGold yeast transformation [63, 64]. Yeast dilutions were then dropped on SD/-Trp, SD/-Trp-His-Ade medium with or without AureobasidinA (AbA) for transactivation activity analysis. Yeast dilutions were dropped on SD/-Trp-Leu, SD/-Trp-Leu-His-Ade medium with or without AbA for protein interaction detection. All the plate was incubated upside down at 28 °C for three days to detect the growth phenotype of yeasts. The related primers are found in Additional file 16.

### Transcriptome screening and qRT-PCR verification of *IbbHLH* genes related to anthocyanin biosynthesis

The flesh of Luozi4 (LZ4, purple-fleshed cultivar) and Qianshu12 (QS12, white-fleshed cultivar) was collected from guizhou academy of agricultural sciences for transcriptome sequencing with three biological replicates conducted by Metware Co., Ltd (Wuhan, China) as described previously [65]. No permission was necessary to collect the plant materials. Genes with |Log2 (fold change)| > 1 and padj < 0.05 found by DESeq were identified as DEGs (differentially Expressed Genes). Then the flesh and skin tissues of mature tuberous roots of three sweetpotato cultivars (Mianshu8, Xushu18, and Qianshu12) with purple skin and white flesh were collected for qRT-PCR assays to validate the transcriptome results as described above. Primers for qRT-PCR analysis are found in Additional file 15.

### Statistical analysis

To rigorously filter the biologically significant *IbbHLH* genes, a cut-off value of three-fold for anthocyanin-related detection and two-fold for analyzing stress induction were employed [38]. Graphpad prism 9 (www.graphpad.com) was applied to create figures.

### Electronic supplementary material

Below is the link to the electronic supplementary material.

Supplementary Material 1

Supplementary Material 2

Supplementary Material 3

Supplementary Material 4

Supplementary Material 5

Supplementary Material 6

Supplementary Material 7

Supplementary Material 8

Supplementary Material 9

Supplementary Material 10

Supplementary Material 11

Supplementary Material 12

Supplementary Material 13

## Data Availability

The datasets supporting the conclusions of this article are included within the article and its supplementary information files. The RNA-seq data used and analyzed during this study are available in the NCBI database (accession numbers SAMN14884352-SAMN14884363).
