# Supplementary material for: Systematic identification and expression analysis of bHLH gene family reveal their relevance to abiotic stress response and anthocyanin biosynthesis in sweetpotato
Source: BMC Plant Biol. 2024 Mar 1;24:156. doi: 10.1186/s12870-024-04788-0 (PMC10905920; doi:10.1186/s12870-024-04788-0)
Supplement: Supplementary file 2 — Supplementary Material 2 [file 12870_2024_4788_MOESM2_ESM.docx]

**Additional file 2**. Characteristics of 227 IbbHLH proteins in sweetpotato.

| Gene name | Gene ID | Amino acids | PI | MW | Subcellular  localization | No.of phosphorylation cite | | | |
| --- | --- | --- | --- | --- | --- | --- | --- | --- | --- |
|  |  |  |  |  |  | Ser site | Tyr cite | Thr cite | Total |
| *IbbHLH1* | g247.t1 | 233 | 7.63 | 25436.83 | Nucleus | 18 | 0 | 4 | 22 |
| *IbbHLH2* | g607.t1 | 208 | 9.75 | 23959 | Nucleus | 14 | 5 | 7 | 26 |
| *IbbHLH3* | g633.t1 | 293 | 7.12 | 32644.05 | Nucleus | 21 | 5 | 12 | 38 |
| *IbbHLH4* | g934.t1 | 328 | 5.28 | 34994.43 | Nucleus | 38 | 2 | 12 | 52 |
| *IbbHLH5* | g1002.t1 | 254 | 5.18 | 28680.12 | Nucleus | 20 | 0 | 8 | 28 |
| *IbbHLH6* | g1003.t1 | 249 | 7.02 | 27988.64 | Nucleus | 18 | 4 | 6 | 28 |
| *IbbHLH7* | g1005.t1 | 311 | 5.93 | 33799.15 | Nucleus | 30 | 2 | 8 | 40 |
| *IbbHLH8* | g1623.t1 | 274 | 6.55 | 30510.8 | Nucleus | 23 | 2 | 4 | 29 |
| *IbbHLH9* | g2468.t1 | 289 | 5.41 | 32279.15 | Nucleus | 15 | 2 | 9 | 26 |
| *IbbHLH10* | g2470.t1 | 258 | 5.17 | 28936.56 | Nucleus | 13 | 2 | 8 | 23 |
| *IbbHLH11* | g3364.t1 | 135 | 11.52 | 15833.38 | Nucleus | 11 | 0 | 3 | 14 |
| *IbbHLH12* | g4237.t1 | 398 | 5.86 | 43235.23 | Nucleus | 46 | 5 | 10 | 61 |
| *IbbHLH13* | g4338.t1 | 414 | 5.61 | 45126.3 | Nucleus | 47 | 5 | 10 | 62 |
| *IbbHLH14* | g4521.t1 | 780 | 6.61 | 86040.14 | Nucleus | 60 | 7 | 23 | 90 |
| *IbbHLH15* | g5137.t1 | 113 | 6.11 | 12274.84 | Cell membrane Mitochondrion | 3 | 1 | 4 | 8 |
| *IbbHLH16* | g5235.t1 | 454 | 5.29 | 48728.98 | Nucleus | 28 | 3 | 16 | 47 |
| *IbbHLH17* | g5306.t1 | 765 | 5.64 | 84134.67 | Nucleus | 71 | 8 | 21 | 100 |
| *IbbHLH18* | g5327.t1 | 532 | 6.55 | 58460 | Nucleus | 34 | 5 | 4 | 43 |
| *IbbHLH19* | g5347.t1 | 363 | 5.74 | 39638.25 | Nucleus | 18 | 6 | 10 | 34 |
| *IbbHLH20* | g5845.t1 | 392 | 8.87 | 41798.89 | Nucleus | 35 | 2 | 11 | 48 |
| *IbbHLH21* | g6179.t1 | 363 | 6.6 | 40581.56 | Nucleus | 30 | 3 | 8 | 41 |
| *IbbHLH22* | g6183.t1 | 363 | 6.6 | 40477.42 | Nucleus | 31 | 3 | 8 | 42 |
| *IbbHLH23* | g7739.t1 | 228 | 7.78 | 25327.25 | Nucleus | 16 | 4 | 6 | 26 |
| *IbbHLH24* | g7791.t1 | 440 | 6.85 | 48281.37 | Nucleus | 37 | 3 | 9 | 49 |
| *IbbHLH25* | g8057.t1 | 163 | 9.51 | 18336.24 | Chloroplast  Nucleus | 11 | 1 | 5 | 17 |
| *IbbHLH26* | g8058.t1 | 260 | 8.73 | 29775.9 | Nucleus | 19 | 2 | 8 | 29 |
| *IbbHLH27* | g8061.t1 | 166 | 9.82 | 18862.8 | Nucleus | 12 | 2 | 3 | 17 |
| *IbbHLH28* | g8108.t1 | 141 | 5.64 | 15563.93 | Nucleus | 9 | 1 | 1 | 11 |
| *IbbHLH29* | g8111.t1 | 142 | 7.78 | 16068.64 | Nucleus | 9 | 1 | 2 | 12 |
| *IbbHLH30* | g8120.t1 | 162 | 9.22 | 18040.07 | Nucleus | 7 | 1 | 5 | 13 |
| *IbbHLH31* | g8427.t1 | 301 | 5.31 | 32415.47 | Nucleus | 20 | 2 | 8 | 30 |
| *IbbHLH32* | g9041.t1 | 489 | 5.46 | 54214.23 | Nucleus | 29 | 7 | 10 | 46 |
| *IbbHLH33* | g9143.t1 | 464 | 6.55 | 49170.08 | Nucleus | 39 | 5 | 14 | 58 |
| *IbbHLH34* | g9423.t1 | 863 | 6.64 | 94604.16 | Nucleus | 71 | 5 | 22 | 98 |
| *IbbHLH35* | g9534.t1 | 313 | 4.88 | 35598.11 | Nucleus | 24 | 3 | 11 | 38 |
| *IbbHLH36* | g9535.t1 | 330 | 9.04 | 36407.63 | Nucleus | 20 | 2 | 12 | 34 |
| *IbbHLH37* | g9621.t1 | 439 | 7.59 | 48626.06 | Nucleus | 23 | 5 | 3 | 31 |
| *IbbHLH38* | g9624.t1 | 491 | 7.59 | 54308.54 | Nucleus | 28 | 3 | 3 | 34 |
| *IbbHLH39* | g9812.t1 | 422 | 6.33 | 45931.11 | Nucleus | 26 | 2 | 9 | 37 |
| *IbbHLH40* | g9834.t1 | 204 | 5.81 | 22404.67 | Nucleus | 24 | 7 | 2 | 33 |
| *IbbHLH41* | g11240.t1 | 1161 | 6.39 | 128668.66 | Chloroplast | 95 | 9 | 33 | 137 |
| *IbbHLH42* | g11247.t1 | 996 | 5.67 | 108901.52 | Nucleus | 85 | 5 | 21 | 111 |
| *IbbHLH43* | g11502.t1 | 346 | 9.77 | 37536.65 | Nucleus | 30 | 1 | 8 | 39 |
| *IbbHLH44* | g12083.t1 | 125 | 6.3 | 14230.55 | Nucleus | 6 | 1 | 2 | 9 |
| *IbbHLH45* | g12088.t1 | 141 | 8.89 | 15934.53 | Chloroplast | 10 | 3 | 2 | 15 |
| *IbbHLH46* | g12089.t1 | 164 | 9.4 | 18742.52 | Chloroplast  Nucleus | 6 | 1 | 2 | 9 |
| *IbbHLH47* | g12090.t1 | 141 | 7.74 | 15816.35 | Nucleus | 7 | 2 | 1 | 10 |
| *IbbHLH48* | g12093.t1 | 166 | 8.93 | 18678.81 | Nucleus | 10 | 2 | 3 | 15 |
| *IbbHLH49* | g12096.t1 | 145 | 8.73 | 16317.96 | Nucleus | 10 | 2 | 2 | 14 |
| *IbbHLH50* | g12097.t1 | 162 | 9.11 | 18333.46 | Nucleus | 9 | 4 | 2 | 15 |
| *IbbHLH51* | g12144.t1 | 261 | 6.45 | 28435.8 | Nucleus | 17 | 1 | 6 | 24 |
| *IbbHLH52* | g12272.t1 | 492 | 8.1 | 53885.7 | Nucleus | 31 | 3 | 10 | 44 |
| *IbbHLH53* | g12277.t1 | 403 | 6.32 | 43883.66 | Nucleus | 32 | 2 | 14 | 48 |
| *IbbHLH54* | g12372.t1 | 287 | 7.7 | 31341.35 | Nucleus | 20 | 1 | 13 | 34 |
| *IbbHLH55* | g12479.t1 | 236 | 5.24 | 26290.21 | Nucleus | 15 | 0 | 4 | 19 |
| *IbbHLH56* | g12588.t1 | 262 | 6.13 | 28508.81 | Nucleus | 17 | 1 | 6 | 24 |
| *IbbHLH57* | g12826.t1 | 326 | 6.42 | 35981.54 | Nucleus | 22 | 1 | 13 | 36 |
| *IbbHLH58* | g12894.t1 | 479 | 6.2 | 51736.94 | Nucleus | 30 | 2 | 14 | 46 |
| *IbbHLH59* | g13135.t1 | 227 | 5.09 | 25887.17 | Nucleus | 14 | 2 | 6 | 22 |
| *IbbHLH60* | g13167.t1 | 243 | 8.56 | 27609.56 | Nucleus | 13 | 2 | 3 | 18 |
| *IbbHLH61* | g13501.t1 | 216 | 11.82 | 23469.31 | Nucleus | 21 | 1 | 5 | 27 |
| *IbbHLH62* | g13650.t1 | 485 | 5.21 | 52522.88 | Nucleus | 38 | 5 | 5 | 48 |
| *IbbHLH63* | g14524.t1 | 240 | 4.95 | 27220.67 | Nucleus | 26 | 2 | 6 | 34 |
| *IbbHLH64* | g14702.t1 | 247 | 9.05 | 27545.64 | Nucleus | 14 | 2 | 7 | 23 |
| *IbbHLH65* | g14749.t1 | 578 | 5.44 | 62264.88 | Nucleus | 57 | 3 | 12 | 72 |
| *IbbHLH66* | g15746.t1 | 296 | 8.96 | 33499.34 | Nucleus | 13 | 2 | 5 | 20 |
| *IbbHLH67* | g16068.t1 | 435 | 8.46 | 48491.66 | Chloroplast  Nucleus | 32 | 8 | 11 | 51 |
| *IbbHLH68* | g16071.t1 | 548 | 5.13 | 60322.58 | Nucleus | 36 | 7 | 14 | 57 |
| *IbbHLH69* | g16106.t1 | 630 | 5.29 | 69498.87 | Nucleus | 46 | 7 | 12 | 65 |
| *IbbHLH70* | g16560.t1 | 172 | 10.47 | 19381.91 | Chloroplast  Nucleus | 14 | 2 | 8 | 24 |
| *IbbHLH71* | g16681.t1 | 232 | 4.73 | 26383.89 | Nucleus | 18 | 2 | 5 | 25 |
| *IbbHLH72* | g16793.t1 | 259 | 8.05 | 28118.19 | Nucleus | 17 | 2 | 10 | 29 |
| *IbbHLH73* | g17124.t1 | 286 | 8.08 | 32283.88 | Nucleus | 17 | 3 | 9 | 29 |
| *IbbHLH74* | g17144.t1 | 250 | 8.12 | 28409.66 | Nucleus | 9 | 1 | 8 | 18 |
| *IbbHLH75* | g17216.t1 | 370 | 5.38 | 40204.54 | Nucleus | 35 | 5 | 7 | 47 |
| *IbbHLH76* | g17220.t1 | 232 | 9.24 | 25479.36 | Nucleus | 20 | 3 | 4 | 27 |
| *IbbHLH77* | g17367.t1 | 110 | 8.89 | 12102.52 | Nucleus | 14 | 1 | 4 | 19 |
| *IbbHLH78* | g17792.t1 | 135 | 11.52 | 15833.38 | Nucleus | 11 | 0 | 3 | 14 |
| *IbbHLH79* | g18028.t1 | 214 | 5.89 | 23449.79 | Nucleus | 26 | 9 | 3 | 38 |
| *IbbHLH80* | g19306.t1 | 470 | 6.15 | 52832.51 | Nucleus | 40 | 4 | 15 | 59 |
| *IbbHLH81* | g19319.t1 | 602 | 5.22 | 66524.45 | Nucleus | 62 | 5 | 15 | 82 |
| *IbbHLH82* | g19329.t1 | 605 | 5.07 | 66742.47 | Nucleus | 60 | 5 | 15 | 80 |
| *IbbHLH83* | g19429.t1 | 608 | 5.26 | 67541.67 | Nucleus | 59 | 4 | 14 | 77 |
| *IbbHLH84* | g19768.t1 | 422 | 8.29 | 46369.05 | Nucleus | 42 | 4 | 13 | 59 |
| *IbbHLH85* | g20532.t1 | 278 | 9.03 | 30898.74 | Nucleus | 21 | 2 | 8 | 31 |
| *IbbHLH86* | g20637.t1 | 303 | 9.14 | 33357.12 | Nucleus | 34 | 1 | 9 | 44 |
| *IbbHLH87* | g20804.t1 | 192 | 6.16 | 21370.08 | Nucleus | 4 | 1 | 5 | 10 |
| *IbbHLH88* | g20973.t1 | 381 | 10.13 | 43153.5 | Nucleus | 38 | 2 | 10 | 50 |
| *IbbHLH89* | g20979.t1 | 164 | 8.83 | 18598.42 | Nucleus | 14 | 3 | 5 | 22 |
| *IbbHLH90* | g20982.t1 | 153 | 8.9 | 17409.19 | Nucleus | 12 | 5 | 1 | 18 |
| *IbbHLH91* | g20985.t1 | 153 | 9.05 | 17532.39 | Nucleus | 10 | 4 | 1 | 15 |
| *IbbHLH92* | g20992.t1 | 146 | 8.73 | 16475.09 | Nucleus | 10 | 3 | 2 | 15 |
| *IbbHLH93* | g22192.t1 | 165 | 9.46 | 18752.55 | Chloroplast | 6 | 2 | 2 | 10 |
| *IbbHLH94* | g22197.t1 | 142 | 8.53 | 16067.66 | Nucleus | 8 | 1 | 2 | 11 |
| *IbbHLH95* | g22200.t1 | 167 | 9.28 | 18964.97 | Nucleus | 8 | 2 | 2 | 12 |
| *IbbHLH96* | g22203.t1 | 155 | 7.86 | 17639.23 | Nucleus | 9 | 1 | 2 | 12 |
| *IbbHLH97* | g23292.t1 | 181 | 10.66 | 19982.87 | Nucleus | 11 | 2 | 8 | 21 |
| *IbbHLH98* | g23731.t1 | 336 | 5.52 | 37221.65 | Nucleus | 26 | 6 | 13 | 45 |
| *IbbHLH99* | g24487.t1 | 169 | 5.55 | 17923.9 | Nucleus | 14 | 3 | 2 | 19 |
| *IbbHLH100* | g24748.t1 | 183 | 8.35 | 20943.89 | Nucleus | 14 | 1 | 4 | 19 |
| *IbbHLH101* | g24949.t1 | 922 | 6.47 | 102435.42 | Nucleus | 55 | 9 | 28 | 92 |
| *IbbHLH102* | g24955.t1 | 356 | 5.91 | 38067.49 | Nucleus | 36 | 3 | 6 | 45 |
| *IbbHLH103* | g25336.t1 | 294 | 6 | 33179.78 | Nucleus | 19 | 4 | 8 | 31 |
| *IbbHLH104* | g25337.t1 | 255 | 8.61 | 28710.53 | Nucleus | 21 | 4 | 9 | 34 |
| *IbbHLH105* | g25466.t1 | 137 | 6.83 | 15153.29 | Nucleus | 16 | 1 | 5 | 22 |
| *IbbHLH106* | g25500.t1 | 254 | 6.39 | 27831.3 | Nucleus | 23 | 2 | 8 | 33 |
| *IbbHLH107* | g25642.t1 | 257 | 7.55 | 27871.6 | Nucleus | 16 | 3 | 5 | 24 |
| *IbbHLH108* | g25833.t1 | 512 | 7.71 | 56877 | Nucleus | 42 | 3 | 9 | 54 |
| *IbbHLH109* | g25891.t1 | 472 | 9.03 | 51830.31 | Nucleus | 39 | 2 | 11 | 52 |
| *IbbHLH110* | g25978.t1 | 405 | 6.72 | 44423.82 | Nucleus | 26 | 2 | 8 | 36 |
| *IbbHLH111* | g26525.t1 | 464 | 5.99 | 48455.64 | Nucleus | 41 | 1 | 14 | 56 |
| *IbbHLH112* | g27371.t1 | 283 | 6.01 | 30165.4 | Nucleus | 23 | 1 | 6 | 30 |
| *IbbHLH113* | g29114.t1 | 431 | 5.55 | 46727.25 | Nucleus | 44 | 3 | 9 | 56 |
| *IbbHLH114* | g29116.t1 | 271 | 6.47 | 30863.63 | Nucleus | 17 | 2 | 10 | 29 |
| *IbbHLH115* | g29126.t1 | 321 | 5.59 | 35864.43 | Nucleus | 18 | 1 | 11 | 30 |
| *IbbHLH116* | g29158.t1 | 327 | 6.4 | 35178.57 | Nucleus | 40 | 7 | 12 | 59 |
| *IbbHLH117* | g29332.t1 | 397 | 8.64 | 43205.78 | Nucleus | 33 | 3 | 12 | 48 |
| *IbbHLH118* | g29350.t1 | 341 | 8.5 | 37248.52 | Nucleus | 31 | 8 | 4 | 43 |
| *IbbHLH119* | g29854.t1 | 319 | 5.95 | 35790.23 | Nucleus | 20 | 8 | 7 | 35 |
| *IbbHLH120* | g30012.t1 | 297 | 4.68 | 33428.88 | Nucleus | 29 | 4 | 12 | 45 |
| *IbbHLH121* | g30090.t1 | 240 | 6.08 | 27055.17 | Nucleus | 19 | 4 | 8 | 31 |
| *IbbHLH122* | g31392.t1 | 255 | 7.24 | 27835.77 | Nucleus | 40 | 2 | 5 | 47 |
| *IbbHLH123* | g33866.t1 | 264 | 6.92 | 28474.77 | Nucleus | 20 | 3 | 5 | 28 |
| *IbbHLH124* | g33888.t1 | 438 | 6.62 | 48368.68 | Nucleus | 28 | 3 | 13 | 44 |
| *IbbHLH125* | g34207.t1 | 343 | 6.28 | 38464.54 | Nucleus | 31 | 2 | 6 | 39 |
| *IbbHLH126* | g34256.t1 | 362 | 4.87 | 39816.14 | Nucleus | 33 | 2 | 8 | 43 |
| *IbbHLH127* | g34447.t1 | 236 | 5.9 | 26201.96 | Nucleus | 12 | 2 | 2 | 16 |
| *IbbHLH128* | g34604.t1 | 158 | 7.65 | 17730.25 | Nucleus | 13 | 2 | 2 | 17 |
| *IbbHLH129* | g35045.t1 | 743 | 4.94 | 82065.92 | Nucleus | 69 | 8 | 19 | 96 |
| *IbbHLH130* | g35355.t1 | 547 | 5.62 | 58489.63 | Nucleus | 42 | 2 | 7 | 51 |
| *IbbHLH131* | g35474.t1 | 694 | 5.98 | 74039.14 | Nucleus | 58 | 1 | 16 | 75 |
| *IbbHLH132* | g37849.t1 | 360 | 8.86 | 40443.31 | Nucleus | 13 | 9 | 8 | 30 |
| *IbbHLH133* | g37863.t1 | 366 | 8.96 | 41261.25 | Nucleus | 16 | 6 | 9 | 31 |
| *IbbHLH134* | g38205.t1 | 244 | 9.61 | 27051.79 | Nucleus | 14 | 0 | 4 | 18 |
| *IbbHLH135* | g38353.t1 | 662 | 5.8 | 71853.25 | Nucleus | 50 | 4 | 14 | 68 |
| *IbbHLH136* | g38365.t1 | 318 | 6.4 | 34240.81 | Nucleus | 10 | 1 | 10 | 21 |
| *IbbHLH137* | g38692.t1 | 231 | 6.92 | 25166.55 | Nucleus | 19 | 4 | 7 | 30 |
| *IbbHLH138* | g38747.t1 | 345 | 4.82 | 38447.64 | Nucleus | 32 | 3 | 4 | 39 |
| *IbbHLH139* | g38780.t1 | 174 | 7.76 | 20011.69 | Nucleus | 11 | 2 | 1 | 14 |
| *IbbHLH140* | g38960.t1 | 376 | 5.29 | 41576.31 | Nucleus | 22 | 5 | 9 | 36 |
| *IbbHLH141* | g39242.t1 | 343 | 5.96 | 38046.92 | Nucleus | 24 | 4 | 11 | 39 |
| *IbbHLH142* | g40985.t1 | 321 | 5.4 | 36275.57 | Nucleus | 17 | 11 | 1 | 29 |
| *IbbHLH143* | g41166.t1 | 285 | 5.65 | 30312.55 | Nucleus | 23 | 1 | 6 | 30 |
| *IbbHLH144* | g41640.t1 | 207 | 11.82 | 22810.51 | Nucleus | 18 | 1 | 3 | 22 |
| *IbbHLH145* | g41643.t1 | 210 | 11.82 | 23055.74 | Nucleus | 18 | 1 | 3 | 22 |
| *IbbHLH146* | g41758.t1 | 423 | 8.27 | 44596.45 | Nucleus | 39 | 1 | 5 | 45 |
| *IbbHLH147* | g41898.t1 | 1081 | 5.79 | 116892.72 | Nucleus | 118 | 8 | 22 | 148 |
| *IbbHLH148* | g41914.t1 | 448 | 6.88 | 49856.05 | Chloroplast | 27 | 5 | 12 | 44 |
| *IbbHLH149* | g41945.t1 | 312 | 5.05 | 35319.1 | Nucleus | 27 | 3 | 4 | 34 |
| *IbbHLH150* | g41970.t1 | 391 | 6.02 | 42616.55 | Nucleus | 32 | 1 | 6 | 39 |
| *IbbHLH151* | g42002.t1 | 370 | 5.03 | 41620.82 | Nucleus | 23 | 6 | 7 | 36 |
| *IbbHLH152* | g42034.t1 | 638 | 8.24 | 71328.47 | Nucleus | 29 | 4 | 27 | 60 |
| *IbbHLH153* | g42044.t1 | 381 | 4.89 | 41613.36 | Nucleus | 31 | 3 | 11 | 45 |
| *IbbHLH154* | g42098.t1 | 339 | 5.66 | 36891.44 | Nucleus | 15 | 4 | 10 | 29 |
| *IbbHLH155* | g42688.t1 | 523 | 5.92 | 56556.32 | Nucleus | 41 | 2 | 13 | 56 |
| *IbbHLH156* | g42835.t1 | 482 | 5.22 | 53949.24 | Chloroplast  Nucleus | 27 | 2 | 6 | 35 |
| *IbbHLH157* | g42953.t1 | 383 | 9.32 | 42583.17 | Nucleus | 30 | 3 | 9 | 42 |
| *IbbHLH158* | g43097.t1 | 292 | 9.11 | 33342.21 | Nucleus | 18 | 2 | 7 | 27 |
| *IbbHLH159* | g43248.t1 | 297 | 5.77 | 33432.66 | Nucleus | 19 | 5 | 5 | 29 |
| *IbbHLH160* | g43717.t1 | 258 | 8.9 | 29195.51 | Nucleus | 14 | 1 | 7 | 22 |
| *IbbHLH161* | g44153.t1 | 698 | 5.72 | 74439.24 | Nucleus | 70 | 2 | 17 | 89 |
| *IbbHLH162* | g44626.t1 | 192 | 9.12 | 21758.15 | Nucleus | 18 | 1 | 4 | 23 |
| *IbbHLH163* | g44627.t1 | 192 | 8.73 | 21788.17 | Nucleus | 16 | 1 | 3 | 20 |
| *IbbHLH164* | g44635.t1 | 192 | 8.73 | 21788.17 | Nucleus | 16 | 1 | 3 | 20 |
| *IbbHLH165* | g44636.t1 | 159 | 8.62 | 18352.27 | Nucleus | 15 | 1 | 3 | 19 |
| *IbbHLH166* | g45156.t1 | 229 | 5.18 | 26158.44 | Nucleus | 16 | 2 | 5 | 23 |
| *IbbHLH167* | g46180.t1 | 192 | 9.07 | 21208.88 | Nucleus | 22 | 0 | 0 | 22 |
| *IbbHLH168* | g46287.t1 | 91 | 6.16 | 9780.1 | Nucleus | 10 | 0 | 0 | 10 |
| *IbbHLH169* | g46389.t1 | 357 | 5.1 | 39599.22 | Nucleus | 30 | 3 | 15 | 48 |
| *IbbHLH170* | g46392.t1 | 221 | 5.78 | 24764.3 | Nucleus | 12 | 0 | 5 | 17 |
| *IbbHLH171* | g46399.t1 | 237 | 6.01 | 26206.78 | Nucleus | 15 | 0 | 2 | 17 |
| *IbbHLH172* | g46801.t1 | 310 | 7.07 | 34473.44 | Nucleus | 18 | 3 | 11 | 32 |
| *IbbHLH173* | g46840.t1 | 392 | 6.79 | 44310.54 | Nucleus | 24 | 2 | 12 | 38 |
| *IbbHLH174* | g46841.t1 | 236 | 7.14 | 26627.99 | Nucleus | 25 | 3 | 7 | 35 |
| *IbbHLH175* | g47048.t1 | 275 | 5.22 | 31336.11 | Nucleus | 14 | 3 | 6 | 23 |
| *IbbHLH176* | g47100.t1 | 476 | 5.86 | 49484.01 | Nucleus | 34 | 1 | 15 | 50 |
| *IbbHLH177* | g47190.t1 | 318 | 7.11 | 33828.4 | Nucleus | 25 | 3 | 4 | 32 |
| *IbbHLH178* | g47436.t1 | 496 | 5.96 | 52522.58 | Nucleus | 35 | 1 | 14 | 50 |
| *IbbHLH179* | g47777.t1 | 215 | 4.68 | 23851.72 | Cytoplasm | 16 | 3 | 6 | 25 |
| *IbbHLH180* | g48979.t1 | 493 | 6.38 | 53107.62 | Nucleus | 46 | 3 | 12 | 61 |
| *IbbHLH181* | g49455.t1 | 544 | 5.79 | 59534.49 | Nucleus | 31 | 3 | 22 | 56 |
| *IbbHLH182* | g49961.t1 | 157 | 6.51 | 17219.59 | Nucleus | 14 | 2 | 5 | 21 |
| *IbbHLH183* | g50409.t1 | 488 | 5.59 | 54434.71 | Nucleus | 32 | 5 | 10 | 47 |
| *IbbHLH184* | g50983.t1 | 508 | 6.17 | 56617.24 | Nucleus | 45 | 3 | 10 | 58 |
| *IbbHLH185* | g52626.t1 | 507 | 5.92 | 55717.52 | Nucleus | 31 | 2 | 6 | 39 |
| *IbbHLH186* | g52855.t1 | 295 | 7.74 | 33471.71 | Nucleus | 19 | 2 | 12 | 33 |
| *IbbHLH187* | g53432.t1 | 221 | 5.57 | 24090.61 | Nucleus | 13 | 2 | 5 | 20 |
| *IbbHLH188* | g54081.t1 | 538 | 9.04 | 59501.43 | Nucleus | 33 | 2 | 18 | 53 |
| *IbbHLH189* | g54106.t1 | 491 | 6.4 | 52589.58 | Nucleus | 55 | 5 | 19 | 79 |
| *IbbHLH190* | g54230.t1 | 371 | 5.83 | 41468.84 | Nucleus | 23 | 1 | 7 | 31 |
| *IbbHLH191* | g54232.t1 | 402 | 5.53 | 45040.51 | Nucleus | 23 | 1 | 10 | 34 |
| *IbbHLH192* | g54594.t1 | 254 | 8.41 | 28110.7 | Nucleus | 30 | 2 | 9 | 41 |
| *IbbHLH193* | g54673.t1 | 240 | 5.83 | 26750.57 | Nucleus | 13 | 1 | 6 | 20 |
| *IbbHLH194* | g54841.t1 | 886 | 6.58 | 97031.86 | Nucleus | 60 | 4 | 21 | 85 |
| *IbbHLH195* | g55288.t1 | 337 | 6.64 | 36214.67 | Nucleus | 38 | 4 | 10 | 52 |
| *IbbHLH196* | g55614.t1 | 581 | 6.17 | 64892.88 | Nucleus | 40 | 5 | 22 | 67 |
| *IbbHLH197* | g55758.t1 | 456 | 8.84 | 49687.12 | Nucleus | 20 | 3 | 13 | 36 |
| *IbbHLH198* | g55778.t1 | 458 | 9.41 | 49946.53 | Nucleus | 22 | 2 | 13 | 37 |
| *IbbHLH199* | g55794.t1 | 517 | 7.13 | 56187 | Nucleus | 24 | 4 | 16 | 44 |
| *IbbHLH200* | g57106.t1 | 352 | 6.33 | 39230.14 | Nucleus | 34 | 3 | 9 | 46 |
| *IbbHLH201* | g57259.t1 | 360 | 5.32 | 40553.41 | Nucleus | 26 | 5 | 11 | 42 |
| *IbbHLH202* | g57590.t1 | 396 | 6.42 | 43635.35 | Nucleus | 42 | 4 | 7 | 53 |
| *IbbHLH203* | g58484.t1 | 381 | 6.78 | 42730.33 | Nucleus | 20 | 2 | 10 | 32 |
| *IbbHLH204* | g58568.t1 | 240 | 6.62 | 27205.74 | Nucleus | 24 | 0 | 5 | 29 |
| *IbbHLH205* | g58925.t1 | 795 | 5.03 | 88120.22 | Nucleus | 66 | 10 | 19 | 95 |
| *IbbHLH206* | g59260.t1 | 423 | 6.64 | 46864.83 | Nucleus | 43 | 2 | 10 | 55 |
| *IbbHLH207* | g59522.t1 | 454 | 5.71 | 49678.25 | Nucleus | 40 | 8 | 11 | 59 |
| *IbbHLH208* | g59640.t1 | 256 | 7.64 | 26980.71 | Nucleus | 16 | 1 | 9 | 26 |
| *IbbHLH209* | g59672.t1 | 300 | 6.55 | 32766.85 | Nucleus | 16 | 1 | 5 | 22 |
| *IbbHLH210* | g59748.t1 | 324 | 9.29 | 35940.98 | Nucleus | 21 | 2 | 12 | 35 |
| *IbbHLH211* | g59794.t1 | 313 | 5.89 | 35534.62 | Nucleus | 29 | 2 | 11 | 42 |
| *IbbHLH212* | g59915.t1 | 195 | 6.47 | 22108.58 | Mitochondrion  Nucleus | 11 | 1 | 9 | 21 |
| *IbbHLH213* | g59965.t1 | 228 | 4.92 | 25341.44 | Nucleus | 12 | 3 | 7 | 22 |
| *IbbHLH214* | g60027.t1 | 995 | 8.91 | 111241.41 | Nucleus | 65 | 5 | 32 | 102 |
| *IbbHLH215* | g60226.t1 | 367 | 4.8 | 40733.89 | Nucleus | 28 | 3 | 11 | 42 |
| *IbbHLH216* | g60256.t1 | 316 | 4.74 | 35436.99 | Nucleus | 21 | 3 | 4 | 28 |
| *IbbHLH217* | g60295.t1 | 361 | 6.47 | 40723.68 | Nucleus | 25 | 2 | 5 | 32 |
| *IbbHLH218* | g60336.t1 | 251 | 4.88 | 28107.79 | Nucleus | 22 | 1 | 5 | 28 |
| *IbbHLH219* | g60357.t1 | 279 | 5.39 | 31065.24 | Nucleus | 20 | 3 | 13 | 36 |
| *IbbHLH220* | g60396.t1 | 377 | 9.08 | 41512.5 | Nucleus | 28 | 2 | 14 | 44 |
| *IbbHLH221* | g60403.t1 | 239 | 9.18 | 26530.29 | Nucleus | 23 | 4 | 6 | 33 |
| *IbbHLH222* | g60618.t1 | 315 | 5.37 | 34979.51 | Nucleus | 13 | 3 | 5 | 21 |
| *IbbHLH223* | g61009.t1 | 395 | 5.42 | 42893.75 | Nucleus | 32 | 3 | 5 | 40 |
| *IbbHLH224* | g61110.t1 | 375 | 6.6 | 41089.47 | Nucleus | 38 | 3 | 12 | 53 |
| *IbbHLH225* | g61504.t1 | 272 | 5.92 | 30472.33 | Nucleus | 19 | 8 | 8 | 35 |
| *IbbHLH226* | g62765.t1 | 263 | 5.68 | 28971.78 | Nucleus | 19 | 4 | 11 | 34 |
| *IbbHLH227* | g62835.t1 | 627 | 5.3 | 69117.23 | Nucleus | 65 | 5 | 14 | 84 |
