# Supplementary material for: Systematic identification and expression analysis of bHLH gene family reveal their relevance to abiotic stress response and anthocyanin biosynthesis in sweetpotato
Source: BMC Plant Biol. 2024 Mar 1;24:156. doi: 10.1186/s12870-024-04788-0 (PMC10905920; doi:10.1186/s12870-024-04788-0)
Supplement: Supplementary file 3 — Supplementary Material 3 [file 12870_2024_4788_MOESM3_ESM.docx]

**Additional file 3**. The 162 AtbHLH sequences from Arabidopsis and 166 OsbHLH sequences from rice.

>AtbHLH001

MATGQNRTTVPENLKKHLAVSVRNIQWSYGIFWSVSASQSGVLEWGDGYYNGDIKTRKTIQASEIKADQLGLRRSEQLSELYESLSVAESSSSGVAAGSQVTRRASAAALSPEDLADTEWYYLVCMSFVFNIGEGMPGRTFANGEPIWLCNAHTADSKVFSRSLLAKSAAVKTVVCFPFLGGVVEIGTTEHITEDMNVIQCVKTSFLEAPDPYATILPARSDYHIDNVLDPQQILGDEIYAPMFSTEPFPTASPSRTTNGFDQEHEQVADDHDSFMTERITGGASQVQSWQLMDDELSNCVHQSLNSSDCVSQTFVEGAAGRVAYGARKSRVQRLGQIQEQQRNVKTLSFDPRNDDVHYQSVISTIFKTNHQLILGPQFRNCDKQSSFTRWKKSSSSSSGTATVTAPSQGMLKKIIFDVPRVHQKEKLMLDSPEARDETGNHAVLEKKRREKLNERFMTLRKIIPSINKIDKVSILDDTIEYLQELERRVQELESCRESTDTETRGTMTMKRKKPCDAGERTSANCANNETGNGKKVSVNNVGEAEPADTGFTGLTDNLRIGSFGNEVVIELRCAWREGVLLEIMDVISDLHLDSHSVQSSTGDGLLCLTVNCKHKGSKIATPGMIKEALQRVAWIC

>AtbHLH002

MATGENRTVPDNLKKQLAVSVRNIQWSYGIFWSVSASQPGVLEWGDGYYNGDIKTRKTIQAAEVKIDQLGLERSEQLRELYESLSLAESSASGSSQVTRRASAAALSPEDLTDTEWYYLVCMSFVFNIGEGIPGGALSNGEPIWLCNAETADSKVFTRSLLAKSASLQTVVCFPFLGGVLEIGTTEHIKEDMNVIQSVKTLFLEAPPYTTISTRSDYQEIFDPLSDDKYTPVFITEAFPTTSTSGFEQEPEDHDSFINDGGASQVQSWQFVGEEISNCIHQSLNSSDCVSQTFVGTTGRLACDPRKSRIQRLGQIQEQSNHVNMDDDVHYQGVISTIFKTTHQLILGPQFQNFDKRSSFTRWKRSSSVKTLGEKSQKMIKKILFEVPLMNKKEELLPDTPEETGNHALSEKKRREKLNERFMTLRSIIPSISKIDKVSILDDTIEYLQDLQKRVQELESCRESADTETRITMMKRKKPDDEEERASANCMNSKRKGSDVNVGEDEPADIGYAGLTDNLRISSLGNEVVIELRCAWREGILLEIMDVISDLNLDSHSVQSSTGDGLLCLTVNCKHKGTKIATTGMIQEALQRVAWIC

>AtbHLH003

MGQKFWENQEDRAMVESTIGSEACDFFISTASASNTALSKLVSPPSDSNLQQGLRHVVEGSDWDYALFWLASNVNSSDGCVLIWGDGHCRVKKGASGEDYSQQDEIKRRVLRKLHLSFVGSDEDHRLVKSGALTDLDMFYLASLYFSFRCDTNKYGPAGTYVSGKPLWAADLPSCLSYYRVRSFLARSAGFQTVLSVPVNSGVVELGSLRHIPEDKSVIEMVKSVFGGSDFVQAKEAPKIFGRQLSLGGAKPRSMSINFSPKTEDDTGFSLESYEVQAIGGSNQVYGYEQGKDETLYLTDEQKPRKRGRKPANGREEALNHVEAERQRREKLNQRFYALRAVVPNISKMDKASLLADAITYITDMQKKIRVYETEKQIMKRRESNQITPAEVDYQQRHDDAVVRLSCPLETHPVSKVIQTLRENEVMPHDSNVAITEEGVVHTFTLRPQGGCTAEQLKDKLLASLSQ

>AtbHLH004

MSPTNVQVTDYHLNQSKTDTTNLWSTDDDASVMEAFIGGGSDHSSLFPPLPPPPLPQVNEDNLQQRLQALIEGANENWTYAVFWQSSHGFAGEDNNNNNTVLLGWGDGYYKGEEEKSRKKKSNPASAAEQEHRKRVIRELNSLISGGVGGGDEAGDEEVTDTEWFFLVSMTQSFVKGTGLPGQAFSNSDTIWLSGSNALAGSSCERARQGQIYGLQTMVCVATENGVVELGSSEIIHQSSDLVDKVDTFFNFNNGGGEFGSWAFNLNPDQGENDPGLWISEPNGVDSGLVAAPVMNNGGNDSTSNSDSQPISKLCNGSSVENPNPKVLKSCEMVNFKNGIENGQEEDSSNKKRSPVSNNEEGMLSFTSVLPCDSNHSDLEASVAKEAESNRVVVEPEKKPRKRGRKPANGREEPLNHVEAERQRREKLNQRFYSLRAVVPNVSKMDKASLLGDAISYISELKSKLQKAESDKEELQKQIDVMNKEAGNAKSSVKDRKCLNQESSVLIEMEVDVKIIGWDAMIRIQCSKRNHPGAKFMEALKELDLEVNHASLSVVNDLMIQQATVKMGNQFFTQDQLKVALTEKVGECP

>AtbHLH005

MNGTTSSINFLTSDDDASAAAMEAFIGTNHHSSLFPPPPQQPPQPQFNEDTLQQRLQALIESAGENWTYAIFWQISHDFDSSTGDNTVILGWGDGYYKGEEDKEKKKNNTNTAEQEHRKRVIRELNSLISGGIGVSDESNDEEVTDTEWFFLVSMTQSFVNGVGLPGESFLNSRVIWLSGSGALTGSGCERAGQGQIYGLKTMVCIATQNGVVELGSSEVISQSSDLMHKVNNLFNFNNGGGNNGVEASSWGFNLNPDQGENDPALWISEPTNTGIESPARVNNGNNSNSNSKSDSHQISKLEKNDISSVENQNRQSSCLVEKDLTFQGGLLKSNETLSFCGNESSKKRTSVSKGSNNDEGMLSFSTVVRSAANDSDHSDLEASVVKEAIVVEPPEKKPRKRGRKPANGREEPLNHVEAERQRREKLNQRFYSLRAVVPNVSKMDKASLLGDAISYINELKSKLQQAESDKEEIQKKLDGMSKEGNNGKGCGSRAKERKSSNQDSTASSIEMEIDVKIIGWDVMIRVQCGKKDHPGARFMEALKELDLEVNHASLSVVNDLMIQQATVKMGSQFFNHDQLKVALMTKVGENY

>AtbHLH006

MTDYRLQPTMNLWTTDDNASMMEAFMSSSDISTLWPPASTTTTTATTETTPTPAMEIPAQAGFNQETLQQRLQALIEGTHEGWTYAIFWQPSYDFSGASVLGWGDGYYKGEEDKANPRRRSSSPPFSTPADQEYRKKVLRELNSLISGGVAPSDDAVDEEVTDTEWFFLVSMTQSFACGAGLAGKAFATGNAVWVSGSDQLSGSGCERAKQGGVFGMHTIACIPSANGVVEVGSTEPIRQSSDLINKVRILFNFDGGAGDLSGLNWNLDPDQGENDPSMWINDPIGTPGSNEPGNGAPSSSSQLFSKSIQFENGSSSTITENPNLDPTPSPVHSQTQNPKFNNTFSRELNFSTSSSTLVKPRSGEILNFGDEGKRSSGNPDPSSYSGQTQFENKRKRSMVLNEDKVLSFGDKTAGESDHSDLEASVVKEVAVEKRPKKRGRKPANGREEPLNHVEAERQRREKLNQRFYALRAVVPNVSKMDKASLLGDAIAYINELKSKVVKTESEKLQIKNQLEEVKLELAGRKASASGGDMSSSCSSIKPVGMEIEVKIIGWDAMIRVESSKRNHPAARLMSALMDLELEVNHASMSVVNDLMIQQATVKMGFRIYTQEQLRASLISKIG

>AtbHLH007

MANNNNIPHDSISDPSPTDDFFEQILGLSNFSGSSGSGLSGIGGVGPPPMMLQLGSGNEGNHNHMGAIGGGGPVGFHNQMFPLGLSLDQGKGHGFLKPDETGKRFQDDVLDNRCSSMKPIFHGQPMSQPAPPMPHQQSTIRPRVRARRGQATDPHSIAERLRRERIAERIRSLQELVPTVNKTDRAAMIDEIVDYVKFLRLQVKVLSMSRLGGAGAVAPLVTEMPLSSSVEDETQAVWEKWSNDGTERQVAKLMEENVGAAMQLLQSKALCIMPISLAMAIYHSQPPDTSSSIVKPEMNPPP

>AtbHLH008

MPLFELFRLTKAKLESAQDRNPSPPVDEVVELVWENGQISTQSQSSRSRNIPPPQANSSRAREIGNGSKTTMVDEIPMSVPSLMTGLSQDDDFVPWLNHHPSLDGYCSDFLRDVSSPVTVNEQESDMAVNQTAFPLFQRRKDGNESAPAASSSQYNGFQSHSLYGSDRARDLPSQQTNPDRFTQTQEPLITSNKPSLVNFSHFLRPATFAKTTNNNLHDTKEKSPQSPPNVFQTRVLGAKDSEDKVLNESVASATPKDNQKACLISEDSCRKDQESEKAVVCSSVGSGNSLDGPSESPSLSLKRKHSNIQDIDCHSEDVEEESGDGRKEAGPSRTGLGSKRSRSAEVHNLSERRRRDRINEKMRALQELIPNCNKVDKASMLDEAIEYLKSLQLQVQIMSMASGYYLPPAVMFPPGMGHYPAAAAAMAMGMGMPYAMGLPDLSRGGSSVNHGPQFQVSGMQQQPVAMGIPRVSGGGIFAGSSTIGNGSTRDLSGSKDQTTTNNNSNLKPIKRKQGSSDQFCGSS

>AtbHLH009

MEHQGWSFEENYSLSTNRRSIRPQDELVELLWRDGQVVLQSQTHREQTQTQKQDHHEEALRSSTFLEDQETVSWIQYPPDEDPFEPDDFSSHFFSTMDPLQRPTSETVKPKSSPEPPQVMVKPKACPDPPPQVMPPPKFRLTNSSSGIRETEMEQYSVTTVGPSHCGSNPSQNDLDVSMSHDRSKNIEEKLNPNASSSSGGSSGCSFGKDIKEMASGRCITTDRKRKRINHTDESVSLSDAIGNKSNQRSGSNRRSRAAEVHNLSERRRRDRINERMKALQELIPHCSKTDKASILDEAIDYLKSLQLQLQVMWMGSGMAAAAASAPMMFPGVQPQQFIRQIQSPVQLPRFPVMDQSAIQNNPGLVCQNPVQNQIISDRFARYIGGFPHMQAATQMQPMEMLRFSSPAGQQSQQPSSVPTKTTDGSRLDH

>AtbHLH010

MEEERESLYEEMGCFDPNTPAEVTVESSFSQAEPPPPPPQVLVAGSTSNSNCSVEVEELSEFHLSPQDCPQASSTPLQFHINPPPPPPPPCDQLHNNLIHQMASHQQQHSNWDNGYQDFVNLGPNSATTPDLLSLLHLPRCSLPPNHHPSSMLPTSFSDIMSSSSAAAVMYDPLFHLNFPMQPRDQNQLRNGSCLLGVEDQIQMDANGGMNVLYFEGANNNNGGFENEILEFNNGVTRKGRGSRKSRTSPTERERRVHFNDRFFDLKNLIPNPTKIDRASIVGEAIDYIKELLRTIEEFKMLVEKKRCGRFRSKKRARVGEGGGGEDQEEEEDTVNYKPQSEVDQSCFNKNNNNSLRCSWLKRKSKVTEVDVRIIDDEVTIKLVQKKKINCLLFTTKVLDQLQLDLHHVAGGQIGEHYSFLFNTKICEGSCVYASGIADTLMEVVEKQYMEAVPSNGY

>AtbHLH011

MDQPMKPKTCSESDFADDSSASSSSSSGQNLRGAEMVVEVKKEAVCSQKAEREKLRRDKLKEQFLELGNALDPNRPKSDKASVLTDTIQMLKDVMNQVDRLKAEYETLSQESRELIQEKSELREEKATLKSDIEILNAQYQHRIKTMVPWVPHYSYHIPFVAITQGQSSFIPYSASVNPLTEQQASVQQHSSSSADASMKQDSKIKPLDLDLMMNSNHSGQGNDQKDDVRLKLELKIHASSLAQQDVSGKEKKVSLTTTASSSNSYSLSQAVQDSSPGTVNDMLKP

>AtbHLH012

MSLTMADGVEAAAGRSKRQNSLLRKQLALAVRSVQWSYAIFWSSSLTQPGVLEWGEGCYNGDMKKRKKSYESHYKYGLQKSKELRKLYLSMLEGDSGTTVSTTHDNLNDDDDNCHSTSMMLSPDDLSDEEWYYLVSMSYVFSPSQCLPGRASATGETIWLCNAQYAENKLFSRSLLARSASIQTVVCFPYLGGVIELGVTELISEDHNLLRNIKSCLMEISAHQDNDDEKKMEIKISEEKHQLPLGISDEDLHYKRTISTVLNYSADRSGKNDKNIRHRQPNIVTSEPGSSFLRWKQCEQQVSGFVQKKKSQNVLRKILHDVPLMHTKRMFPSQNSGLNQDDPSDRRKENEKFSVLRTMVPTVNEVDKESILNNTIKYLQELEARVEELESCMGSVNFVERQRKTTENLNDSVLIEETSGNYDDSTKIDDNSGETEQVTVFRDKTHLRVKLKETEVVIEVRCSYRDYIVADIMETLSNLHMDAFSVRSHTLNKFLTLNLKAKFRGAAVASVGMIKRELRRVIGDLF

>AtbHLH013

MNIGRLVWNEDDKAIVASLLGKRALDYLLSNSVSNANLLMTLGSDENLQNKLSDLVERPNASNFSWNYAIFWQISRSKAGDLVLCWGDGYCREPKEGEKSEIVRILSMGREEETHQTMRKRVLQKLHDLFGGSEEENCALGLDRVTDTEMFLLSSMYFSFPRGEGGPGKCFASAKPVWLSDVVNSGSDYCVRSFLAKSAGIQTVVLVPTDLGVVELGSTSCLPESEDSILSIRSLFTSSLPPVRAVALPVTVAEKIDDNRTKIFGKDLHNSGFLQHHQHHQQQQQQPPQQQQHRQFREKLTVRKMDDRAPKRLDAYPNNGNRFMFSNPGTNNNTLLSPTWVQPENYTRPINVKEVPSTDEFKFLPLQQSSQRLLPPAQMQIDFSAASSRASENNSDGEGGGEWADAVGADESGNNRPRKRGRRPANGRAEALNHVEAERQRREKLNQRFYALRSVVPNISKMDKASLLGDAVSYINELHAKLKVMEAERERLGYSSNPPISLDSDINVQTSGEDVTVRINCPLESHPASRIFHAFEESKVEVINSNLEVSQDTVLHTFVVKSEELTKEKLISALSREQTNSVQSRTSSGR

>AtbHLH014

MYNLTFSPSLSSSLLSFTQQTPAAIVSSSPPDLVLQQKLRFVVETSPDRWAYVIFWQKMFDDQSDRSYLVWVDGHFCGNKNNNSQENYTTNSIECELMMDGGDDLELFYAASFYGEDRSPRKEVSDESLVWLTGPDELRFSNYERAKEAGFHGVHTLVSIPINNGIIELGSSESIIQNRNFINRVKSIFGSGKTTKHTNQTGSYPKPAVSDHSKSGNQQFGSERKRRRKLETTRVAAATKEKHHPAVLSHVEAEKQRREKLNHRFYALRAIVPKVSRMDKASLLSDAVSYIESLKSKIDDLETEIKKMKMTETDKLDNSSSNTSPSSVEYQVNQKPSKSNRGSDLEVQVKIVGEEAIIRVQTENVNHPTSALMSALMEMDCRVQHANASRLSQVMVQDVVVLVPEGLRSEDRLRTTLVRTLSL

>AtbHLH015

MDPQQQPSSDQNLFIQEDEMTSWLHYPLRDDDFCSDLLFSAAPTATATATVSQVTAARPPVSSTNESRPPVRNFMNFSRLRGDFNNGRGGESGPLLSKAVVRESTQVSPSATPSAAASESGLTRRTDGTDSSAVAGGGAYNRKGKAVAMTAPAIEITGTSSSVVSKSEIEPEKTNVDDRKRKEREATTTDETESRSEETKQARVSTTSTKRSRAAEVHNLSERKRRDRINERMKALQELIPRCNKSDKASMLDEAIEYMKSLQLQIQMMSMGCGMMPMMYPGMQQYMPHMAMGMGMNQPIPPPSFMPFPNMLAAQRPLPTQTHMAGSGPQYPVHASDPSRVFVPNQQYDPTSGQPQYPAGYTDPYQQFRGLHPTQPPQFQNQATSYPSSSRVSSSKESEDHGNHTTG

>AtbHLH016

MSQCVPNCHIDDTPAAATTTVRSTTAADIPILDYEVAELTWENGQLGLHGLGPPRVTASSTKYSTGAGGTLESIVDQATRLPNPKPTDELVPWFHHRSSRAAMAMDALVPCSNLVHEQQSKPGGVGSTRVGSCSDGRTMGGGKRARVAPEWSGGGSQRLTMDTYDVGFTSTSMGSHDNTIDDHDSVCHSRPQMEDEEEKKAGGKSSVSTKRSRAAAIHNQSERKRRDKINQRMKTLQKLVPNSSKTDKASMLDEVIEYLKQLQAQVSMMSRMNMPSMMLPMAMQQQQQLQMSLMSNPMGLGMGMGMPGLGLLDLNSMNRAAASAPNIHANMMPNPFLPMNCPSWDASSNDSRFQSPLIPDPMSAFLACSTQPTTMEAYSRMATLYQQMQQQLPPPSNPK

>AtbHLH017

MNMSDLGWDDEDKSVVSAVLGHLASDFLRANSNSNQNLFLVMGTDDTLNKKLSSLVDWPNSENFSWNYAIFWQQTMSRSGQQVLGWGDGCCREPNEEEESKVVRSYNFNNMGAEEETWQDMRKRVLQKLHRLFGGSDEDNYALSLEKVTATEIFFLASMYFFFNHGEGGPGRCYSSGKHVWLSDAVNSESDYCFRSFMAKSAGIRTIVMVPTDAGVLELGSVWSLPENIGLVKSVQALFMRRVTQPVMVTSNTNMTGGIHKLFGQDLSGAHAYPKKLEVRRNLDERFTPQSWEGYNNNKGPTFGYTPQRDDVKVLENVNMVVDNNNYKTQIEFAGSSVAASSNPSTNTQQEKSESCTEKRPVSLLAGAGIVSVVDEKRPRKRGRKPANGREEPLNHVEAERQRREKLNQRFYALRSVVPNISKMDKASLLGDAISYIKELQEKVKIMEDERVGTDKSLSESNTITVEESPEVDIQAMNEEVVVRVISPLDSHPASRIIQAMRNSNVSLMEAKLSLAEDTMFHTFVIKSNNGSDPLTKEKLIAAFYPETSSTQPPLPSSSSQVSGDI

>AtbHLH018

MNSLVGDVPQSLSSLDDTTTCYNLDASCNKSLVEERPSKILKTTHISPNLHPFSSSNPPPPKHQPSSRILSFEKTGLHVMNHNSPNLIFSPKDEEIGLPEHKKAELIIRGTKRAQSLTRSQSNAQDHILAERKRREKLTQRFVALSALIPGLKKMDKASVLGDAIKHIKYLQESVKEYEEQKKEKTMESVVLVKKSSLVLDENHQPSSSSSSDGNRNSSSSNLPEIEVRVSGKDVLIKILCEKQKGNVIKIMGEIEKLGLSITNSNVLPFGPTFDISIIAQNNNFDMKIEDVVKNLSFGLSKLT

>AtbHLH019

MDEDFFLPDFSLVDIDFDFNIYEENNLSPDESLSNSRRADQSSKFDHQMHFECLREKPKAAVKPMMKINNKQQLISFDFSSNVISSPAAEEIIMDKLVGRGTKRKTCSHGTRSPVLAKEHVLAERKRREKLSEKFIALSALLPGLKKADKVTILDDAISRMKQLQEQLRTLKEEKEATRQMESMILVKKSKVFFDEEPNLSCSPSVHIEFDQALPEIEAKISQNDILIRILCEKSKGCMINILNTIENFQLRIENSIVLPFGDSTLDITVLAQMDKDFSMSILKDLVRNLRLAMV

>AtbHLH020

MDDSSFMDLMIDTDEYLIDDWESDFPICGETNTNPGSESGSGTGFELLAERPTKQMKTNNNMNSTSSSPSSSSSSGSRTSQVISFGSPDTKTNPVETSLNFSNQVSMDQKVGSKRKDCVNNGGRREPHLLKEHVLAERKRRQKLNERLIALSALLPGLKKTDKATVLEDAIKHLKQLQERVKKLEEERVVTKKMDQSIILVKRSQVYLDDDSSSYSSTCSAASPLSSSSDEVSIFKQTMPMIEARVSDRDLLIRVHCEKNKGCMIKILSSLEKFRLEVVNSFTLPFGNSTLVITILTKMDNKFSRPVEEVVKNIRVALAE

>AtbHLH021

MESNMQNLLEKLRPLVGARAWDYCVLWRLNEDQRFVKWMGCCCGGTELIAENGTEEFSYGGCRDVMFHHPRTKSCEFLSHLPASIPLDSGIYAETLLTNQTGWLSESSEPSFMQETICTRVLIPIPGGLVELFATRHVAEDQNVVDFVMGHCNMLMDDSVTINMMVADEVESKPYGMLSGDIQQKGSKEEDMMNLPSSYDISADQIRLNFLPQMSDYETQHLKMKSDYHHQALGYLPENGNKEMMGMNPFNTVEEDGIPVIGEPSLLVNEQQVVNDKDMNENGRVDSGSDCSDQIDDEDDPKYKKKSGKGSQAKNLMAERRRRKKLNDRLYALRSLVPRITKLDRASILGDAINYVKELQNEAKELQDELEENSETEDGSNRPQGGMSLNGTVVTGFHPGLSCNSNVPSVKQDVDLENSNDKGQEMEPQVDVAQLDGREFFVKVICEYKPGGFTRLMEALDSLGLEVTNANTTRYLSLVSNVFKVEKNDNEMVQAEHVRNSLLEITRNTSRGWQDDQMATGSMQNEKNEVDYQHYDDHQHHNGHHHPFDHQMNQSAHHHHHHQHINHYHNQ

>AtbHLH022

MGGGSRFQEPVRMSRRKQVTKEKEEDENFKSPNLEAERRRREKLHCRLMALRSHVPIVTNMTKASIVEDAITYIGELQNNVKNLLETFHEMEEAPPEIDEEQTDPMIKPEVETSDLNEEMKKLGIEENVQLCKIGERKFWLKIITEKRDGIFTKFMEVMRFLGFEIIDISLTTSNGAILISASVQTQELCDVEQTKDFLLEVMRSNP

>AtbHLH023

MTWKPKMLILSHDLISPEKYIMGEDDIVELLGKSSQVVTSSQTQTPSCDPPLILRGSGSGDGEGNGPLPQPPPPLYHQQSLFIQEDEMASWLHQPNRQDYLYSQLLYSGVASTHPQSLASLEPPPPPRAQYILAADRPTGHILAERRAENFMNISRQRGNIFLGGVEAVPSNSTLLSSATESIPATHGTESRATVTGGVSRTFAVPGLGPRGKAVAIETAGTQSWGLCKAETEPVQRQPATETDITDERKRKTREETNVENQGTEEARDSTSSKRSRAAIMHKLSERRRRQKINEMMKALQELLPRCTKTDRSSMLDDVIEYVKSLQSQIQMFSMGHVMIPPMMYAGNIQQQYMPHMAMGMNRPPAFIPFPRQAHMAEGVGPVDLFRENEETEQETMSLLLREDKRTKQKMFS

>AtbHLH024

MISQREEREEKKQRVMGDKKLISSSSSSSVYDTRINHHLHHPPSSSDEISQFLRHIFDRSSPLPSYYSPATTTTTASLIGVHGSGDPHADNSRSLVSHHPPSDSVLMSKRVGDFSEVLIGGGSGSAAACFGFSGGGNNNNVQGNSSGTRVSSSSVGASGNETDEYDCESEEGGEAVVDEAPSSKSGPSSRSSSKRCRAAEVHNLSEKRRRSRINEKMKALQSLIPNSNKTDKASMLDEAIEYLKQLQLQVQMLTMRNGINLHPLCLPGTTLHPLQLSQIRPPEATNDPLLNHTNQFASTSNAPEMINTVASSYALEPSIRSHFGPFPLLTSPVEMSREGGLTHPRLNIGHSNANITGEQALFDGQPDLKDRIT

>AtbHLH025

MSILSTRWFSEQEIEENSIIQQFHMNSIVGEVQEAQYIFPHSFTTNNDPSYDDLIEMKPPKILETTYISPSSHLPPNSKPHHIHRHSSSRILSFEDYGSNDMEHEYSPTYLNSIFSPKLEAQVQPHQKSDEFNRKGTKRAQPFSRNQSNAQDHIIAERKRREKLTQRFVALSALVPGLKKMDKASVLGDALKHIKYLQERVGELEEQKKERRLESMVLVKKSKLILDDNNQSFSSSCEDGFSDLDLPEIEVRFSDEDVLIKILCEKQKGHLAKIMAEIEKLHILITNSSVLNFGPTLDITIIAKKESDFDMTLMDVVKSLRSALSNFI

>AtbHLH026

MSNNQAFMELGWRNDVGSLAVKDQGMMSERARSDEDRLINGLKWGYGYFDHDQTDNYLQIVPEIHKEVENAKEDLLVVVPDEHSETDDHHHIKDFSERSDHRFYLRNKHENPKKRRIQVLSSDDESEEFTREVPSVTRKGSKRRRRDEKMSNKMRKLQQLVPNCHKTDKVSVLDKTIEYMKNLQLQLQMMSTVGVNPYFLPATLGFGMHNHMLTAMASAHGLNPANHMMPSPLIPALNWPLPPFTNISFPHSSSQSLFLTTSSPASSPQSLHGLVPYFPSFLDFSSHAMRRL

>AtbHLH027

MEDLDHEYKNYWETTMFFQNQELEFDSWPMEEAFSGSGESSSPDGAATSPASSKNVVSERNRRQKLNQRLFALRSVVPNISKLDKASVIKDSIDYMQELIDQEKTLEAEIRELESRSTLLENPVRDYDCNFAETHLQDFSDNNDMRSKKFKQMDYSTRVQHYPIEVLEMKVTWMGEKTVVVCITCSKKRETMVQLCKVLESLNLNILTTNFSSFTSRLSTTLFLQADEEESSAVEAKIQMAIAAYNDPNCLINF

>AtbHLH028

MINTDDNLLMIEALLTSDPSPPLLPANLSLETTLPKRLHAVLNGTHEPWSYAIFWKPSYDDFSGEAVLKWGDGVYTGGNEEKTRGRLRRKKTILSSPEEKERRSNVIRELNLMISGEAFPVVEDDVSDDDDVEVTDMEWFFLVSMTWSFGNGSGLAGKAFASYNPVLVTGSDLIYGSGCDRAKQGGDVGLQTILCIPSHNGVLELASTEEIRPNSDLFNRIRFLFGGSKYFSGAPNSNSELFPFQLESSCSSTVTGNPNPSPVYLQNRYNLNFSTSSSTLARAPCGDVLSFGENVKQSFENRNPNTYSDQIQNVVPHATVMLEKKKGKKRGRKPAHGRDKPLNHVEAERMRREKLNHRFYALRAVVPNVSKMDKTSLLEDAVCYINELKSKAENVELEKHAIEIQFNELKEIAGQRNAIPSVCKYEEKASEMMKIEVKIMESDDAMVRVESRKDHHPGARLMNALMDLELEVNHASISVMNDLMIQQANVKMGLRIYKQEELRDLLMSKIS

>AtbHLH029

MEGRVNALSNINDLELHNFLVDPNFDQFINLIRGDHQTIDENPVLDFDLGPLQNSPCFIDENQFIPTPVDDLFDELPDLDSNVAESFRSFDGDSVRAGGEEDEEDYNDGDDSSATTTNNDGTRKTKTDRSRTLISERRRRGRMKDKLYALRSLVPNITKMDKASIVGDAVLYVQELQSQAKKLKSDIAGLEASLNSTGGYQEHAPDAQKTQPFRGINPPASKKIIQMDVIQVEEKGFYVRLVCNKGEGVAPSLYKSLESLTSFQVQNSNLSSPSPDTYLLTYTLDGTCFEQSLNLPNLKLWITGSLLNQGFEFIKSFT

>AtbHLH030

MCAKKEEEEEEEEDSSEAMNNIQNYQNDLFFHQLISHHHHHHHDPSQSETLGASGNVGSGFTIFSQDSVSPIWSLPPPTSIQPPFDQFPPPSSSPASFYGSFFNRSRAHHQGLQFGYEGFGGATSAAHHHHEQLRILSEALGPVVQAGSGPFGLQAELGKMTAQEIMDAKALAASKSHSEAERRRRERINNHLAKLRSILPNTTKTDKASLLAEVIQHVKELKRETSVISETNLVPTESDELTVAFTEEEETGDGRFVIKASLCCEDRSDLLPDMIKTLKAMRLKTLKAEITTVGGRVKNVLFVTGEESSGEEVEEEYCIGTIEEALKAVMEKSNVEESSSSGNAKRQRMSSHNTITIVEQQQQYNQR

>AtbHLH031

MDPSGMMNEGGPFNLAEIWQFPLNGVSTAGDSSRRSFVGPNQFGDADLTTAANGDPARMSHALSQAVIEGISGAWKRREDESKSAKIVSTIGASEGENKRQKIDEVCDGKAEAESLGTETEQKKQQMEPTKDYIHVRARRGQATDSHSLAERARREKISERMKILQDLVPGCNKVIGKALVLDEIINYIQSLQRQVEFLSMKLEAVNSRMNPGIEVFPPKEFGQQAFENPEIQFGSQSTREYSRGASPEWLHMQIGSGGFERTS

>AtbHLH032

MYAMKEEDCLQTFHNLQDYQDQFHLHHHPQILPWSSTSLPSFDPLHFPSNPTRYSDPVHYFNRRASSSSSSFDYNDGFVSPPPSMDHPQNHLRILSEALGPIMRRGSSFGFDGEIMGKLSAQEVMDAKALAASKSHSEAERRRRERINTHLAKLRSILPNTTKTDKASLLAEVIQHMKELKRQTSQITDTYQVPTECDDLTVDSSYNDEEGNLVIRASFCCQDRTDLMHDVINALKSLRLRTLKAEIATVGGRVKNILFLSREYDDEEDHDSYRRNFDGDDVEDYDEERMMNNRVSSIEEALKAVIEKCVHNNDESNDNNNLEKSSSGGIKRQRTSKMVNRCYN

>AtbHLH033

MESREDSFISKEKKSTMKKEKQAIASQRNRRRVIKNRGNGKRLIASLSQRKRRRIPRGRGNEKAVFAPSSLPNDVVEEIFLRLPVKAIIQLKSLSKQWRSTIESRSFEERHLKIVERSRVDFPQVMVMSEEYSLKGSKGNQPRPDTDIGFSTICLESASILSSTLITFPQGFQHRIYASESCDGLFCIHSLKTQAIYVVNPATRWFRQLPPARFQILMQKLYPTQDTWIDIKPVVCYTAFVKANDYKLVWLYNSDASNPNLGVTKCEVFDFRANAWRYLTCTPSYRIFPDQVPAATNGSIYWFTEPYNGEIKVVALDIHTETFRVLPKINPAIASSDPDHIDMCTLDNGLCMSKRESDTLVQEIWRLKSSEDSWEKFDMNSDGVWLDGSGESPEVNNGEAASWVRNPDEDWFNNPPPPQHTNQNDFRFNGGFPLNPSENLLLLLQQSIDSSSSSSPLLHPFTLDAASQQQQQQQQQQEQSFLATKACIVSLLNVPTINNNTFDDFGFDSGFLGQQFHGNHQSPNSMNFTGLNHSVPDFLPAPENSSGSCGLSPLFSNRAKVLKPLQVMASSGSQPTLFQKRAAMRQSSSSKMCNSESSSEMRKSSYEREIDDTSTGIIDISGLNYESDDHNTNNNKGKKKGMPAKNLMAERRRRKKLNDRLYMLRSVVPKISKMDRASILGDAIDYLKELLQRINDLHTELESTPPSSSSLHPLTPTPQTLSYRVKEELCPSSSLPSPKGQQPRVEVRLREGKAVNIHMFCGRRPGLLLSTMRALDNLGLDVQQAVISCFNGFALDVFRAEQCQEDHDVLPEQIKAVLLDTAGYAGLV

>AtbHLH034

MYPSIEDDDDLLAALCFDQSNGVEDPYGYMQTNEDNIFQDFGSCGVNLMQPQQEQFDSFNGNLEQVCSSFRGGNNGVVYSSSIGSAQLDLAASFSGVLQQETHQVCGFRGQNDDSAVPHLQQQQGQVFSGVVEINSSSSVGAVKEEFEEECSGKRRRTGSCSKPGTKACREKLRREKLNDKFMDLSSVLEPGRTPKTDKSAILDDAIRVVNQLRGEAHELQETNQKLLEEIKSLKADKNELREEKLVLKAEKEKMEQQLKSMVVPSPGFMPSQHPAAFHSHKMAVAYPYGYYPPNMPMWSPLPPADRDTSRDLKNLPPVA

>AtbHLH035

MEDIVDQELSNYWEPSSFLQNEDFEYDSWPLEEAISGSYDSSSPDGAASSPASKNIVSERNRRQKLNQRLFALRSVVPNITKMDKASIIKDAISYIEGLQYEEKKLEAEIRELESTPKSSLSFSKDFDRDLLVPVTSKKMKQLDSGSSTSLIEVLELKVTFMGERTMVVSVTCNKRTDTMVKLCEVFESLNLKILTSNLTSFSGMIFHTVFIEADEEEQEVLRLKIETGIGAYNETQSPTLSIDSLY

>AtbHLH036

MEKMMHRETERQRRQEMASLYASLRSLLPLHFIKGKRSTSDQVNEAVNYIKYLQRKIKELSVRRDDLMVLSRGSLLGSSNGDFKEDVEMISGKNHVVVRQCLVGVEIMLSSRCCGGQPRFSSVLQVLSEYGLCLLNSISSIVDDRLVYTIQAEVNDMALMIDLAELEKRLIRMK

>AtbHLH037

MDNSDILMNMMMQQMEKLPEHFSNSNPNPNPHNIMMLSESNTHPFFFNPTHSHLPFDQTMPHHQPGLNFRYAPSPSSSLPEKRGGCSDNANMAAMREMIFRIAVMQPIHIDPESVKPPKRKNVRISKDPQSVAARHRRERISERIRILQRLVPGGTKMDTASMLDEAIHYVKFLKKQVQSLEEHAVVNGGGMTAVAGGALAGTVGGGYGGKGCGIMRSDHHQMLGNAQILR

>AtbHLH038

MCALVPSFFTNFGWPSTNQYESYYGAGDNLNNGTFLELTVPQTYEVTHHQNSLGVSVSSEGNEIDNNPVVVKKLNHNASERDRRKKINTLFSSLRSCLPASDQSKKLSIPETVSKSLKYIPELQQQVKRLIQKKEEILVRVSGQRDFELYDKQQPKAVASYLSTVSATRLGDNEVMVQVSSSKIHNFSISNVLGGIEEDGFVLVDVSSSRSQGERLFYTLHLQVENMDDYKINCEELSERMLYLYEKCENSFN

>AtbHLH039

MCALVPPLFPNFGWPSTGEYDSYYLAGDILNNGGFLDFPVPEETYGAVTAVTQHQNSFGVSVSSEGNEIDNNPVVVKKLNHNASERDRRRKINSLFSSLRSCLPASGQSKKLSIPATVSRSLKYIPELQEQVKKLIKKKEELLVQISGQRNTECYVKQPPKAVANYISTVSATRLGDNEVMVQISSSKIHNFSISNVLSGLEEDRFVLVDMSSSRSQGERLFYTLHLQVEKIENYKLNCEELSQRMLYLYEECGNSYI

>AtbHLH040

MENGMYKKKGVCDSCVSSKSRSNHSPKRSMMEPQPHHLLMDWNKANDLLTQEHAAFLNDPHHLMLDPPPETLIHLDEDEEYDEDMDAMKEMQYMIAVMQPVDIDPATVPKPNRRNVRISDDPQTVVARRRRERISEKIRILKRIVPGGAKMDTASMLDEAIRYTKFLKRQVRILQPHSQIGAPMANPSYLCYYHNSQP

>AtbHLH041

MMHLILSCSYLISMDGYYNEASEEPSSSSSSGSLARSLFHEYRQSVIPLQNGHVPSMAFMNNLPYVEIRPQESQRLAFNDTQRLFYQMKIEASLREWFPEDFNRKSSPANSDYLRPPHYPSSSSSSLSPNNISEYSSLLFPLIPKPSTTTEAVNVPVLPPLAPINMIHPQHQEPLFRNRQREEEAMTQAILAVLTGPSSPPSTSSSPQRKGRATAFKRYYSMISDRGRAPLPSVRKQSMMTRAMSFYNRLNINQRERFTRENATTHGEGSGGSGGGGRYTSGPSATQLQHMISERKRREKLNESFQALRSLLPPGTKKDKASVLSIAREQLSSLQGEISKLLERNREVEAKLAGEREIENDLRPEERFNVRIRHIPESTSRERTLDLRVVLRGDIIRVDDLMIRLLEFLKQINNVSLVSIEARTLARAEGDTSIVLVISLRLKIEGEWDESAFQEAVRRVVADLAH

>AtbHLH042

MDESSIIPAEKVAGAEKKELQGLLKTAVQSVDWTYSVFWQFCPQQRVLVWGNGYYNGAIKTRKTTQPAEVTAEEAALERSQQLRELYETLLAGESTSEARACTALSPEDLTETEWFYLMCVSFSFPPPSGMPGKAYARRKHVWLSGANEVDSKTFSRAILAKSAKIQTVVCIPMLDGVVELGTTKKVREDVEFVELTKSFFYDHCKTNPKPALSEHSTYEVHEEAEDEEEVEEEMTMSEEMRLGSPDDEDVSNQNLHSDLHIESTHTLDTHMDMMNLMEEGGNYSQTVTTLLMSHPTSLLSDSVSTSSYIQSSFATWRVENGKEHQQVKTAPSSQWVLKQMIFRVPFLHDNTKDKRLPREDLSHVVAERRRREKLNEKFITLRSMVPFVTKMDKVSILGDTIAYVNHLRKRVHELENTHHEQQHKRTRTCKRKTSEEVEVSIIENDVLLEMRCEYRDGLLLDILQVLHELGIETTAVHTSVNDHDFEAEIRAKVRGKKASIAEVKRAIHQVIIHDTNL

>AtbHLH043

MNNYNMNPSLFQNYTWNNIINSSNNNNKNDDHHHQHNNDPIGMAMDQYTQLHIFNPFSSSHFPPLSSSLTTTTLLSGDQEDDEDEEEPLEELGAMKEMMYKIAAMQSVDIDPATVKKPKRRNVRISDDPQSVAARHRRERISERIRILQRLVPGGTKMDTASMLDEAIRYVKFLKRQIRLLNNNTGYTPPPPQDQASQAVTTSWVSPPPPPSFGRGGRGVGELI

>AtbHLH044

MANFENLSSDFQTIAMDIYSSITQAADLNNNNSNLHFQTFHPSSTSLESLFLHHHQQQLLHFPGNSPDSSNNFSSTSSFLHSDHNIVDETKKRKALLPTLSSSETSGVSDNTNVIATETGSLRRGKRLKKKKEEEDEKEREVVHVRARRGQATDSHSLAERVRRGKINERLRCLQDMVPGCYKAMGMATMLDEIINYVQSLQNQVEFLSMKLTAASSFYDFNSETDAVDSMQRAKARETVEMGRQTRDGSPVFHLSTWSL

>AtbHLH045

MSHIAVERNRRRQMNEHLKSLRSLTPCFYIKRGDQASIIGGVIEFIKELQQLVQVLESKKRRKTLNRPSFPYDHQTIEPSSLGAATTRVPFSRIENVMTTSTFKEVGACCNSPHANVEAKISGSNVVLRVVSRRIVGQLVKIISVLEKLSFQVLHLNISSMEETVLYFFVVKIGLECHLSLEELTLEVQKSFVSDEVIVSTN

>AtbHLH046

MMFNANERNVLDENSNSNCSSYAAASSGFTLWDESASGKKGQTRKENSVGERVNMRADVAATVGQWPVAERRSQSLTNNHMSGFSSLSSSQGSVLKSQSFMDMIRSAKGSSQEDDLDDEEDFIMKKESSSTSQSHRVDLRVKADVRGSPNDQKLNTPRSKHSATEQRRRSKINDRFQMLRQLIPNSDQKRDKASFLLEVIEYIQFLQEKADKYVTSYQGWNHEPAKLLNWQSNNNQQLVPEGVAFAPKLEEEKNNIPVSVLATAQGVVIDHPTTATTSPFPLSIQSNSFFSPVIAGNPVPQFHARVASSEAVEPSPSSRSQKEEEDEEVLEGNIRISSVYSQGLVKTLREALENSGVDLTKASISVEIELAKQSSSSSFKDHEVREPVSRTRNDNVKQTRKPKRLKTGQ

>AtbHLH047

MVSKTPSTSSDEANATADERCRKGKVPKRINKAVRERLKREHLNELFIELADTLELNQQNSGKASILCEATRFLKDVFGQIESLRKEHASLLSESSYVTTEKNELKEETSVLETEISKLQNEIEARANQSKPDLNTSPAPEYHHHHYQQQHPERVSQFPGLPIFQGPGFQQSATTLHPPATVLVLPIQPDPQTQDISEMTQAQQPLMFNSSNVSKPCPRYASAADSWSSRLLGERLKASE

>AtbHLH048

MDLTQGFRARSGVVGPVAGLESLNFSDEFRHLVTTMPPETTGGSFTALLEMPVTQAMELLHFPDSSSSQARTVTSGDISPTTLHPFGALTFPSNSLLLDRAARFSVIATEQNGNFSGETANSLPSNPGANLDRVKAEPAETDSMVENQNQSYSSGKRKEREKKVKSSTKKNKSSVESDKLPYVHVRARRGQATDNHSLAERARREKINARMKLLQELVPGCDKIQGTALVLDEIINHVQTLQRQVEMLSMRLAAVNPRIDFNLDSILASENGSLMDGSFNAESYHQLQQWPFDGYHQPEWGREEDHHQANFSMGSATLHPNQVKMEL

>AtbHLH049

MDLSAKDEFSAEKRNPDNYDSVNNPSGDWRVDSYPSENLISAGPASCSPSQMMDSFGQTLWYDPTSVQAVGYAGFNGGNASSSSFRGSIDRSLEMGWNLPNLLPPKGNGLFLPNASSFLPPSMAQFPADSGFIERAARFSLFSGGNFSDMVNQPLGNSEAIGLFLQGGGTMQGQCQSNELNVGEPHNDVSVAVKESTVRSSEQAKPNVPGSGNVSEDTQSSGGNGQKGRETSSNTKKRKRNGQKNSEAAQSHRSQQSEEEPDNNGDEKRNDEQSPNSPGKKSNSGKQQGKQSSDPPKDGYIHVRARRGQATNSHSLAERVRREKISERMKFLQDLVPGCNKVTGKAVMLDEIINYVQSLQRQVEFLSMKLATVNPQMDFNLEGLLAKDALQLRAGSSSTTPFPPNMSMAYPPLPHGFMQQTLSSIGRTITSPLSPMNGGFKRQETNGWEGDLQNVIHINYGAGDVTPDPQAAATASLPAANMKVEP

>AtbHLH050

MANLSSDFQTFTMDDPIRQLAELSNTLHHFQTFPPPFSSSLDSLFFHNQFPDHFPGKSLENNFHQGIFFPSNIQNNEESSSQFDTKKRKSLMEAVSTSENSVSDQTLSTSSAQVSINGNISTKNNSSRRGKRSKNREEEKEREVVHVRARRGQATDSHSIAERVRRGKINERLKCLQDIVPGCYKTMGMATMLDEIINYVQSLQNQVEFLSMKLTAASSYYDFNSETDAVESMQKAKAREAVEMGQGRDGSSVFHSSSWTL

>AtbHLH051

MENSYDSSKWSDSTTPYMVSWSLQSESSDSDWNRFNLGFSSSSFGGNFPADDCVGGIEKAESLSRSHRLAEKRRRDRINSHLTALRKLVPNSDKLDKAALLATVIEQVKELKQKAAESPIFQDLPTEADEVTVQPETISDFESNTNTIIFKASFCCEDQPEAISEIIRVLTKLQLETIQAEIISVGGRMRINFILKDSNCNETTNIAASAKALKQSLCSALNRITSSSTTTSSVCRIRSKRQRWFLSSHYSHNE

>AtbHLH052

MIIPETDSFFFQEQPQHQPLYPDEALSPSLFGFDHYDHFYESFLPSQEIFLPSPKTRVFNESQELDSFHTPKHQKLIDSSFHFNSHDPFSPSPESNYLLDSYITEASNISKFQAPDFSSTFKVGWTEQGDTKKRELSAQSIAARKRRRRITEKTQELGKLIPGSQKHNTAEMFNAAAKYVKFLQAQIEILQLKQTKMQTLDSSKVGREMQFLLGSQEIQEKLSTEEVCVVPREMVQVLKAEECILTNPKISRDINKLLSTNLMN

>AtbHLH053

MSMDCLSYFFNYDPPVQLQDCFIPEMDMIIPETDSFFFQSQPQLEFHQPLFQEEAPSQTHFDPFCDQFLSPQEIFLPNPKNEIFNETHDLDFFLPTPKRQRLVNSSYNCNTQNHFQSRNPNFFDPFGDTDFVPESCTFQEFRVPDFSLAFKVGRGDQDDSKKPTLSSQSIAARGRRRRIAEKTHELGKLIPGGNKLNTAEMFQAAAKYVKFLQSQVGILQLMQTTKKGSSNVQMETQYLLESQAIQEKLSTEEVCLVPCEMVQDLTTEETICRTPNISREINKLLSKHLAN

>AtbHLH054

MDVFVDGELESLLGMFNFDQCSSSKEERPRDELLGLSSLYNGHLHQHQHHNNVLSSDHHAFLLPDMFPFGAMPGGNLPAMLDSWDQSHHLQETSSLKRKLLDVENLCKTNSNCDVTRQELAKSKKKQRVSSESNTVDESNTNWVDGQSLSNSSDDEKASVTSVKGKTRATKGTATDPQSLYARKRREKINERLKTLQNLVPNGTKVDISTMLEEAVHYVKFLQLQIKLLSSDDLWMYAPLAYNGLDMGFHHNLLSRLM

>AtbHLH055

MTLSEIMKGDDEPKNKRAKHKELERQRRQENTSLFKILRYLLPSQYIKGKRSSADHVLEAVNYIKDLQKKIKEVSEKRDRIKRSITHPSSRGEFSIRSLASSTCSCVGDTNIAVVVRPCLIGLEIVVSCCNRHESCLSSVLQLLAQEQCFNIVSCISTRLHQGFIHTIASEVEEGIEVYFSELQEKIIKIGTSRVTTR

>AtbHLH056

MQFDESDARVWIWFEIRREDDIVELLWQSGQVVGTNQTHRQSYDPPPILRGSGSGRGEENAPLSQPPPHLHQQNLFIQEGEMYSWLHHSYRQNYFCSELLNSTPATHPQSSISLAPRQTIATRRAENFMNFSWLRGNIFTGGRVDEAGPSFSVVRESMQVGSNTTPPSSSATESCVIPATEGTASRVSGTLAAHDLGRKGKAVAVEAAGTPSSGVCKAETEPVQIQPATESKLKAREETHGTEEARGSTSRKRSRTAEMHNLAERRRREKINEKMKTLQQLIPRCNKSTKVSTLDDAIEYVKSLQSQIQMMSTGQGMMSPMMNAGNTQQFMPHMAMDMNRPPPFIPFPGTSFPMPAQMAGVGPSYPAPRYPFPNIQTFDPSRVRLPSPQPNPVSNQPQFPAYMNPYSQFAGPHQLQQPPPPPFQVTLYHGIHCVVAGNPYV

>AtbHLH057

MSGLMSFGELEDQFGQISDTTMEEKIPFLQMLQCIEHPFTTTEPNQFLQSLLQIQTLESKSCLTLETNIKRDPGQTDDPEKDPRTENGAVTVKEKRKRKRTRAPKNKDEVENQRMTHIAVERNRRRQMNEHLNSLRSLMPPSFLQRGDQASIVGGAIDFIKELEQLLQSLEAEKRKDGTDETPKTASCSSSSSLACTNSSISSVSTTSENGFTARFGGGDTTEVEATVIQNHVSLKVRCKRGKRQILKAIVSIEELKLAILHLTISSSFDFVIYSFNLKMEDGCKLGSADEIATAVHQIFEQINGEVMWSNLSRT

>AtbHLH058

MDLSVLDRLKWLQQQQMVSPEFLQILGSDGREELKRVESYLGNNNDELQSFRHFPEFGPDYDTTDGCISRTSSFHMEPVKNNGHSRAITLQNKRKPEGKTEKREKKKIKAEDETEPSMKGKSNMSNTETSSEIQKPDYIHVRARRGEATDRHSLAERARREKISKKMKCLQDIVPGCNKVTGKAGMLDEIINYVQSLQQQVEFLSMKLSVINPELECHIDDLSAKQFQAYFTGPPEGDSKQSIMADFRSFPLHQQGSLDYSVINSDHTTSLGAKDHTSSSWETHSQCLYNSLRTDSVSNFFSLK

>AtbHLH059

MASNNPHDNLSDQTPSDDFFEQILGLPNFSASSAAGLSGVDGGLGGGAPPMMLQLGSGEEGSHMGGLGGSGPTGFHNQMFPLGLSLDQGKGPGFLRPEGGHGSGKRFSDDVVDNRCSSMKPVFHGQPMQQPPPSAPHQPTSIRPRVRARRGQATDPHSIAERLRRERIAERIRALQELVPTVNKTDRAAMIDEIVDYVKFLRLQVKVLSMSRLGGAGAVAPLVTDMPLSSSVEDETGEGGRTPQPAWEKWSNDGTERQVAKLMEENVGAAMQLLQSKALCMMPISLAMAIYHSQPPDTSSVVKPENNPPQ

>AtbHLH060

MDLTGGFGARSGGVGPCREPIGLESLHLGDEFRQLVTTLPPENPGGSFTALLELPPTQAVELLHFTDSSSSQQAAVTGIGGEIPPPLHSFGGTLAFPSNSVLMERAARFSVIATEQQNGNISGETPTSSVPSNSSANLDRVKTEPAETDSSQRLISDSAIENQIPCPNQNNRNGKRKDFEKKGKSSTKKNKSSEENEKLPYVHVRARRGQATDSHSLAERARREKINARMKLLQELVPGCDKGTDFGGKIKIKVCFGVHLLMISGKKVAIFLWKVSCEDLIDCSFSPPRIQGTALVLDEIINHVQSLQRQVEMLSMRLAAVNPRIDFNLDTILASENGSLMDGSFNAAPMQLAWPQQAIETEQSFHHRQLQQPPTQQWPFDGLNQPVWGREEDQAHGNDNSNLMAVSENVMVASANLHPNQVKMEL

>AtbHLH061

METELTQLRKQESNNLNGVNGGFMAIDQFVPNDWNFDYLCFNNLLQEDDNIDHPSSSSLMNLISQPPPLLHQPPQPSSPLYDSPPLSSAFDYPFLEDIIHSSYSPPPLILPASQENTNNYSPLMEESKSFISIGETNKKRSNKKLEGQPSKNLMAERRRRKRLNDRLSLLRSIVPKITKMDRTSILGDAIDYMKELLDKINKLQEDEQELGSNSHLSTLITNESMVRNSLKFEVDQREVNTHIDICCPTKPGLVVSTVSTLETLGLEIEQCVISCFSDFSLQASCFEVGEQRYMVTSEATKQALIRNAGYGGRCL

>AtbHLH062

MENELFMNAGVSHPPVMTSPSSSSAMLKWVSMETQPVDPSLSRNLFWEKSTEQSIFDSALSSLVSSPTPSNSNFSVGGVGGENVIMRELIGKLGNIGDIYGITASNGNSCYATPMSSPPPGSMMETKTTTPMAELSGDPGFAERAARFSCFGSRSFNSRTNSPFPINNEPPITTNEKMPRVSSSPVFKPLASHVPAGESSGELSRKRKTKSKQNSPSAVSSSKEIEEKEDSDPKRCKKSEENGDKTKSIDPYKDYIHVRARRGQATDSHSLAERVRREKISERMKLLQDLVPGCNKVTGKALMLDEIINYVQSLQRQVEFLSMKLSSVNTRLDFNMDALLSKDIFPSSNNLMHHQQVLQLDSSAETLLGDHHNKNLQLNPDISSNNVINPLETSETRSFISHLPTLAHFTDSISQYSTFSEDDLHSIIHMGFAQNRLQELNQGSSNQVPSHMKAEL

>AtbHLH063

MNGAIGGDLLLNFPDMSVLERQRAHLKYLNPTFDSPLAGFFADSSMITGGEMDSYLSTAGLNLPMMYGETTVEGDSRLSISPETTLGTGNFKKRKFDTETKDCNEKKKKMTMNRDDLVEEGEEEKSKITEQNNGSTKSIKKMKHKAKKEENNFSNDSSKVTKELEKTDYIHVRARRGQATDSHSIAERVRREKISERMKFLQDLVPGCDKITGKAGMLDEIINYVQSLQRQIEFLSMKLAIVNPRPDFDMDDIFAKEVASTPMTVVPSPEMVLSGYSHEMVHSGYSSEMVNSGYLHVNPMQQVNTSSDPLSCFNNGEAPSMWDSHVQNLYGNLGV

>AtbHLH064

MLEGLVSQESLSLNSMDMSVLERLKWVQQQQQQLQQVVSHSSNNSPELLQILQFHGSNNDELLESSFSQFQMLGSGFGPNYNMGFGPPHESISRTSSCHMEPVDTMEVLLKTGEETRAVALKNKRKPEVKTREEQKTEKKIKVEAETESSMKGKSNMGNTEASSDTSKETSKGASENQKLDYIHVRARRGQATDRHSLAERARREKISKKMKYLQDIVPGCNKVTGKAGMLDEIINYVQCLQRQVEFLSMKLAVLNPELELAVEDVSVKQAYFTNVVASKQSIMVDVPLFPLDQQGSLDLSAINPNQTTSIEAPSGSWETQSQSLYNTSSLGFHY

>AtbHLH065

MEQVFADWNFEDNFHMSTNKRSIRPEDELVELLWRDGQVVLQSQARREPSVQVQTHKQETLRKPNNIFLDNQETVQKPNYAALDDQETVSWIQYPPDDVIDPFESEFSSHFFSSIDHLGGPEKPRTIEETVKHEAQAMAPPKFRSSVITVGPSHCGSNQSTNIHQATTLPVSMSDRSKNVEERLDTSSGGSSGCSYGRNNKETVSGTSVTIDRKRKHVMDADQESVSQSDIGLTSTDDQTMGNKSSQRSGSTRRSRAAEVHNLSERRRRDRINERMKALQELIPHCSRTDKASILDEAIDYLKSLQMQLQVMWMGSGMAAAAAAAASPMMFPGVQSSPYINQMAMQSQMQLSQFPVMNRSAPQNHPGLVCQNPVQLQLQAQNQILSEQLARYMGGIPQMPPAGNQTVQQQPADMLGFGSPAGPQSQLSAPATTDSLHMGKIG

>AtbHLH066

MMNSSLLTPSSSSSSHIQTPSTTFDHEDFLDQIFSSAPWPSVVDDAHPLPSDGFHGHDVDSRNQPIMMMPLNDGSSVHALYNGFSVAGSLPNFQIPQGSGGGLMNQQGQTQTQTQPQASASTATGGTVAAPPQSRTKIRARRGQATDPHSIAERLRRERIAERMKALQELVPNGNKTDKASMLDEIIDYVKFLQLQVKVLSMSRLGGAASVSSQISEAGGSHGNASSAMVGGSQTAGNSNDSVTMTEHQVAKLMEEDMGSAMQYLQGKGLCLMPISLATAISTATCHSRNPLIPGAVADVGGPSPPNLSGMTIQSTSTKMGSGNGKLNGNGVTERSSSIAVKEAVSVSKA

>AtbHLH067

MERFQGHINPCFFDRKPDVRSLEVQGFAEAQSFAFKEKEEESLQDTVPFLQMLQSEDPSSFFSIKEPNFLTLLSLQTLKEPWELERYLSLEDSQFHSPVQSETNRFMEGANQAVSSQEIPFSQANMTLPSSTSSPLSAHSRRKRKINHLLPQEMTREKRKRRKTKPSKNNEEIENQRINHIAVERNRRRQMNEHINSLRALLPPSYIQRGDQASIVGGAINYVKVLEQIIQSLESQKRTQQQSNSEVVENALNHLSGISSNDLWTTLEDQTCIPKIEATVIQNHVSLKVQCEKKQGQLLKGIISLEKLKLTVLHLNITTSSHSSVSYSFNLKMEDECDLESADEITAAVHRIFDIPTI

>AtbHLH068

MNRGVLESSPVQQLMAAGNPNWWNVSGGMRPPPPLMGHQQAPLPPHMTPNNNYLRPRMMPTPFPHFLPSPATSSSSSSSSPSLPNNPNLSSWLESNDLPPESWSLSQLLLGGLMMGEEERLEMMNHHNHHDEQQHHGFQGKIRLENWEEQVLSHQQASMVAVDIKQEGNINNNNGYVISSPNSPPNKSCVTTTTTTSLNSNDDNINNNNNMLDFSSNHNGLHLSEGRHTPPDRSSECNSLEIGGSTNKKPRLQPSPSSQSTLKVRKEKLGGRIAALHQLVSPFGKTDTASVLSEAIGYIRFLQSQIEALSHPYFGTTASGNMRHQQHLQGDRSCIFPEDPGQLVNDQCMKRRGASSSSTDNQNASEEPKKDLRSRGLCLVPISCTLQVGSDNGADYWAPALGSAGFH

>AtbHLH069

MNSSSLLTPSSSPSPHLQSPATFDHDDFLHHIFSSTPWPSSVLDDTPPPTSDCAPVTGFHHHDADSRNQITMIPLSHNHPNDALFNGFSTGSLPFHLPQGSGGQTQTQSQATASATTGGATAQPQTKPKVRARRGQATDPHSIAERLRRERIAERMKSLQELVPNGNKTDKASMLDEIIDYVKFLQLQVKVLSMSRLGGAASASSQISEDAGGSHENTSSSGEAKMTEHQVAKLMEEDMGSAMQYLQGKGLCLMPISLATTISTATCPSRSPFVKDTGVPLSPNLSTTIVANGNGSSLVTVKDAPSVSKP

>AtbHLH070

MFVLRVSNQSFKLHQQVQCKDEIFCLDQKVNVRRSLQVQETVEDHQSFALEEEEQQLSTPSLLQDTTIPFLQMLQQSEDPSPFLSFKDPSFLALLSLQTLEKPWELENYLPHEVPEFHSPIHSETNHYYHNPSLEGVNEAISNQELPFNPLENARSRRKRKNNNLASLMTREKRKRRRTKPTKNIEEIESQRMTHIAVERNRRRQMNVHLNSLRSIIPSSYIQRGDQASIVGGAIDFVKILEQQLQSLEAQKRSQQSDDNKEQIPEDNSLRNISSNKLRASNKEEQSSKLKIEATVIESHVNLKIQCTRKQGQLLRSIILLEKLRFTVLHLNITSPTNTSVSYSFNLKMEDECNLGSADEITAAIRQIFDS

>AtbHLH071

MTLEALSSNGLLNFLLSETLSPTPFKSLVDLEPLPENDVIISKNTISEISNQEPPPQRQPPATNRGKKRRRRKPRVCKNEEEAENQRMTHIAVERNRRRQMNQHLSVLRSLMPQPFAHKGDQASIVGGAIDFIKELEHKLLSLEAQKHHNAKLNQSVTSSTSQDSNGEQENPHQPSSLSLSQFFLHSYDPSQENRNGSTSSVKTPMEDLEVTLIETHANIRILSRRRGFRWSTLATTKPPQLSKLVASLQSLSLSILHLSVTTLDNYAIYSISAKVEESCQLSSVDDIAGAVHHMLSIIEEEPFCCSSMSELPFDFSLNHSNVTHSL

>AtbHLH072

MSNYGVKELTWENGQLTVHGLGDEVEPTTSNNPIWTQSLNGCETLESVVHQAALQQPSKFQLQSPNGPNHNYESKDGSCSRKRGYPQEMDRWFAVQEESHRVGHSVTASASGTNMSWASFESGRSLKTARTGDRDYFRSGSETQDTEGDEQETRGEAGRSNGRRGRAAAIHNESERRRRDRINQRMRTLQKLLPTASKADKVSILDDVIEHLKQLQAQVQFMSLRANLPQQMMIPQLPPPQSVLSIQHQQQQQQQQQQQQQQQQQFQMSLLATMARMGMGGGGNGYGGLVPPPPPPPMMVPPMGNRDCTNGSSATLSDPYSAFFAQTMNMDLYNKMAAAIYRQQSDQTTKVNIGMPSSSSNHEKRD

>AtbHLH073

MGDSDVGDRLPPPSSSDELSSFLRQILSRTPTAQPSSPPKSTNVSSAETFFPSVSGGAVSSVGYGVSETGQDKYAFEHKRSGAKQRNSLKRNIDAQFHNLSEKKRRSKINEKMKALQKLIPNSNKTDKASMLDEAIEYLKQLQLQVQTLAVMNGLGLNPMRLPQVPPPTHTRINETLEQDLNLETLLAAPHSLEPAKTSQGMCFSTATLL

>AtbHLH074

MGGESNEGGEMGFKHGDDESGGISRVGITSMPLYAKADPFFSSADWDPVVNAAAAGFSSSHYHPSMAMDNPGMSCFSHYQPGSVSGFAADMPASLLPFGDCGGGQIGHFLGSDKKGERLIRAGESSHEDHHQVSDDAVLGASPVGKRRLPEAESQWNKKAVEEFQEDPQRGNDQSQKKHKNDQSKETVNKESSQSEEAPKENYIHMRARRGQATNSHSLAERVRREKISERMRLLQELVPGCNKITGKAVMLDEIINYVQSLQQQVEFLSMKLATVNPEINIDIDRILAKDLLQSRDRNTPTLGLNPFAGFQGNIPNLSATTNPQYNPLPQTTLESELQNLYQMGFVSNPSTMSSFSPNGRLKPEL

>AtbHLH075

MARFEPYNYNNGHDPFFAHINQNPELINLDLPASTPSSFMLFSNGALVDANHNNSHFFPNLLHGNTRRKGNKEESGSKRRRKRSEEEEAMNGDETQKPKDVVHVRAKRGQATDSHSLAERVRREKINERLKCLQDLVPGCYKAMGMAVMLDVIIDYVRSLQNQIEFLSMKLSAASACYDLNSLDIEPTDIFQGGNIHSAAEMERILRESVGTQPPNFSSTLPF

>AtbHLH076

MSDKDEFAAKKKDLVNTPVDLYPPENPMLGPSPMMDSFRETLWHDGGFNVHTDADTSFRGNNNIDIPLEMGWNMAQFPADSGFIERAAKFSFFGCGEMMMNQQQSSLGVPDSTGLFLQDTQIPSGSKLDNGPLTDASKLVKERSINNVSEDSQSSGGNGHDDAKCGQTSSKGFSSKKRKRIGKDCEEEEDKKQKDEQSPTSNANKTNSEKQPSDSLKDGYIHMRARRGQATNSHSLAERVRREKISERMKFLQDLVPGCDKVTGKAVMLDEIINYVQSLQCQIEFLSMKLSAVNPVLDFNLESLLAKDALQSSAPTFPHNMSMLYPPVSYLSQTGFMQPNISSMSLLSGGLKRQETHGYESDHHNLVHMNHETGTAPDHEDTTADMKVEP

>AtbHLH077

MNMDKETEQTLNYLPLGQSDPFGNGNEGTIGDFLGRYCNNPQEISPLTLQSFSLNSQISENFPISGGIRFPPYPGQFGSDREFGSQPTTQESNKSSLLDPDSVSDRVHTTKSNSRKRKSIPSGNGKESPASSSLTASNSKVSGENGGSKGGKRSKQDVAGSSKNGVEKCDSKGDNKDDAKPPEAPKDYIHVRARRGQATDSHSLAERARREKISERMTLLQDLVPGCNRITGKAVMLDEIINYVQSLQRQVEFLSMKLATVNPRMEFNANASLSTEMIQPGESLTQSLYAMACSEQRLPSAYYSLGKNMPRFSDTQFPSNDGFVHTETPGFWENNDLQSIVQMGFGDILQQQSNNNNNNCSEPTLQMKLEP

>AtbHLH078

MDNELFMNTEFPPPPEMATHFEHQQSSSSAMMLNWALMDPNPHQDSSFLWEKSTEQQQQQSIFDSALSSLVSSPTPSNSNFSGGGGDGFLIRELIGKLGNIGNNNNNSGEIYGTPMSRSASCYATPMSSPPPPTNSNSQMMMNRTTPLTEFSADPGFAERAARFSCFGSRSFNGRTNTNLPINNGNNMVNNSGKLTRVSSTPALKALVSPEVTPGGEFSRKRKSVPKGKSKENPISTASPSPSFSKTAEKNGGKGGSKSSEEKGGKRRREEEDDEEEEGEGEGNKSNNTKPPEPPKDYIHVRARRGQATDSHSLAERVRREKIGERMKLLQDLVPGCNKVTGKALMLDEIINYVQSLQRQVEFLSMKLSSVNDTRLDFNVDALVSKDVMIPSSNNRLHEEGLQSKSSSHHHQQQLNIYNNNSQLLPNISSNNMMLQSPMNSLETSTLARSFTHLPTLTQFTDSISQYQMFSEEDLQSIVGMGVAENPNNESQHMKIEL

>AtbHLH079

MDPPLVNDSSFSAANPSSYTLSEIWPFPVNDAVRSGLRLAVNSGRVFTRSEHSGNKDVSAAEESTVTDLTAGWGSRKTRDLNSEDDSSKMVSSSSSGNELKESGDKKRKLCGSESGNGDGSMRPEGETSSGGGGSKATEQKNKPEPPKDYIHVRARRGQATDRHSLAERARREKISEKMTALQDIIPGCNKIIGKALVLDEIINYIQSLQRQVEFLSMKLEVVNSGASTGPTIGVFPSGDLGTLPIDVHRTIYEQQEANETRVSQPEWLHMQVDGNFNRTT

>AtbHLH080

MQSTHISGGSSGGGGGGGGEVSRSGLSRIRSAPATWIETLLEEDEEEGLKPNLCLTELLTGNNNSGGVITSRDDSFEFLSSVEQGLYNHHQGGGFHRQNSSPADFLSGSGSGTDGYFSNFGIPANYDYLSTNVDISPTKRSRDMETQFSSQLKEEQMSGGISGMMDMNMDKIFEDSVPCRVRAKRGCATHPRSIAERVRRTRISDRIRRLQELVPNMDKQTNTADMLEEAVEYVKALQSQIQELTEQQKRCKCKPKEEQ

>AtbHLH081

MQPTSVGSSGGGDDGGGRGGGGGLSRSGLSRIRSAPATWLEALLEEDEEESLKPNLGLTDLLTGNSNDLPTSRGSFEFPIPVEQGLYQQGGFHRQNSTPADFLSGSDGFIQSFGIQANYDYLSGNIDVSPGSKRSREMEALFSSPEFTSQMKGEQSSGQVPTGVSSMSDMNMENLMEDSVAFRVRAKRGCATHPRSIAERVRRTRISDRIRKLQELVPNMDKQTNTADMLEEAVEYVKVLQRQIQELTEEQKRCTCIPKEEQ

>AtbHLH082

MENGNGEGKGEFINQNNDFFLDSMSMLSSLPPCWDPSLPPPPPPPQSLFHALAVDAPFPDQFHHPQESGGPTMGSQEGLQPQGTVSTTSAPVVRQKPRVRARRGQATDPHSIAERLRRERIAERMKSLQELVPNTNKTDKASMLDEIIEYVRFLQLQVKVLSMSRLGGAGSVGPRLNGLSAEAGGRLNALTAPCNGLNGNGNATGSSNESLRSTEQRVAKLMEEDMGSAMQYLQGKGLCLMPISLATAISSSTTHSRGSLFNPISSAVAAEDSNVTATAVAAPEASSTMDDVSASKA

>AtbHLH083

MALVNDHPNETNYLSKQNSSSSEDLSSPGLDQPDAAYAGGGGGGGSASSSSTMNSDHQQHQGFVFYPSGEDHHNSLMDFNGSSFLNFDHHESFPPPAISCGGSSGGGGFSFLEGNNMSYGFTNWNHQHHMDIISPRSTETPQGQKDWLYSDSTVVTTGSRNESLSPKSAGNKRSHTGESTQPSKKLSSGVTGKTKPKPTTSPKDPQSLAAKNRRERISERLKILQELVPNGTKVDLVTMLEKAISYVKFLQVQVKVLATDEFWPAQGGKAPDISQVKDAIDAILSSSQRDRNSNLITN

>AtbHLH084

MEAMGEWSTGLGGIYTEEADFMNQLLASYEQPCGGSSSETTATLTAYHHQGSQWNGGFCFSQESSSYSGYCAAMPRQEEDNNGMEDATINTNLYLVGEETSECDATEYSGKSLLPLETVAENHDHSMLQPENSLTTTTDEKMFNQCESSKKRTRATTTDKNKRANKARRSQKCVEMSGENENSGEEEYTEKAAGKRKTKPLKPQKTCCSDDESNGGDTFLSKEDGEDSKALNLNGKTRASRGAATDPQSLYARKRRERINERLRILQHLVPNGTKVDISTMLEEAVQYVKFLQLQIKLLSSDDLWMYAPIAYNGMDIGLDLKLNALTR

>AtbHLH085

MEAMGEWSNNLGGMYTYATEEADFMNQLLASYDHPGTGSSSGAAASGDHQGLYWNLGSHHNHLSLVSEAGSFCFSQESSSYSAGNSGYYTVVPPTVEENQNETMDFGMEDVTINTNSYLVGEETSECDVEKYSSGKTLMPLETVVENHDDEESLLQSEISVTTTKSLTGSKKRSRATSTDKNKRARVNKRAQKNVEMSGDNNEGEEEEGETKLKKRKNGAMMSRQNSSTTFCTEEESNCADQDGGGEDSSSKEDDPSKALNLNGKTRASRGAATDPQSLYARKRRERINERLRILQNLVPNGTKVDISTMLEEAVHYVKFLQLQIKLLSSDDLWMYAPIAFNGMDIGLSSPR

>AtbHLH086

MSLINEHCNERNYISTPNSSEDLSSPQNCGLDEGASASSSSTINSDHQNNQGFVFYPSGETIEDHNSLMDFNASSFFTFDNHRSLISPVTNGGAFPVVDGNMSYSYDGWSHHQVDSISPRVIKTPNSFETTSSFGLTSNSMSKPATNHGNGDWLYSGSTIVNIGSRHESTSPKLAGNKRPFTGENTQLSKKPSSGTNGKIKPKATTSPKDPQSLAAKNRRERISERLKVLQELVPNGTKVDLVTMLEKAIGYVKFLQVQVKVLAADEFWPAQGGKAPDISQVKEAIDAILSSSQRDSNSTRETSIAE

>AtbHLH087

MEGLESVYAQAMYGMTRESKIMEHQGSDLIWGGNELMARELCSSSSYHHQLINPNLSSCFMSDLGVLGEIQQQQHVGNRASSIDPSSLDCLLSATSNSNNTSTEDDEGISVLFSDCQTLWSFGGVSSAESENREITTETTTTIKPKPLKRNRGGDGGTTETTTTTTKPKSLKRNRGDETGSHFSLVHPQDDSEKGGFKLIYDENQSKSKKPRTEKERGGSSNISFQHSTCLSDNVEPDAEAIAQMKEMIYRAAAFRPVNFGLEIVEKPKRKNVKISTDPQTVAARQRRERISEKIRVLQTLVPGGTKMDTASMLDEAANYLKFLRAQVKALENLRPKLDQTNLSFSSAPTSFPLFHPSFLPLQNPNQIHHPEC

>AtbHLH088

MDSDIMNMMMHQMEKLPEFCNPNSSFFSPDHNNTYPFLFNSTHYQSDHSMTNEPGFRYGSGLLTNPSSISPNTAYSSVFLDKRNNSNNNNNGTNMAAMREMIFRIAVMQPIHIDPEAVKPPKRRNVRISKDPQSVAARHRRERISERIRILQRLVPGGTKMDTASMLDEAIHYVKFLKKQVQSLEEQAVVTGGGGGGGGRVLIGGGGMTAASGGGGGGGVVMKGCGTVGTHQMVGNAQILR

>AtbHLH089

MGGGGMFEEIGCFDPNAPAEMTAESSFSPSEPPPTITVIGSNSNSNCSLEDLSAFHLSPQDSSLPASASAYAHQLHINATPNCDHQFQSSMHQTLQDPSYAQQSNHWDNGYQDFVNLGPNHTTPDLLSLLQLPRSSLPPFANPSIQDIIMTTSSSVAAYDPLFHLNFPLQPPNGSFMGVDQDQTETNQGVNLMYDEENNNLDDGLNRKGRGSKKRKIFPTERERRVHFKDRFGDLKNLIPNPTKNDRASIVGEAIDYIKELLRTIDEFKLLVEKKRVKQRNREGDDVVDENFKAQSEVVEQCLINKKNNALRCSWLKRKSKFTDVDVRIIDDEVTIKIVQKKKINCLLFVSKVVDQLELDLHHVAGAQIGEHHSFLFNAKISEGSSVYASAIADRVMEVLKKQYMEALSANNGYHCYSSD

>AtbHLH090

MMMMRGGERVKEFLRPFVDSRTWDLCVIWKLGDDPSRFIEWVGCCCSGCYIDKNIKLENSEEGGTGRKKKASFCRDDHNKHRIRTLACEALSRFPLFMPLYPGIHGEVVMSKSPKWLVNSGSKMEMFSTRVLVPVSDGLVELFAFDMRPFDESMVHLIMSRCTTFFEPFPEQRLQFRIIPRAEESMSSGVNLSVEGGGSSSVSNPSSETQNLFGNYPNASCVEILREEQTPCLIMNKEKDVVVQNANDSKANKKLLPTENFKSKNLHSERKRRERINQAMYGLRAVVPKITKLNKIGIFSDAVDYINELLVEKQKLEDELKGINEMECKEIAAEEQSAIADPEAERVSSKSNKRVKKNEVKIEVHETGERDFLIRVVQEHKQDGFKRLIEAVDLCELEIIDVNFTRLDLTVMTVLNVKANKDGIACGILRDLLLKMMITSI

>AtbHLH091

MYEESSCFDPNSMVDNNGGFCAAETTFTVSHQFQPPLGSTTNSFDDDLKLPTMDEFSVFPSVISLPNSETQNQNISNNNHLINQMIQESNWGVSEDNSNFFMNTSHPNTTTTPIPDLLSLLHLPRCSMSLPSSDIMAGSCFTYDPLFHLNLPPQPPLIPSNDYSGYLLGIDTNTTTQRDESNVGDENNNAQFDSGIIEFSKEIRRKGRGKRKNKPFTTERERRCHLNERYEALKLLIPSPSKGDRASILQDGIDYINELRRRVSELKYLVERKRCGGRHKNNEVDDNNNNKNLDDHGNEDDDDDDENMEKKPESDVIDQCSSNNSLRCSWLQRKSKVTEVDVRIVDDEVTIKVVQKKKINCLLLVSKVLDQLQLDLHHVAGGQIGEHYSFLFNTKIYEGSTIYASAIANRVIEVVDKHYMASLPNSNY

>AtbHLH092

MDNFFLGLSCQEENNFWDLIVADISGDRSVSVPIRSAFRSYMKDTELRMMSPKISSSKVNVKKRMVNLLRKNWEEKKNTVAPEKERSRRHMLKERTRREKQKQSYLALHSLLPFATKNDKNSIVEKAVDEIAKLQRLKKELVRRIKVIEEKSAKDGHDEMSETKVRVNLKEPLSGLDSMLEALHYLKSMGTKLKTVHANFSPQEFSATMTIETQIRGEEVEKRVERRLQETEWKLLFLPEASFYKDY

>AtbHLH093

MELSTQMNVFEELLVPTKQETTDNNINNLSFNGGFDHHHHQFFPNGYNIDYLCFNNEEEDENTLLYPSSFMDLISQPPPLLLHQPPPLQPLSPPLSSSATAGATFDYPFLEALQEIIDSSSSSPPLILQNGQEENFNNPMSYPSPLMESDQSKSFSVGYCGGETNKKKSKKLEGQPSKNLMAERRRRKRLNDRLSMLRSIVPKISKMDRTSILGDAIDYMKELLDKINKLQDEEQELGNSNNSHHSKLFGDLKDLNANEPLVRNSPKFEIDRRDEDTRVDICCSPKPGLLLSTVNTLETLGLEIEQCVISCFSDFSLQASCSEGAEQRDFITSEDIKQALFRNAGYGGSCL

>AtbHLH094

MPLEAVVYPQDPFGYLSNCKDFMFHDLYSQEEFVAQDTKNNIDKLGHEQSFVEQGKEDDHQWRDYHQYPLLIPSLGEELGLTAIDVESHPPPQHRRKRRRTRNCKNKEEIENQRMTHIAVERNRRKQMNEYLAVLRSLMPSSYAQRGDQASIVGGAINYVKELEHILQSMEPKRTRTHDPKGDKTSTSSLVGPFTDFFSFPQYSTKSSSDVPESSSSPAEIEVTVAESHANIKIMTKKKPRQLLKLITSLQSLRLTLLHLNVTTLHNSILYSISVRVEEGSQLNTVDDIATALNQTIRRIQEET

>AtbHLH095

MTNAQELGQEGFMWGISNSDDSGGGCKRIEKEPLPSHPSHPSPEIQTTTVKKGKKRTKRNDKNHEEESPDHEIHIWTERERRKKMRDMFSKLHALLPQLPPKADKSTIVDEAVSSIKSLEQTLQKLEMQKLEKLQYSSASTNTTPTTTFAYAPSSSSSPTALLTPISNHPIDATATDSYPRAAFLADQVSSSSAAAANLPYPCNDPIVNFDTWSSRNVVLTICGNEAFFNLCVPKHKPGVFTSVCYLFEKYNMEVLFANVSSNVFWSTYVIQAQVNPSCENQLLGNGLGVVDVFKQVSQELVLYFSSL

>AtbHLH096

MALEAVVYPQDPFSYISCKDFPFYDLYFQEEEDQDPQDTKNNIKLGQGQGHGFASNNYNGRTGDYSDDYNYNEEDLQWPRDLPYGSAVDTESQPPPSDVAAGGGRRKRRRTRSSKNKEEIENQRMTHIAVERNRRKQMNEYLAVLRSLMPPYYAQRGDQASIVGGAINYLKELEHHLQSMEPPVKTATEDTGAGHDQTKTTSASSSGPFSDFFAFPQYSNRPTSAAAAEGMAEIEVTMVESHASLKILAKKRPRQLLKLVSSIQSLRLTLLHLNVTTRDDSVLYSISVKVEEGSQLNTVEDIAAAVNQILRRIEEESSFS

>AtbHLH097

MDKDYSAPNFLGESSGGNDDNSSGMIDYMFNRNLQQQQKQSMPQQQQHQLSPSGFGATPFDKMNFSDVMQFADFGSKLALNQTRNQDDQETGIDPVYFLKFPVLNDKIEDHNQTQHLMPSHQTSQEGGECGGNIGNVFLEEKEDQDDDNDNNSVQLRFIGGEEEDRENKNVTKKEVKSKRKRARTSKTSEEVESQRMTHIAVERNRRKQMNEHLRVLRSLMPGSYVQRGDQASIIGGAIEFVRELEQLLQCLESQKRRRILGETGRDMTTTTTSSSSPITTVANQAQPLIITGNVTELEGGGGLREETAENKSCLADVEVKLLGFDAMIKILSRRRPGQLIKTIAALEDLHLSILHTNITTMEQTVLYSFNVKITSETRFTAEDIASSIQQIFSFIHANTNISGSSNLGNIVFT

>AtbHLH098

MQEIIPDFLEECEFVDTSLAGDDLFAILESLEGAGEISPTAASTPKDGTTSSKELVKDQDYENSSPKRKKQRLETRKEEDEEEEDGDGEAEEDNKQDGQQKMSHVTVERNRRKQMNEHLTVLRSLMPCFYVKRGDQASIIGGVVEYISELQQVLQSLEAKKQRKTYAEVLSPRVVPSPRPSPPVLSPRKPPLSPRINHHQIHHHLLLPPISPRTPQPTSPYRAIPPQLPLIPQPPLRSYSSLASCSSLGDPPPYSPASSSSSPSVSSNHESSVINELVANSKSALADVEVKFSGANVLLKTVSHKIPGQVMKIIAALEDLALEILQVNINTVDETMLNSFTIKIGIECQLSAEELAQQIQQTFC

>AtbHLH099

MMFQQDYPHGFSLVETSLSYEMLDYFQNIVVSNSEDVASQQNSISSSSYSSATLSCSITEQKSHLTEKLSPLRERYGCGDFLSRKRRRRSEKTIVDKENQRMNHIAVERNRRKQMNHFLSILKSMMPLSYSQPNDQASIIEGTISYLKKLEQRLQSLEAQLKATKLNQSPNIFSDFFMFPQYSTATATATATASSSSSSHHHHKRLEVVADVEVTMVERHANIKVLTKTQPRLLFKIINEFNSLGLSTLHLNLTTSKDMSLFTFSVKVEADCQLTPSGNEVANTVHEVVRRVHKER

>AtbHLH100

MCALVPPLYPNFGWPCGDHSFYETDDVSNTFLDFPLPDLTVTHENVSSENNRTLLDNPVVMKKLNHNASERERRKKINTMFSSLRSCLPPTNQTKKLSVSATVSQALKYIPELQEQVKKLMKKKEELSFQISGQRDLVYTDQNSKSEEGVTSYASTVSSTRLSETEVMVQISSLQTEKCSFGNVLSGVEEDGLVLVGASSSRSHGERLFYSMHLQIKNGQVNSEELGDRLLYLYEKCGHSFT

>AtbHLH101

MEYPWLQSQVHSFSPTLHFPSFLHPLDDSKSHNINLHHMSLSHSNNTNSNNNNYQEEDRGAVVLEKKLNHNASERDRRRKLNALYSSLRALLPLSDQKRKLSIPMTVARVVKYIPEQKQELQRLSRRKEELLKRISRKTHQEQLRNKAMMDSIDSSSSQRIAANWLTDTEIAVQIATSKWTSVSDMLLRLEENGLNVISVSSSVSSTARIFYTLHLQMRGDCKVRLEELINGMLLGLRQS

>AtbHLH102

MRTGKGNQEEEDYGEEDFNSKREGPSSNTTVHSNRDSKENDKASAIRSKHSVTEQRRRSKINERFQILRELIPNSEQKRDTASFLLEVIDYVQYLQEKVQKYEGSYPGWSQEPTKLTPWRNNHWRVQSLGNHPVAINNGSGPGIPFPGKFEDNTVTSTPAIIAEPQIPIESDKARAITGISIESQPELDDKGLPPLQPILPMVQGEQANECPATSDGLGQSNDLVIEGGTISISSAYSHELLSSLTQALQNAGIDLSQAKLSVQIDLGKRANQGLTHEEPSSKNPLSYDTQGRDSSVEEESEHSHKRMKTL

>AtbHLH103

MTEEFDTTGVCTGTWWSSSNGMFSGCSLPRSAEIVVDFGEIEWQNIDTLDAKTYNENYLSTSTFLGNANLDTTSQIYVSSPSNIHEEERYNQINSFLEGLFDSSEQLLVPNCPKPELFESFHFFDDVFPNESRMISVFDHQKPKEDMQACKSLTTCKRASEKSGELEDIESSQPLKRPRLETPSHFPSFKVRKEKLGDRITALQQLVSPFGKTDTASVLHDAIDYIKFLQEQITEKVSTSPHLNSIGSGEQKQWSDKSSNNTHNQNCSPRQDLRSRGLCLMPISSTFSTPPQHLDTSSLWN

>AtbHLH104

MYPSLDDDFVSDLFCFDQSNGAELDDYTQFGVNLQTDQEDTFPDFVSYGVNLQQEPDEVFSIGASQLDLSSYNGVLSLEPEQVGQQDCEVVQEEEVEINSGSSGGAVKEEQEHLDDDCSRKRARTGSCSRGGGTKACRERLRREKLNERFMDLSSVLEPGRTPKTDKPAILDDAIRILNQLRDEALKLEETNQKLLEEIKSLKAEKNELREEKLVLKADKEKTEQQLKSMTAPSSGFIPHIPAAFNHNKMAVYPSYGYMPMWHYMPQSVRDTSRDQELRPPAA

>AtbHLH105

MVSPENANWICDLIDADYGSFTIQGPGFSWPVQQPIGVSSNSSAGVDGSAGNSEASKEPGSKKRGRCESSSATSSKACREKQRRDRLNDKFMELGAILEPGNPPKTDKAAILVDAVRMVTQLRGEAQKLKDSNSSLQDKIKELKTEKNELRDEKQRLKTEKEKLEQQLKAMNAPQPSFFPAPPMMPTAFASAQGQAPGNKMVPIISYPGVAMWQFMPPASVDTSQDHVLRPPVA

>AtbHLH106

MQPETSDQMLYSFLAGNEVGGGGYCVSGDYMTTMQSLCGSSSSTSSYYPLAISGIGETMAQDRALAALRNHKEAERRRRERINSHLNKLRNVLSCNSKTDKATLLAKVVQRVRELKQQTLETSDSDQTLLPSETDEISVLHFGDYSNDGHIIFKASLCCEDRSDLLPDLMEILKSLNMKTLRAEMVTIGGRTRSVLVVAADKEMHGVESVHFLQNALKSLLERSSKSLMERSSGGGGGERSKRRRALDHIIMV

>AtbHLH107

MQPEVSDQIFYAFLTGGLCASSTSTTVTSSSDPFATVYEDKALASLRNHKEAERKRRARINSHLNKLRKLLSCNSKTDKSTLLAKVVQRVKELKQQTLEITDETIPSETDEISVLNIEDCSRGDDRRIIFKVSFCCEDRPELLKDLMETLKSLQMETLFADMTTVGGRTRNVLVVAADKEHHGVQSVNFLQNALKSLLERSSKSVMVGHGGGGGEERLKRRRALDHIIMV

>AtbHLH108

MNKDEVFLRQWFEILYSLTNPEANSDLRRINNEKGVEKVGQKRSAESRREGKKKRVKTQCVIKSSDKSDHDTLLKKKRRERIRRQLETLKEITPNCPQSDINAILDCVIEYTNNLRLAHYKGSQGICDDWRLFTEAGAVLYYIDT

>AtbHLH109

MERNNRNEGTHEEEQCSLSDIIYSFCSENHSELNPLQEIFGVTKNNDHEKHDEEPDEESYRMAKRQRSMEYRMMMEKKRRKEIKDKVDILQGLMPNHCTKPDLASKLENIIEYIKSLKYQVDVMSMAYTTTPVYTPPFYAAAQAPCMSPWGYYTPGVPMMPQQNMTYIPQYPQVYGTVPPNQTQP

>AtbHLH110

MDSANLHQLQDQLQLVGSSSSSSSLDNNSDPSCYGASSAHQWSPGGISLNSVSLSHNYNNEMLNTRAHNNNNNNNTSECMSLSSIHNHSLIQQQDFPLQWPHDQSSYQHHEGLLKIKEELSSSTISDHQEGISKFTDMLNSPVITNYLKINEHKDYTEKLLLKSMSSGFPINGDYGSSLPSSSSSSSPSSQSHRGNFSQIYPSVNISSLSESRKMSMDDMSNISRPFDINMQVFDGRLFEGNVLVPPFNAQEISSLGMSRGSLPSFGLPFHHHLQQTLPHLSSSPTHQMEMFSNEPQTSEGKRHNFLMATKAGENASKKPRVESRSSCPPFKVRKEKLGDRIAALQQLVSPFGKTDTASVLMEAIGYIKFLQSQIETLSVPYMRASRNRPGKASQLVSQSQEGDEEETRDLRSRGLCLVPLSCMTYVTGDGGDGGGGVGTGFWPTPPGFGGGT

>AtbHLH112

MAEEFKATASICGGGGGAWWNSPRSVMSPSDHFLSPCFGAAITSNDFSSQENHLKSRMTCTDNNNIVFGQREADSDSGGSTVTMDSTLQMMGLGFSSNCSSDWNQTILQEDLNSSFIRSSQDQDHGQGFLSTTTSPYILNPACSSSPSTSSSSSLIRTFYDPEPSPYNFVSTTSGSINDPQLSWANKTNPHHQVAYGLINSFSNNANSRPFWNSSSTTNLNNTTPSNFVTTPQIISTRLEDKTKNLKTRAQSESLKRAKDNESAAKKPRVTTPSPLPTFKVRKENLRDQITSLQQLVSPFGKTDTASVLQEAIEYIKFLHDQVTVLSTPYMKQGASNQQQQQISGKSKSQDENENHELRGHGLCLVPISSTFPVANETTADFWTPTFGGNNFR

>AtbHLH113

MGDTAEDQDDRAMMEAEGVTSFSELLMFSDGVLSSSSDHQPEGNVGDGGEDSLGFVFSGKTGSRMLCFSGGYQNDDESLFLEPSVPTSGVSDLDPSCIKIDCRNSNDACTVDKSTKSSTKKRTGTGNGQESDQNRKPGKKGKRNQEKSSVGIAKVRKERLGERIAALQQLVSPYGKTDAASVLHEAMGYIKFLQDQIQVLCSPYLINHSLDGGVVTGDVMAAMKAKDLRSRGLCLVPVSSTVHVENSNGADFWSPATMGHTTSPSLPQGF

>AtbHLH114

MNMHNSFFEGLLIDPNDQLLPDPWSKSTIPNAKSELLENFPFLDNMFLVDSEAESLLDHEIRNHKSSKEQITQDYKNLTSKRSEELEENSDEYSPRLLKRPRLETLSPLPSFKVRKEKLGDRITALQQLVSPFGKTDTASVLNEAVEYIKFLQEQVTVLSNPEQNTIGSVQQQQCSNKKSINTQGEVEEDECSPRRYVDLSSRGLCLMPISASYPVAAAAASAAEMNVHLVSGIFHSL

>AtbHLH115

MVSPENTNWLSDYPLIEGAFSDQNPTFPWQIDGSATVSVEVDGFLCDADVIKEPSSRKRIKTESCTGSNSKACREKQRRDRLNDKFTELSSVLEPGRTPKTDKVAIINDAIRMVNQARDEAQKLKDLNSSLQEKIKELKDEKNELRDEKQKLKVEKERIDQQLKAIKTQPQPQPCFLPNPQTLSQAQAPGSKLVPFTTYPGFAMWQFMPPAAVDTSQDHVLRPPVA

>AtbHLH116

MGLDGNNGGGVWLNGGGGEREENEEGSWGRNQEDGSSQFKPMLEGDWFSSNQPHPQDLQMLQNQPDFRYFGGFPFNPNDNLLLQHSIDSSSSCSPSQAFSLDPSQQNQFLSTNNNKGCLLNVPSSANPFDNAFEFGSESGFLNQIHAPISMGFGSLTQLGNRDLSSVPDFLSARSLLAPESNNNNTMLCGGFTAPLELEGFGSPANGGFVGNRAKVLKPLEVLASSGAQPTLFQKRAAMRQSSGSKMGNSESSGMRRFSDDGDMDETGIEVSGLNYESDEINESGKAAESVQIGGGGKGKKKGMPAKNLMAERRRRKKLNDRLYMLRSVVPKISKMDRASILGDAIDYLKELLQRINDLHNELESTPPGSLPPTSSSFHPLTPTPQTLSCRVKEELCPSSLPSPKGQQARVEVRLREGRAVNIHMFCGRRPGLLLATMKALDNLGLDVQQAVISCFNGFALDVFRAEQCQEGQEILPDQIKAVLFDTAGYAGMI

>AtbHLH117

METPAYDFDSLTDLPPLPPSDFTPSNAFTFPDHNLDFSFLDSTLSLLNRHHLSESTRLEQIFYDSTHTQLFHNDDTTTTTTPFLHLPDLKSIDAVEEPTTMKLFPSLSPPLPAAKRQKLNSTSSSTTSGSPTASNDGGIITKRRKISDKIRSLEKLMPWERKMNLAMTLEESHKYIKFLQSQIASLRWMPLESVYNTAGEVGETDLLKSLTRQQILQVLANSPGSRNVLSSRGVCVFSYEQLLSLKTMSRNL

>AtbHLH118

MEKLVHKEIEKRRRQEMASLYASLRSLLPLEFIQGKRSTSDQVKGAVNYIDYLQRNIKDINSKRDDLVLLSGRSFRSSNEQEWNEISNHVVIRPCLVGIEIVLSILQTPFSSVLQVLREHGLYVLGYICSSVNDRLIHTLQAEVNDLALIDLADLKDTLTLMK

>AtbHLH119

MGEDDIVELLWNGQVVRTSQPQRPSSGKPSPTPPILRGSGSGSGEENAPLPLPLLQPPRPLHHQNLFIREEEMSSWLHYSYTGVTSTPATHPQSSVSLPPPPPIAPSEDDVVELLWKSGQVVQSIQTQRPIPPPIFRGSGSGGGEETVLPLPPLHPSHQNIFIQEDEMASWLYHPLRQDYFSSGVASTSATRPQSSASLAPTPPPPSVPYGQIPVERRTENFMNFLRLRGNIFSGGRVEAGPVVIESTQIGSSATPSSSAAESCVIPATHGTESRAAAITGVSRTFAVPGLGRRGKEVATETAGTSYSGVNKAETERVQIQPERETKITEDKKREETIAEIQGTEEAHGSTSRKRSRAADMHNLSERRRRERINERMKTLQELLPRCRKTDKVSMLEDVIEYVKSLQLQIQMMSMGHGMMPPMMHEGNTQQFMPHMAMGMKGMNRPPPFVPFPGKTFPRPGHMAGVGPSYPALRYPFPDTQASDLSRVHVPSLHSNPVPNQPRFPAYINPYSQFVGLHQMQQPPLPLQGQPTSQPSFSHASTSK

>AtbHLH120

MNPSNNPKKTRHQSHMPQERDETKKEKKLLHRNIERQRRQEMAILFASLRSQLPLKYIKALSSQGKRAMSDHVNGAVSFIKDTQTRIKDLSARRDELKREIGDPTSLTGSGSGSGSSRSEPASVMVQPCVSGFEVVVSSLASGLEAWPLSRVLEVLHGQGLEVISSLTARVNERLMYTIQVEVNSFDCFDLAWLQQKLIEQLVLSTTRH

>AtbHLH121

MDVSARKSQKAGREKLRREKLNEHFVELGNVLDPERPKNDKATILTDTVQLLKELTSEVNKLKSEYTALTDESRELTQEKNDLREEKTSLKSDIENLNLQYQQRLRSMSPWGAAMDHTVMMAPPPSFPYPMPIAMPPGSIPMHPSMPSYTYFGNQNPSMIPAPCPTYMPYMPPNTVVEQQSVHIPQNPGNRSREPRAKVSRESRSEKAEDSNEVATQLELKTPGSTSDKDTLQRPEKTKRCKRNNNNNSIEESSHSSKCSSSPSVRDHSSSSSVAGGQKPDDAK

>AtbHLH122

MESEFQQHHFLLHDHQHQRPRNSGLIRYQSAPSSYFSSFGESIEEFLDRPTSPETERILSGFLQTTDTSDNVDSFLHHTFNSDGTEKKPPEVKTEDEDAEIPVTATATAMEVVVSGDGEISVNPEVSIGYVASVSRNKRPREKDDRTPVNNLARHNSSPAGLFSSIDVETAYAAVMKSMGGFGGSNVMSTSNTEASSLTPRSKLLPPTSRAMSPISEVDVKPGFSSRLPPRTLSGGFNRSFGNEGSASSKLTALARTQSGGLDQYKTKDEDSASRRPPLAHHMSLPKSLSDIEQLLSDSIPCKIRAKRGCATHPRSIAERVRRTKISERMRKLQDLVPNMDTQTNTADMLDLAVQYIKDLQEQVKALEESRARCRCSSA

>AtbHLH123

MGDHHDFINSGSWWKVSSSSSPSSSSSMRASSIESGGSAVFHDKLHHHSLATDHHLQMIGLGLSSQSPVDQWNQSLLRGDSKAETSFGVMLQENLNLDATSNANANTTSSTSSYQLQESDSSHHHQALWRDPQSDFKPQILTSGGNRGFFLDHQFSPHGSSSTDSSTVTCQGFAVDNSSNAMYAATTTTPNSSSGMFHHQQAGGFGSSDQQPSRNHQQSSLGYSQFGSSTGNYDQMASALPSTWFLRSSPPPKPHSPLRFSNNATFWNPAAAGNAGAPPPHDASSNFFPALQPPQIHPQSFDEQPKNISEIRDSSSNEVKRGGNDHQPAAKRAKSEAASPSPAFKRKEKMGDRIAALQQLVSPFGKTDAASVLSEAIEYIKFLHQQVSALSNPYMKSGASLQHQQSDHSTELEVSEEPDLRSRGLCLVPVSSTFPVTHDTTVDFWTPTFGGTFR

>AtbHLH124

MEAKPLASSSSEPNMISPSSNIKPKLKDEDYMELVCENGQILAKIRRPKNNGSFQKQRRQSLLDLYETEYSEGFKKNIKILGDTQVVPVSQSKPQQDKETNEQMNNNKKKLKSSKIEFERNVSKSNKCVESSTLIDVSAKGPKNVEVTTAPPDEQSAAVGRSTELYFASSSKFSRGTSRDLSCCSLKRKYGDIEEEESTYLSNNSDDESDDAKTQVHARTRKPVTKRKRSTEVHKLYERKRRDEFNKKMRALQDLLPNCYKDDKASLLDEAIKYMRTLQLQVQMMSMGNGLIRPPTMLPMGHYSPMGLGMHMGAAATPTSIPQFLPMNVQATGFPGMNNAPPQMLSFLNHPSGLIPNTPIFSPLENCSQPFVVPSCVSQTQATSFTQFPKSASASNLEDAMQYRGSNGFSYYRSPN

>AtbHLH125

MDCVPSLFMPDSTYEDGLLFSDSFLLSPFISYQNNDVFHSITNKIGGSNKKRSLCDITYGANEANKNDDDRESKKMKHRDIERQRRQEVSSLFKRLRTLLPFQYIQGKRSTSDHIVQAVNYIKDLQIKIKELNEKRNRVKKVISATTTTHSAIEECTSSLSSSAASTLSSSCSCVGDKHITVVVTPCLVGVEIIISCCLGRNKSCLSSVLQMLAQEQRFSVVSCLSARRQQRFMHTIVSQVEDGKQINILELKDKIMTM

>AtbHLH126

MDPYKNLNPKGYQRQRPFSSAGESGGSGGSGTAHETDDNKKKKKLLHRDIERQRRQEMATLFATLRTHLPLKYIKGKRAVSDHVNGAVNFIKDTEARIKELSARRDELSRETGQGYKSNPDPGKTGSDVGKSEPATVMVQPHVSGLEVVVSSNSSGPEALPLSKVLETIQEKGLEVMSSFTTRVNDRLMHTIQVEVNSFGCIDLLWLQQKLVEDLILSTGY

>AtbHLH127

MMIISSQILLLFGFKLFFETRGEDDIVELLCKIGQTQIPSSDPLPILRGSGSGGREENTPLPPPLPHQNLFIQEDEMSSWPHHPLRQDYLCSELYASTPAPHPQSSVSLAPPPPKPPSSAPYGQIIAPRSAPRIQGTEEARGSTSRKRSRAAEMHNLAERRRREKINERMKTLQQLIPRCNKSTKVSMLEDVIEYVKSLEMQINQFMPHMAMGMNQPPAYIPFPSQAHMAGVGPSYPPPRYPFPNIQTFDPSRVWLQSPQPNPVSNQPQMNPYGQFVGHHQMQQSLPPPLQVILSQYPLCLFLCSNK

>AtbHLH128

MYQSSSSTSSSSQRSSLPGGGGLIRYGSAPGSFLNSVVDEVIGGGSSNARDFTGYQPSSDNFIGNFFTGAADSSSLRSDSTTCGVNNSSDGQKQLGNNNNNNSNKDIFLDRSYGGFNEISQQHKSNDIGGGNSSGSYSLARQRSSPADFFTYLASDKNNFSLNQPTSDYSPQGGSNGGRGHSRLKSQLSFTNHDSLARINEVNETPVHDGSGHSFSAASFGAATTDSWDDGSGSIGFTVTRPSKRSKDMDSGLFSQYSLPSDTSMNYMDNFMQLPEDSVPCKIRAKRGCATHPRSIAERERRTRISGKLKKLQDLVPNMDKQTSYSDMLDLAVQHIKGLQHQLQNLKKDQENCTCGCSEKPS

>AtbHLH129

MYPPNSSKSTAHDGGGDADTNQYDSAAGATRDFSSLGPQTHHHPPPQRQQQHQQNPNLVGHYLPGEPSSIGFDSNASSSSSLFRHRSSPAGFYDQHLPTDPNGTGFSLGRPNGGYGGGGEQGPSRLKSELRFSSGSSSHQEHNSLPRISEVEAAAAARNGVASSSMSFGNNRTNNWDNSSSHISFTIDQPGKRSKNSDFFTLETQYSMPQTTLEMATMENLMNIPEDSVPCRARAKRGFATHPRSIAERERRTRISGKLKKLQELVPNMDKQTSYADMLDLAVEHIKGLQHQVEVRP

>AtbHLH130

MDSNNHLYDPNPTGSGLLRFRSAPSSVLAAFVDDDKIGFDSDRLLSRFVTSNGVNGDLGSPKFEDKSPVSLTNTSVSYAATLPPPPQLEPSSFLGLPPHYPRQSKGIMNSVGLDQFLGINNHHTKPVESNLLRQSSSPAGMFTNLSDQNGYGSMRNLMNYEEDEESPSNSNGLRRHCSLSSRPPSSLGMLSQIPEIAPETNFPYSHWNDPSSFIDNLSSLKREAEDDGKLFLGAQNGESGNRMQLLSHHLSLPKSSSTASDMVSVDKYLQLQDSVPCKIRAKRGCATHPRSIAERVRRTRISERMRKLQELVPNMDKQTNTSDMLDLAVDYIKDLQRQYKILNDNRANCKCMNKEKKSI

>AtbHLH132

MELVFENGQILAKGQRSNVSLHNQRTKSIMDLYEAEYNEDFMKSIIHGGGGAITNLGDTQVVPQSHVAAAHETNMLESNKHVDDSETLKASSSKRMMVDYHNRKKIKFIPPDEQSVVADRSFKLGFDTSSVGFTEDSEGSMYLSSSLDDESDDARPQVPARTRKALVKRKRNAEAYNSPERNQRNDINKKMRTLQNLLPNSHKDDNESMLDEAINYMTNLQLQVQMMTMGNRFVTPSMMMPLGPNYSQMGLAMGVGMQMGEQQFLPAHVLGAGLPGINDSADMLRFLNHPGLMPMQNSAPFIPTENCSPQSVPPSCAAFPNQIPNPNSLSNLDGATLHKKSRKTNR

>AtbHLH134

MSSSRRSRQASSSSRISDDQITDLISKLRQSIPEIRQNRRSNTVSASKVLQETCNYIRNLNKEADDLSDRLTQLLESIDPNSPQAAVIRSLING

>AtbHLH135

MSGRRSRSRQSSGTSRISEDQINDLIIKLQQLLPELRDSRRSDKVSAARVLQDTCNYIRNLHREVDDLSERLSELLANSDTAQAALIRSLLTQ

>AtbHLH136

MSNRRSRQSSSAPRISDNQMIDLVSKLRQILPEIGQRRRSDKASASKVLQETCNYIRNLNREVDNLSERLSQLLESVDEDSPEAAVIRSLLM

>AtbHLH137

MATFSYFQNYPHSLLDPLLFPTPHSSINLTSFIDQNHLYPLPNISTVEDISFLEYNVDKTENSGSEKLANTTKTATTGSSSCDQLSHGPSAITNTGKTRGRKARNSNNSKEGVEGRKSKKQKRGSKEEPPTDYIHVRARRGQATDSHSLAERVRREKISERMRTLQNLVPGCDKVTGKALMLDEIINYVQTLQTQVEFLSMKLTSISPVVYDFGSDLDGLILQSEMGSPEVGTSFTNAMPTTTPIFPSLLDNSVVPTHAQVQEEGEERENFVDRSGFNNNNFCSFP

>AtbHLH138

MERYTKKNERFKAEEGKGSKKSRTFLTERERRALFNDRFFDLKNLIPNPTKGGEASIVQDGIVYINELQRLVSELKYLVEKKKCGARHNNIEVDNKNTIYGTSKIEHPFSKNKNTFNCLIRTLRFVHHF

>AtbHLH139

MENEAFVDGELESLLGMFNFDQCSSNESSFCNAPNETDVFSSDDFFPFGTILQSNYAAVLDGSNHQTNRNVDSRQDLLKPRKKQKLSSESNLVTEPKTAWRDGQSLSSYNSSDDEKALGLVSNTSKSLKRKAKANRGIASDPQSLYARKRRERINDRLKTLQSLVPNGTKVDISTMLEDAVHYVKFLQLQIKLLSSEDLWMYAPLAHNGLNMGLHHNLLSRLI

>AtbHLH140

MDDFNLRSENPNSSSTTSSSSSSFHRHKSETGNTKRSRSTSTLSTDPQSVAARDRRHRISDRFKILQSMVPGGAKMDTVSMLDEAISYVKFLKAQIWYHQNMLLFINDHETTSSCTYSPGAGEFGPKLFGYDDDYAPIMDTYSQGVPLTVADSKYTPWFGSVDDEQEHVTYFKYRRATRHALRGHCNCIIGETEEFADQREKMEVQIEESGKNQTSPESIEADKAKQIVVLLIGPPGSGKSTFCDTAMRSSHRPWSRICQDIVNNGKAGTKAQCLKMATDSLREGKSVFIDRCNLDREQRSEFIKLGGPEFEVHAVVLELPAQVCISRSVKRTGHEGNLQGGRAAAVVNKMLQSKELPKVNEGFSRIMFCYSDADVDNAVNMYNKLGPMDTLPSGCFGEKKLDTKSQPGIMKFFKKVSALPASSSNEATNTTRKADEMTANVRVSPVKLGSADIVPTLAFPSISTADFQFDLEKASDIIVEKAEEFLSKLGTARLVLVDLSRGSKILSLVKAKASQKNIDSAKFFTFVGDITKLRSEGGLHCNVIANATNWRLKPGGGGVNAAIFKAAGPDLETATRVRANTLLPGKAVVVPLPSTCPLHNAEGITHVIHVLGPNMNPNRPDNLNNDYTKGCKTLREAYTSLFEGFLSVVQDQSKLPKRSSQTAVSDSGEDIKEDSERNKKYKGSQDKAVTNNLESESLEDTRGSGKKMSKGWNTWALALHSIAMHPERHENVVLEYLDNIVVINDQYPKARKHVLVLARQESLDGLEDVRKENLQLLQEMHNVGLKWVDRFQNEDASLIFRLGYHSVPSMRQLHLHVISQDFNSDSLKNKKHWNSFTTSFFRDSVDVLEEVNSQGKANVASEDLLKGELRCNRCRSAHPNIPKLKSHVRSCHSQFPDHLLQNNRLVARAET

>AtbHLH141

MNSHDIDDQLEADVYSNLPSRNDSSTGRRNRNSCRSKHSETEQRRRSKINERFQSLMDIIPQNQNDQKRDKASFLLEVIEYIHFLQEKVHMYEDSHQMWYQSPTKLIPWRNSHGSVAEENDHPQIVKSFSSNDKVAASSGFLLDTYNSVNPDIDSAVSTKIPEHSPVSAVSSYLRTEPSLQFVQHDFWQPKTSCGTINCFTNELLTSDEKTSASLSTVCSQRVLNTLTEALKSSGVNMSETMISVQLSLRKREDREYSVAAFASEDNGNSIADEEGDSPTETRSFCNDIDHSQKRIRR

>AtbHLH142

MPLDKRQRDLPLGLSPQACFKDIVGRSVLPRIPLPELGKLYAAKLQARCLQPPPFQSLLCSHDKESYGKRFSRSDMRSWCAAATTTTTPLGALESSQKRLLIFDQSGDQTRLLQCPFPLRFPSHAAAEPVKLSELQGIEKAFKEDGEEFHKSDGTESEMHEDTEEINALLYSDDDYDDDCESDDEVMSTGHSPYPNEGVCNKRELEEIDGPCKRQKLLDKVNNISDLSSLVGTESSTQLNGSSFLKDKKLPESKTISTKEDTGSGLSNEQSKKDKIRTALKILESVVPGAKGNEALLLLDEAIDYLKLLKRDLISTEVKNQSSTTHKSPILLLKETTWGTRNLQTDKA

>AtbHLH143

MPLDTKQQKWLPLGLNPQACVQDKATEYFRPGIPFPELGKVYAAEHQFRYLQPPFQALLSRYDQQSCGKQVSCLNGRSSNGAAPEGALKSSRKRFIVFDQSGEQTRLLQCGFPLRFPSSMDAERGNILGALHPEKGFSKDHAIQEKILQHEDHENGEEDSEMHEDTEEINALLYSDDDDNDDWESDDEVMSTGHSPFTVEQQACNITTEELDETESTVDGPLLKRQKLLDHSYRDSSPSLVGTTKVKGLSDENLPESNISSKQETGSGLSDEQSRKDKIHTALRILESVVPGAKGKEALLLLDEAIDYLKLLKQSLNSSKGLNNHW

>AtbHLH144

MQNNQFPHFSDEVGDRNMHNPYASGSSYDALFPPCAKLPYHGVELQPSAVCPKNFVIFDQTYDRSQVMYHPELTHKLMNTPSLNNLASTFQNEYVGGSYGNYGNYEQEVSSSYQEDPNEIDALLSADEDYEENDDNEGEEDGGDSEEVSTARTSSRDYGNTTAESCCSSYGYNNNNNNNSRKQSLSGSASSSNNDGKGRKKMKKMMGVLRRIVPGGEQMNTACVLDEAVQYLKSLKIEAQKLGVGHFSNQS

>AtbHLH145

MGQDRGFGFPTQRLCSLSSLALSHLGKQDLNLVSKTCGDTTDMFSTRGSYQVSTQVSQSYFDGYCGWVHGSSHLQQQFLPPQNQCMKQVPLQVDGVISKAEEQCSQKRFLVFDQSGDQTTLLLASDIRKSFETLKQHACPDMKEELQRSNKDLFVCHGMQGNSEPDLKEDSEELNALLYSEDESGYCSEEDEVTSADHSPSIVVSGREDQKTFLGSYGQPLNAKKRKILETSNESMRDAESSCGSCDNTRISFLKRSKLSSNKIGEEKIFETVSLLRSVVPGEELVDPILVIDRAIDYLKSLKMEAKNREA

>AtbHLH146

MERQIINRKKRVFSLEPNKNPSAVFTRKYTSHLVPALKKLNMNKNSSKQTVKHEVDMALALSAQEFAWSRFLLQKLSSSSNPTTTTSSSSDGIRILERPDKEGGNEEGGIEERLRELKKLLPGGEEMNVEEMLSEIGNYIKCLELQTIALKSIVQDST

>AtbHLH147

MESISPVSNQLLQPTTTSSNSDRSRRKRKKKSSPSSVEKSPSPSISLEKWRSEKQQQIYSTKLVHALRELRISQQPSSSSSSSIPRGGRAVREVADRALAVAARGKTLWSRAILSKAVKLKFRKHKRQRISNPTTTTLTTGSIRSKKQRATVLRLKAKGLPAVQRKVKVLSRLVPGCRKQSLPVVLEETTDYIAAMEMQIRTMTAILSAVSSSPPPPTPGHEGGQTHMLG

>AtbHLH148

MASLISDIEPPTSTTSDLVRRKKRSSASSAASSRSSASSVSGEIHARWRSEKQQRIYSAKLFQALQQVRLNSSASTSSSPTAQKRGKAVREAADRALAVSARGRTLWSRAILANRIKLKFRKQRRPRATMAIPAMTTVVSSSSNRSRKRRVSVLRLNKKSIPDVNRKVRVLGRLVPGCGKQSVPVILEEATDYIQALEMQVRAMNSLVQLLSSYGSAPPPI

>AtbHLH149

MVESLFPSIENTGESSRRKKPRISETAEAEIEARRVNEESLKRWKTNRVQQIYACKLVEALRRVRQRSSTTSNNETDKLVSGAAREIRDTADRVLAASARGTTRWSRAILASRVRAKLKKHRKAKKSTGNCKSRKGLTETNRIKLPAVERKLKILGRLVPGCRKVSVPNLLDEATDYIAALEMQVRAMEALAELLTAAAPRTTLTGT

>AtbHLH150

MSSEQGNGSNPSTSPEVEGTKTIPFRRRLQRGQRVFAPKLMEALRRSRVSSEEAPVRHLSRRWRATTAQKVYSLKLYDALQRSRRSATVRDTADKVLATTARGATRWSRAILVSRFGTSLRRRRNTKPASALAAAIRGSGGSGRRRKLSAVGNRVRVLGGLVPGCRRTALPELLDETADYIAALEMQVRAMTALSKILSELQPSTNLGSAL

>AtbHLH151

MGVTLEGQRKESIWVLMRRQRARRALVKKIMIRPRKSVEASRRPCRAIHRRVKTLKELVPNTKTSEGLDGLFRQTADYILALEMKVKVMQTMVQVLTETNCV

>AtbHLH153

MEFSRDAGMMMENKRNVCSLGESSIKRHKSDLSFSSKERKDKVGERISALQQIVSPYGKTDTASVLLDAMHYIEFLHEQVKVLSAPYLQTVPDATQEELEQYSLRNRGLCLVPMENTVGVAQSNGADIWAPVKTPLSPAFSVTSQSPFR

>AtbHLH154

MEYSRDSAEMMMETKRNVYSLEDNKIKRHKSSDLSFSSKERKDKLAERISALQQLVSPYGKTDTASVLLEGMQYIQFLQEQVKVLSAPYLQATPSTTEEEVEEYSLRSKGLCLVPLEYTSEVAQTNGADIWAPVKTPTSSHAFNLSSSNSPFQ

>AtbHLH155

MGSTSQEILKSFCFNTDWDYAVFWQLNHRGSRMVLTLEDAYYDHHGTNMHGAHDPLGLAVAKMSYHVYSLGEGIVGQVAVSGEHQWVFPENYNNCNSAFEFHNVWESQISAGIKTILVVAVGPCGVVQLGSLCKVNEDVNFVNHIRHLFLALRDPLADHAANLRQCNMNNSLCLPKMPSEGLHAEAFPDCSGEVDKAMDVEESNILTQYKTRRSDSMPYNTPSSCLVMEKAAQVVGGREVVQGSTCGSYSGVTFGFPVDLVGAKHENQVGTNIIRDAPHVGMTSGCKDSRDLDPNLHLYMKNHVLNDTSTSALAIEAERLITSQSYPRLDSTFQATSRTDKESSYHNEVFQLSENQGNKYIKETERMLGRNCESSQFDALISSGYTFAGSELLEALGSAFKQTNTGQEELLKSEHGSTMRPTDDMSHSQLTFDPGPENLLDAVVANVCQRDGNARDDMMSSRSVQSLLTNMELAEPSGQKKHNIVNPINSAMNQPPMAEVDTQQNSSDICGAFSSIGFSSTYPSSSSDQFQTSLDIPKKNKKRAKPGESSRPRPRDRQLIQDRIKELRELVPNGSKCSIDSLLERTIKHMLFLQNVTKHAEKLSKSANEKMQQKETGMQGSSCAVEVGGHLQVSSIIVENLNKQGMVLIEMLCEECGHFLEIANVIRSLDLVILRGFTETQGEKTWICFVTESQNSKVMQRMDILWSLVQIFQPKANEKG

>AtbHLH156

MGVLLREALRSMCVNNQWSYAVFWKIGCQNSSLLIWEECYNETESSSNPRRLCGLGVDTQGNEKVQLLTNRMMLNNRIILVGEGLVGRAAFTGHHQWILANSFNRDVHPPEVINEMLLQFSAGIQTVAVFPVVPHGVVQLGSSLPIMENLGFVNDVKGLILQLGCVPGALLSENYRTYEPAADFIGVPVSRIIPSQGHKILQSSAFVAETSKQHFNSTGSSDHQMVEESPCNLVDEHEGGWQSTTGFLTAGEVAVPSNPDAWLNQNFSCMSNVDAAEQQQIPCEDISSKRSLGSDDLFDMLGLDDKNKGCDNSWGVSQMRTEVLTRELSDFRIIQEMDPEFGSSGYELSGTDHLLDAVVSGACSSTKQISDETSESCKTTLTKVSNSSVTTPSHSSPQGSQLFEKKHGQPLGPSSVYGSQISSWVEQAHSLKREGSPRMVNKNETAKPANNRKRLKPGENPRPRPKDRQMIQDRVKELREIIPNGAKCSIDALLERTIKHMLFLQNVSKHSDKLKQTGESKIMKEDGGGATWAFEVGSKSMVCPIVVEDINPPRIFQVEMLCEQRGFFLEIADWIRSLGLTILKGVIETRVDKIWARFTVEASRDVTRMEIFMQLVNILEQTMKCGGNSKTILDGIKATMPLPVTGGCSM

>AtbHLH157

MGSEYKHILKSLCLSHGWSYAVFWRYDPINSMILRFEEAYNDEQSVALVDDMVLQAPILGQGIVGEVASSGNHQWLFSDTLFQWEHEFQNQFLCGFKILIRQFTYTQTIAIIPLGSSGVVQLGSTQKILESTEILEQTTRALQETCLKPHDSGDLDTLFESLGDCEIFPAESFQGFSFDDIFAEDNPPSLLSPEMISSEAASSNQDLTNGDDYGFDILQSYSLDDLYQLLADPPEQNCSSMVIQGVDKDLFDILGMNSQTPTMALPPKGLFSELISSSLSNNTCSSSLTNVQEYSGVNQSKRRKLDTSSAHSSSLFPQEETVTSRSLWIDDDERSSIGGNWKKPHEEGVKKKRAKAGESRRPRPKDRQMIQDRIKELRGMIPNGAKCSIDTLLDLTIKHMVFMQSLAKYAERLKQPYESKLVKEKERTWALEVGEEGVVCPIMVEELNREGEMQIEMVCEEREEFLEIGQVVRGLGLKILKGVMETRKGQIWAHFIVQAKPQVTRIQVLYSLVQLFQHHTKHDDLLS

>AtbHLH158

MASADKLINTDVPEKDVFAFHFLQSLSNLRKQNPFDTPDQKNYRVRKIKKAAYVSMARAAGGSSRLWSRALLRRADKDDNKIVRFSRRKWKISSKRRRSNQRAPVVEEAAERLRNLVPGGGGMETSKLMEETAHYIKCLSMQVKVMQCLVDGLSPK

>AtbHLH159

MQPTSSMNEEFLKKWQMGLQIFRPSIDNTSVHERKKAIKLSADVAMASLRKGTTCWSRALIEKTATEDNFLVRQMLSGIKAETLINKKLPKKTVCHRKIVRRSKKILRRKSKSASEEAAAKAKRLVKRRTQGLRNVVPGGELMSNDVLLLQETLDYIVSLQTQVNVMRSIVDAAEAEIER

>AtbHLH160

MSSQPNHQTSISSLLHDRLHIPPAETIVEKESAEKDTCQSQRKRKEPVLHEVDGSSSGAAKKQDHNAKERLRRMRLHASYLTLGTLLPDHSSSSSKVLFSLLLLQVRYVLLVVELYITFLADWQKKWSAPSIIDNVITYIPKLQNEVGELTLRKQKLVELERRGPSIRAISVLELGESGYEAVVQICLKKENEDEFSNLLHVMEVQGLSVLSASTSQVCREQRVVCYNFHVKVLCLILSSSFLFSLCFDRMETMC

>AtbHLH161

MSSRKSRSRQTGASMITDEQINDLVLQLHRLLPELANNRRSGKVSASRVLQETCSYIRNLSKEVDDLSERLSQLLESTDSAQAALIRSLLMQ

>AtbHLH162

MEPSHSNTGQSRSVDRKTVEKNRRMQMKSLYSELISLLPHHSSTEPLTLPDQLDEAANYIKKLQVNVEKKRERKRNLVATTTLEKLNSVGSSSVSSSVDVSVPRKLPKIEIQETGSIFHIFLVTSLEHKFMFCEIIRVLTEELGAEITHAGYSIVDDAVFHTLHCKVEEHDYGARSQIPERLEKIVNSVH

>LOC_Os01g70310.2

MDEAEAAAAAAKMDELAGGGGGGGGDWSYLAADALAAASFTAFPFHHHHHHHHRDVLSAS

TPSSLLLNMDAATAAAMFDFQAAFPSSSVPPPPPTTTAALPPFHDFASSNPFDDAPPPFL

APPGQKLGFLGPPGGAFGGGMGWDDDDEIEQSVDASSMGVSASLENAAPVAAGGGGGGGG

GGGRGKKKGMPAKNLMAERRRRKKLNDRLYMLRSVVPKISKMDRASILGDAIEYLKELLQ

RINDLHNELESAPSSSLTGPSSASFHPSTPTLQTFPGRVKEELCPTSFPSPSGQQATVEV

RMREGHAVNIHMFCARRPGILMSTLRALDSLGLGIEQAVISCFNGFAMDVFRAEQCRDGP

GLGPEEIKTVLLHSAGLQNAM

>LOC_Os11g32100.1

MLPRFHGAMWMQDDGGGDQEHGQAAPPGQEQHHHDQHLMALAAAAAGGAGFGAAQAPAPL

LDEDWYFDAAGGGGGGAHGSMMLGLSSVHGGIGAGTSGGGHGQQFSLLNMGAAAAPFDVS

GFDLGIACGGVGGGGDVVSFLGGGNASNTALLPVGNAGFLGTFGGFGTAASQMPEFGGLA

GFDMFDAGAVNTGGSSSSSSAAAAAASASAHVSNTAPFSGRGKAAVLRPLDIVPPVGAQP

TLFQKRALRRNAGEDDDDKKRKAAAGAGAGALSADGADMVLDDGDDDGLSIDASGGLNYD

SEDARGGEDSGAKKESNANSTVTGDGKGKKKGMPAKNLMAERRRRKKLNDRLYMLRSVVP

KISKMDRASILGDAIEYLKELLQKINDLQNELESSPATSSLPPTPTSFHPLTPTLPTLPS

RIKEEICPSALPSPTGQQPRVEVRLREGRAVNIHMFCARRPGLLLSAMRAVEGLGLDVQQ

AVISCFNGFTLDIFKAEQCKDGPGLLPEEIKAVLMQSAGFHTMI

>LOC_Os03g04310.1

MELDEESFLDELMSLRRDGSAPWQAPPYPGGGGGGGGGGMMMSDLLFYGGDGGSAEARGG

MDASPFQELASMAAPPPQHPHEEFNFDCLSEVCNPYRSCGAQLVPSEAASQTQTQLTPLR

DAMVAEEETSGDKALLHGGGGSSSPTFMFGGGAGESSEMMAGIRGVGGGVHPRSKLHGTP

SKNLMAERRRRKRLNDRLSMLRSIVPKISKMDRTSILGDTIDYVKELTERIKTLEEEIGV

TPEELDLLNTMKDSSSGNNNEMLVRNSTKFDVENRGSGNTRIEICCPANPGVLLSTVSAL

EVLGLEIEQCVVSCFSDFGMQASCLQEDGKRQVVSTDEIKQTLFRSAGYGGRCL

>LOC_Os10g39750.1

MELMDDDGSSSLLEELMAPLRRGTPTTTPEDLWLQAYPMMMSPMCGDGVMLGDLLVGGGN

ARNTLASPPPPSFPLPVPLTTTTPCPPLHEVSFEFDSIDCLGEVCNPYKRSGGAVRATAA

AQVMVAAMDPRREAASSAVAVAAVEEEERCKARRGAGGGGDSGELAPMFVFGGGGGAAAS

VRPRSCRPPQPGAPSKNLMAERRRRKRLNDRLSMLRSVVPRISKMDRTSILGDTIGYVKE

LMDRIKNLQVEAATGDSSSSSTENLSMLKLNTLKPPPSSSSGEETPLIRNSTRFEVERRE

NGSTRIEMACAAIPELLPSTLAALEALGVEIEQCVISCFDDFAMQASCLQDDKKREMTRD

TEEIKQTLFRSAGYGDGCLI

>LOC_Os02g02820.1

MGRGDHLLMKNSNAAAAAAAVNGGGTSLDAALRPLVGSDGWDYCIYWRLSPDQRFLEMTG

FCCSSELEAQVSALLDLPSSIPLDSSSIGMHAQALLSNQPIWQSSSEEEEADGGGGAKTR

LLVPVAGGLVELFASRYMAEEQQMAELVMAQCGGGGAGDDGGGQAWPPPETPSFQWDGGA

DAQRLMYGGSSLNLFDAAAADDDPFLGGGGGDAVGDEAAAAGAWPYAGMAVSEPSVAVAQ

EQMQHAAGGGVAESGSEGRKLHGGDPEDDGDGEGRSGGAKRQQCKNLEAERKRRKKLNGH

LYKLRSLVPNITKMDRASILGDAIDYIVGLQKQVKELQDELEDNHVHHKPPDVLIDHPPP

ASLVGLDNDDASPPNSHQQQPPLAVSGSSSRRSNKDPAMTDDKVGGGGGGGHRMEPQLEV

RQVQGNELFVQVLWEHKPGGFVRLMDAMNALGLEVINVNVTTYKTLVLNVFRVMVRDSEV

AVQADRVRDSLLEVTRETYPGVWPSPQEEDDAKFDGGDGGQAAAAAAAAGGEHYHDEVGG

GYHQHLHYLAFD

>LOC_Os04g23550.1

MDAEMAMGESFAYYWETQRYLESEELDSMYLPTQDDSNYESSSPDGSHSSSAPAPAAVGG

DAAAAVAGSGGGMTTMMMGGGGGGGDDAGGANKNILMERDRRRKLNEKLYALRSVVPNIT

KMDKASIIKDAIEYIQRLQAEEQQMLREVAALESAAAASAAPAAANPFAGLGADEEHEYG

HHHPSSSSERTKKVKRALSVSSISDALLAAAAPAPPVEIQELRVSEVGDRVLVVSVTCSK

RRDAMARVCRALEELRLRVITANITSVAGCLMHTLFVEVDHMDSVQMKQMVEAALSQLVA

TGSPLSSMSY

>LOC_Os04g23440.1

MEAAADMGVLLDLYWHTPHIFDLAVADSLLMDGFGGGMYGPAAAPATPAMVEKEYDESLS

ELYAYTSQSRYADSSSPDVVNLCSTAVASAAAAASSKNIAMERDRRKRLNEKLFALRAVV

PKITKMDKASIVRDAIAHIEKLQEEERQLLDEISVLQSAAAVAATAVEDVDDSGVTMPSM

KKLRSTPPLDGGGGALRVASSPPLQILEVETKETEDFYGLILQTSQSWPSDPQLQVSKVG

EKTVAVSIRCAKTRGAMAKVCHAVESLYLKVVSASVAAVDGTIVHTMFVEVTN

>LOC_Os01g13460.1

MVMKMEADEDGANGGTGGTWTDEDRALTASVLGTDAFAYLTKGGGAISEGLVAASLPVDL

QNRLQELVESDRPGAGWNYAIFWQLSRTKSGDLVLGWGDGSCREPHDGEMGPAASAGSDE

AKQRMRKRVLQRLHSAFGGVDEEDYAPGIDQVTDTEMFFLASMYFAFPRRAGGPGQVFAA

GVPLWIPNTERNVFPANYCYRGYLANAAGFRTIVLVPFETGVLELGSMQQVAESSDTLQT

IRSVFAGAIGNKAGVQRHEGSGPTDKSPGLAKIFGKDLNLGRPSAGPGTGVSEADERSWE

QRTGGGSSLLPNVQRGLQNFTWSQARGLNSHQQKFGNGILIVSNEATPRNNGGVDSSTAT

QFQLQKAPPLQKLPQLQKSHQLVKPQQLVSQQQLQPQAPRQIDFSAGTSSKPGVLTKKPA

GIDGDGAEVDGLCKDEGPPPALEDRRPRKRGRKPANGREEPLNHVEAERQRREKLNQRFY

ALRAVVPNISKMDKASLLGDAITYITDLQKKLKEMEVERERLIESGMIDPRDRTPRPEVD

IQVVQDEVLVRVMSPMESHPVRAIFQAFEEAEVHAGESKITSNNGTAVHSFIIKCPGAEQ

QTREKVIAAMSRVMNSG

>LOC_Os10g42430.1

MWVLLSPLLTTKNPFHPIPIPTFPLLLFSSSLVGVLFQIKSNLEEEEIEIKSMNLWTDDN

ASMMEAFMASADLPAFPWGAASTPPPPPPPPHHHHQQQQQQVLPPPAAAPAAAAFNQDTL

QQRLQSIIEGSRETWTYAIFWQSSIDVSTGASLLGWGDGYYKGCDDDKRKQRSSTPAAAA

EQEHRKRVLRELNSLIAGAGAAPDEAVEEEVTDTEWFFLVSMTQSFPNGLGLPGQALFAA

QPTWIATGLSSAPCDRARQAYTFGLRTMVCLPLATGVLELGSTDVIFQTGDSIPRIRALF

NLSAAAASSWPPHPDAASADPSVLWLADAPPMDMKDSISAADISVSKPPPPPPHQIQHFE

NGSTSTLTENPSPSVHAPTPSQPAAPPQRQQQQQQSSQAQQGPFRRELNFSDFASNGGAA

APPFFKPETGEILNFGNDSSSGRRNPSPAPPAATASLTTAPGSLFSQHTPTLTAAANDAK

SNNQKRSMEATSRASNTNNHPAATANEGMLSFSSAPTTRPSTGTGAPAKSESDHSDLEAS

VREVESSRVVAPPPEAEKRPRKRGRKPANGREEPLNHVEAERQRREKLNQRFYALRAVVP

NVSKMDKASLLGDAISYINELRGKLTALETDKETLQSQMESLKKERDARPPAPSGGGGDG

GARCHAVEIEAKILGLEAMIRVQCHKRNHPAARLMTALRELDLDVYHASVSVVKDLMIQQ

VAVKMASRVYSQDQLNAALYTRIAEPGTAAR

>LOC_Os01g50940.1

MSWSETDAALFAAVLGHDAAHHLATTPPHLDAPEGSPSSAELQASLHDLVERQGGAWTYG

IFWQESRGAGAASGRAARAVLGWGDGHCRDGAGHGEVGAAERSVARKRVLLRLHALYGGG

DEDGADYALRLDRVTGAEMYFLASMYFSFPEGSGGPGRALASGRHAWADVDPHPSGSGSA

PGWYVRSSLAQSAGLRTVVFLPCKGGVLELGSVVAIRETPEVLRAIQSAMRAVPAPPEDF

MRIFGKDLSPGRPSQPMGCDAPWTPRLVVQTTPVRPAKKEVVKAKPAEPPKSLDFSKANV

QEQAGGQERRPRKRGRKPANGREEPLNHVEAERQRREKLNQRFYALRAVVPKISKMDKAS

LLSDAIAYIQELEARLRGDAPVPARADGPAVEVKAMQDEVVLRVTTPLDEHPISRVFHAM

RESQISVVASDVAVSDDAVTHTLMVRSAGPERLTAETVLAAMSRGVSVTTPSP

>LOC_Os08g43070.1

MDELVLSPSSFSATACFPTLDFEFCEVPDQWLLGLGHDELDKDAAASALAAAAASQSASN

DDVPRNPPATTTTTKRRGRKPGPRSGGGGAPPIGHVEAERQRREKLNRRFCELRAAVPTV

SRMDKASLLADAVDYIAELRRRVERLEAEARRAPLAPSAAAAAAWAAGLGAGAIGRDDLV

VRMIGRDAAILRLTTAAAAARHAPARMMCAVRALNLAVQHASVARVGGATVQDVMVDDVP

AALQDEARLRAALLHTLQLADTT

>LOC_Os01g39480.1

MVPEEPNVVNRITTAFWEFQLLACSDEPISSGTPSSPSSPLTKETGDANTVLIDDLFLAH

SDAILAGGDQEDHQLGNDLGQQQAATAMEIDDDMIYSLIRNWDNDSSSSWIELLDHVVVS

PASCFVPWKRTELDKQAVAGGGEAAQRLLKKAVGGGGAWMNRAAGSSIKNHVMSERRRWE

KLNEMFLTLKSLVPSIDKVDKASSLAETIAYLKELERRVQELESGKKVSRPAKRKPCSER

IIGGGDAGAVKEHHHWVLSESQEGTPSNVRVIVMDKDELHLEVHCRWKELMMTRLFDAIK

SLRLDVLSVQASAPNGLLGLKIRAKVVSLT

>LOC_Os04g47080.1

MEETPLPSGKNFRSQLAAAARSINWTYAIFWSISTSRPGVLTWKDGFYNGEIKTRKITNS

MNLMADELVLQRSEQLRELYDSLLSGECGHRARRPVAALLPEDLGDTEWYYVVCMTYAFG

PRQGLPGKSFASNEFVWLTNAQSADRKLFHRALIAKSASIKTIVCVPFIMHGVLELGTTD

PISEDPALVDRIAASFWDTPPRAAFSSEAGDADIVVFEDLDHGNAAVEATTTTVPGEPHA

VAGGEVAECEPNSDNDLEQITMDDIGELYSLCEELDVVRPLDDDSSSWAVADPWSSFQLV

PTSSPAPDQAPAAEATDVDDVVVAALDSSSIDGSCRPSPSSFVAWKRTADSDEVQAVPLI

SGEPPQKLLKKAVAGAGAWMNNGDSSAAAMTTQGSSIKNHVMSERRRREKLNEMFLILKS

VVPSIHRVDKASILAETIAYLKELEKRVEELESSSQPSPCPLETRSRRKCREITGKKVSA

GAKRKAPAPEVASDDDTDGERRHCVSNVNVTIMDNKEVLLELQCQWKELLMTRVFDAIKG

VSLDVLSVQASTSDGLLGLKIQAKFASSAAVEPG

>LOC_Os11g15210.1

MYDDNGAADLPTSQSASIKTIVCVPFIMHGVLELGTTDPVSEDPALVDRITASLWDTPPR

AAFSSEAGVADIVVFEDLDHGNTAVEATTTMVPGEPEPHAVAGGEVAECESNAHNDLEQI

TMDDIGELYSLCEELDVLDDDSSSWVADPWSSFQLVPTAEATDVDDAVVAALGAIDGSCR

PSPSSFVAWKRTPDSDEVQAVPLISGEPPQKLLKKAVAGAGAWMNNADGSAATMTTDQGS

SIKNHVMSERRRREKLKEMFLILKSVVPSIHKVDKASILAETIAYLKELEKRVEELESSS

QPSPRPMETTRRRCCKSTGKKVSAGARAKRKAPAPEDTDGERRHCVSNVNVTIMDNKELL

LELQCQWKELLMTRVFDAIKGVSLDVLSVQASTSDGLLGLKIQAKVVVSAAKSSQQICSI

VYLSIYQSLYLRLFGVLILAILLLHACSLPHLLPSNLG

>LOC_Os04g47040.1

MASAPPVQEEPLQPGTNHFRSLLAAAVRSISWSYAIFWSISTSCPGVLTWNDGFYNGVVK

TRKISNSADLTAGQLVVQRSEQLRELYYSLLSGECDHRARRPIAALSPEDLADTEWYYVV

CMTYSFQPGQGLPGKSYASNASVWLRNAQSADSKTFLRSLLAKSASIQTIICIPFTSGVL

ELGTTDPVLEDPKLVNRIVAYFQELQFPICLEVLMSTSPSPNETEDADIVSEGLITHNAI

EEGQMVVSDECVSNANRDPITMEIDELYSIYEDLDLDMDLDLDTVRFLEDNGWPVNPSSF

QLVPASSTEAVAAAAAANDVDGVANSQVSCFMAWKSAKSNEMAVPVVTGIESQKLLKKVV

DCGARMSTGRGSRAALTQESGIKNHVISERRRREKLNEMFLILKSIVPSIHKVDKASILE

ETIAYLKVLEKRVKELESSSEPSHQRATETGQQRRCEITGKELVSEIGVSGGGDAGREHH

HVNVTVTDKVVLLEVQCRWKELVMTRVFDAIKSLCLDVLSVQASAPDGLLGLKIQAKFAC

SGSVAPGMISEALQKAIGG

>LOC_Os04g47059.1

MQAIDLFGCAMLSLQIAKPFLRALLAKSASIQTIVCIPFMSGVLELGTTDPVSEDPNLVN

RIVAYLKELQFPICLEVPSSTPSPDETEDADTVFDGLIEEDQMVILQGEDELGDVVVAEC

ETNGANPETITMETDEFYSLCEELDLDLGSYQLVPTSARETVAAAAAAANDVDGVAYSHA

SCFVSWKRANPAEKVVAVPMTAGIESQKLLKKAVGGGTAWMSNIDDRGSVAITTTPGSNI

KSHVMSERRRREKLNEMFLILKSLLPSVRKVDKASILAETITYLKVLEKRVKELESSSRE

PSRWRPTEIGQGKAP

>LOC_Os07g11020.1

MAGGEAHAALQAVAQSLRWTYSLLWQLCPHQGSSLVWGEGHYNGAVKTRKSTVMQPPPAE

EEDDADHAARHRSRQLRELYDWLQQAGENSSGGVQTSSTTASRRPGAALSPEDLTETEWF

FLMSASYSFPPGIGLPGRAFARRGHVWLTGANEVDSKVFLRAILAKTVVCIPVVDGVLEI

GTTEKVEEDMGLIQYARGIFMDQHGIHMKPTLSQHSTSNPVTHCTHQHPIQVQMQLGITS

QTKFDYSDELNADEENDDTEEEGMSGSDTNNTDTERNSGQLQLQMQDQLNMVSNDHQTIP

NNAVSSELMQCEMSEVVRDGCSNNILEDEIQMLMDCQNSNCQLNLQGPDEPCHSWHFLCE

ELQNDYQPATEDQVASPENTHYPKTLMTILHYNTLRQQEMNIKNYLPVSEKSSFSRWTTP

EGSDDNKTMISPGTTQRMLKSILMIVPSSHCSYRGAETPESRGGKGASGCHPR

>LOC_Os03g51580.1

MATQWFSNMVMDEPSFFHQWQSDGLLEQYTEQQIAVAFGQAGEADAAAAAAAMMVQQQQY

AAAAAAEHRPRKAAKVNTSWDSCITEQGSPADSSSPTILSFGGHADAAAAAAFASAGQAQ

SAPYYGGASAAALKPKQELDAAAAPFSQARPVKRSYDAMVAADVAKAPAAAASRPASQNQ

EHILAERKRREKLSQRFIALSKIVPGLKKMDKASVLGDAIKYVKQLQDQVKGLEEEARRR

PVEAAVLVKKSQLSADDDDGSSCDENFDGGEATAGLPEIEARVSERTVLVKIHCENRKGA

LITALSEVETIGLTIMNTNVLPFTSSSLDITIMATAGENFSLSVKDIVKKLNQAFKLSL

>LOC_Os03g12760.1

MSTLSNKVSLSSNLLNLQTGLAEEPEELTYMYHQEEHARMQEQFAGTPLVEQPVRFDQFY

PASMAPNQFHPSHCSSFPAFGGSSALPSLAFGAVATTKKEQVQQPSPSSSNVLSFAGQVQ

GSTTTLDFSGRGWQQDDGVGVFQQPPERRSRPPANAQEHVIAERKRREKLQQQFVALATI

VPGLKKTDKISLLGSTIDYVKQLEEKVKALEEGSRRTAEPTTAFESKCRITVDDDDGGSA

SSGTDDGSSSSSSPTVEASIHGNTVLLKICCKERRGLLVMILSELEKQGLSIINTSVVPF

TDSCLNITITAKARLALPVYYS

>LOC_Os03g46860.1

MEDSSLFMEWAMETLQHLHPLPATPPPAGGGYAGDNATFPSLQALRESSVSQNGMAPPEP

TAHEGHRASNSWSSGDTDSVSGGGGGAVMEHDGWSTSPNSVRCAAGGGGGGGGGGLWPVS

WNFSSAMTQPCNDQATPSNPPTTTRARYGGGGVRYLPAAVSPSPSAQTRRASSKGNGGGG

SGSSSAAPYAQEHIIAERKRREKINQRFIELSTVIPGLKKMDKATILSDAVRYVKEMQEK

LSELEQHQNGGVESAILLKKPCIATSSSDGGCPAASSAVAGSSSSGTARSSLPEIEAKIS

HGNVMVRIHGENNGKGSLVRLLAAVEGLHLGITHTNVMPFSACTAIITIMAKVEDGVSVT

AEDIVGKLNTVLQQNSRNSARETKS

>LOC_Os12g43620.1

MDDSSFFMQWAMDTLHQLPSDSTAAAAYATDVAGDSGAFPSLQALRNASAAGGGGGFRDL

TVQVDQVHRANSWSSSDSPGGGAATAAAGWSPHVTGGGGRGHRPMSWNFSAASAQPTTED

SGGGGGGGVVPAPLQAMETTATARAAVKKGGGGGSSSSAAAPGYVQDHIIAERRRREKIN

QRFIELSTVIPGLKKMDKATILGDAVKYVKELQEKVKTLEEEDGGGRPAAMVVRKSSCSG

RQSAAGDGDGEGRVPEIEVRVWERSVLVRVQCGNSRGLLVRLLSEVEELRLGITHTSVMP

FPASTVIITITAKASSLSNHPALLCLYICIKLPCVISS

>LOC_Os03g46790.1

MDDSSLFLHWAVSTLQHQHPAAVAVVADDDATFFSFQELCDTEEVVVVPVQEEVITEAHG

GGASRIGLAVAVDEHGGWSRSPNPGARPPSGGCGSNNLPLMSWDFSAASVAVQLEHVVAE

RKRREKINQRFMELSAVIPKLKKMDKATILSDAASYIRELQEKLKALEEQAAARVTEAAM

ATPSPARAMNHLPVPPEIEVRCSPTNNVVMVRIHCENGEGVIVRILAEVEEIHLRIINAN

VMPFLDQGATMIITIAAKASSSLLY

>LOC_Os10g01530.1

MQQLAESLANELFNQPQEQQEEQHGYHNPSLRVLPFVGDINKPEGHTPAAAAIRDSFFSL

TNGSSSSLNFSALEQQQDSGPMTKFCSPLSEMKRGGRRATSSMQEHVIAERKRREKMHQQ

FTTLASIVPEITKTDKVSVLGSTIEYVHHLRERVKILQDIQSMGSTQPPISDARSRAGSG

DDEDDDGNNNEVEIKVEANLQGTTVLLRVVCPEKKGVLIKLLTELEKLGLSTMNTNVVPF

ADSSLNITITAQIDNASCTTVELVKNLKSTLRNF

>LOC_Os01g39330.1

MDEVWCSLDSMPADIAAGIHQPPPPDLHDPSFWPAFADCAASFIAGGGGDNACFDELMAGGSSGDTRMVAMDDGDDGSGFLVGDAEAEHLMLSSSSPSSLSSGRSLSIDSAGSMSSFSLD

AAAALAMSTLAVPHPYPPPVAHGMFASGDGGGGGGGAVDDHEDAIMRAMMAVISSASASPSSSGGSASSPTPFSRDSGAHHQPAGQPAMAAPQQPRGGNGGHVVVKSSSSSGGLAVPMDQKPGGGGRGRQQEEAAAASATNSSQLYHMMSERKRREKLNDSFHTLRSLLPPCSKKDKTTVLINAAKYLKSLETEITELEGTNTKLEKHIAGGGGAADAAMRARRAQQRAKVQISKAADSQSQQLVSLTVMVMVECDVVELVLHILECLRWMKEISVLSVYADTYSPQLLLKAIANIKLQIVGGDWNEASFHEAMTKAANDATISCAPLAITAAQ

>LOC_Os01g09990.1

MEDCSSWIHGYANANATAGNNGFMCGYAASCSPVEFQQQQQLVGSQIEHHLNQISMQMGMDDESAVYDGASMVDVLLMASSSPHHHAGAGSFQYSSPTSSSASFRSASVSCSPESSAAATTHFLGPPAPSAAAAGFHYPEVSSQAPLPLPLPPYEPQHGQYTTVLSPPPPAPELPATTTP

ATGGAFRRYARHLRPRRLPKPGGCGQRMFKTAMSVLTKMHVAATYNRQYYYQQAAAAAASASAAEAPPSGNQLQHMISERKRREKLNDSFLALKAVLPPGSKKDKTSILIRAREYVKSLE

SKLSELEEKNRELEARLASRPAAAAKNDKGETAAAPAPEAGDETKRKDLVEIEVTTSGGG

AGAADAAAAAGGDQETCTLNVDLRGGGGGGGMSTTDVVLRTLQCLREQIGDGASLVAMST

SAGSGGRPPRANLTLQLKV

>LOC_Os01g09930.1

MDSSSWIHGYTNANATGANSGFMCGYAASPVEFPQQQQLISMQMGMDDESAVYDGASMVG

DLLMASSSAHHAGAGSFQYSSSTTSSSASFRSASVSCNPESSAAAAPELPAATGGAFSRY

ARHLRPRRPPKPGACGQRMFKTAMSVLANMHVAATYRRQYYYQQAAAAAAAEAAPAPPSD

NQLQHMISERKRREKLNDSFVALKAVLPTGSKKDKASILIRAREHIKSLESKLSELEEKN

RELEARLASRPAAKNDKGETAAAEAGDETKREDLVEIEVTTTSGGSGAAAAAATGGDQET

CCTLNVDLRGGGGGGMSTTDVVLRTLQCLREQIGDGASLVAMSTSAGSGGRPPSANLTLQ

LKSPTYGRCSYKYEFTLFLVNRQKPTIKHIRYGSRKISMSLSMDDVTMISPR

>LOC_Os01g09900.1

MDSGGWIVHGYTNGATATGAGNHGFTCGYAASSCGSWEFEQREQQFISSQIQHRLNEISM

HLSMDDDDDQSAVYGVAMVDDLLTPSPSTHHAVAAAAGSFPSSSSSSASFRSASVSYSPD

TSSSAAAAAATGFYPELSSQVAPLLPPPPLVRNEPQPGRYTAGLPPPPPPPVTGGAFRRY

ARHLGPRRAPKPGACGQRMFKTAMSALAKMHMETTYRRRQYYYQQAAAAAEAAPPPPSGN

NQLQHTMSERKRREKLNDSFVALKAVLPPGSKKDKTSILIRAREYVKSLESKLSELEEKN

RELEARLSTRPDDTKNDEEEVAAPPEAGGEVKREDPVEIEALDNFFIR

>LOC_Os05g11070.1

MDGGSWMLKAAAAGDMHGGAGDTMIRCSWSDMATTDQLLRHHEQEPAMTMMMNSQSQAMQ

QQLSQIYMLMDMEEHDHQYATPPSPSSSSFRSFSAGTTTTTTSRDDNSSLMLAAAAASCH

HQTTEVSSQILLPRPGQAARRSSGGHGAAAAATAFRPYSRYLGPKKHLLRRPGAATTATG

GGGGQRAFKKAISVLSKIHAARLAQYYQIMEMAARASPAATAGGGGGENQQLQLQHVLSE

RKRREKLNDSFKALRDVLPPATKKDKASVLMRAKDYVNVLKARIAELEEKNRKLSESQQL

HAGDGDGERDDGPDDDKIEVNTSRSAADQGSSPNKCQELHLKIVLGSSSGCSAMDAVAGI

LQGLNEKRDVSLLATGHNSSSSSSSGRRRLLPRAKSSQQPAVRLQTLCKLSR

>LOC_Os02g12820.1

MDTIFVLGQESRQRILHRAAARLPGCAYVCAWAPLPLVAAAGLHHQRPSSGGAAGAARLL

YCVDGWLSGGEDGGGCVRALFDAYRGSVCGAVTGCVPGWAYVGGGGGVFMELSELELVAS

ASLPVQQSFYQEAGIKMAAFMGCESGEIEVGFSTAPAENYGGGGGGGSLQASVEQVFSED

FFQQSLLEELLQLPPTRPSSSSSSLVGSPADGAASTSLLRTMTPMMASSSATPSPRELAA

QVATTTTTTTTPSSSSRLHPRPPAPHHVHVSPFSRHGGVGGSGVLHFPSAEADDAAMAQA

MLDVISSPSTSSSAAALHAPWSSVKHRAQIIRSPRRGTPTTTAFRAYNAALAPRAAASRR

PPGAPGQRMIKMGFSILRRMHMVRCSQERAAAAAAAASAAAAQRSGGGDDEDATAAPPPP

TSSQLHHMISERRRRERLNESFEHLRGLLPPGSKKDKATVLAKTLEYMNLLIAQISELEA

KNRALQTQIHQRANGSSSSRSSMIRTVNEVHHHHNHQWLAVAAAAGGSPERVQVHVIGGG

DHDGGASASSSSSAPEVTVRVAVRAPERGGADVSELVLRVLALLKAMGGFTVVAVDARQP

GGGGGNGVAQASLTLRATVRMLTLLKRMFRAKNHSSSDQIIRL

>LOC_Os06g37410.1

MGRAAARLPGCLYLCLWAPAAAIAGGVQPNHLFCLDAWIGGGGGVGAGGGGGDRALELFE

AYRGALCAAVSGCVPGWAYKEGAACMELTEHDLAASASLQVQQQFYHETGTKMAVFMGCD

SGEIEVGLSAASATATAAVVGEMQQSILEELLQMPPPPPSPSSSSLLSLSVGSPEYSSLV

RSMATSVGASAAADPSPVHGGLLAPVYGEFPGSDDDAAMAQAMLAVISTPAPPPPLWRPP

RRRARSSSSPRRATAFKAYNAALSPRARPRPGAPGQRMIKTGISLLASVHMQTRSRELAA

ARQRDTHAAPPPPPPPPPPSSSQLHHMISERRRRERINDSFQTLRALLPLPPDSKKDKAA

ILASTTEYMDKLISQVSELGEKNRQLEAQLAARSGEAQWPAASGGGGGESSSERVQVDVV

IAGSSASTDQPREVSIRVTVRAECDVSELVVAVLARLREMGRFAVVSVDAGRRSSSFAQA

SLTFRVMNYKYKTNSVTVGKSQLIVHTEKYKAGDVCDETSLKEAVAKAVDGAAFFKFQRT

PFMHSKNYDQSDDLEGEAKLLPLDS

>LOC_Os08g38210.1

MGLQGNKATHDFLSLYAAAATATDYSPLPRHPDSKPSAPAPPPAQDFFLKTHDFLPQVEK

RADQTPPPPPPPPPHAAVTAEKQLLHQHALAAAGAFTINHAVAAAATAVKQEPPFAPWCQ

PVAAVDPRGHQWSLPFAARAVAVASSRPQQQQQQPPPPERKGGGGFMDAGSRSSGGAGFD

DDDGHAARREVSSSLKELTVRVEGKGGSCSGSAGTDQMPNTPRSKHSATEQRRRSKINDR

FQLLRDLLPHNDQKRDKASFLLEVIEYIRFLQEKVQKYEVSYPEWNQENAKVVPWTNIYF

RSSWKNAQNKGQVPADHSPDPPELLKNGSPYMFPFTGNSDNNNAVETAAASGAQDQAETD

PMSRVSYRSVDTPSPNNVADKVTSQPHAQLVRPSPAENHTVNCDKLNNSDLAIDEGTISL

SSQYSQELLNKLNHALENSGIDLSQASISVQINLGKRAMKRSTPAATSTSKELTDPASNS

QAMGRQLRLGDGAEEHRQASKRHKSDNS

>LOC_Os09g29930.1

MGIQGNKATTREHDFLSLYTTAAKDPSLQLHDAKPPPPSQGFFLRTHDFLQPLEKPTPAP

TPPPTSQLQQQQQQAFPGGIGTFSISHVAGARPVAAAVVKAEPTPFVLWGQPAAAAAAHP

VAALGHHHHHHQWTLPFAGVGQVAATAARQQQEWKGRVGGGGFMDSGSRSSGGAGFDDDD

GVAARREVSSSLKELTVRVDGKGGSCSGSGTDQRPSSPRSKHSATEQRRRSKINDRFQIL

RELLPHSDQKRDKATFLLEVIEYIRFLQEKVQKFEASVPEWNQENAKILPWSNIYFRSFW

KNSQSKGQNPGDDLPDPSQFIRNGSSSGYNFTGKPDDNHNMVTSAAASGAQELVETDHAA

SVSYRSAETPTNITNNVTSQAQAQWASPAGVDDCAMNSEMLNNQQLAIDEGTISLSSQYS

QQLLGTLTHALESSGVDLSQSSISVQINLGKRAVKRPGADGSSSSKELPSTSANNENMGH

QLTMLGGGTEELPHPTKRHKSGNS

>LOC_Os01g65080.4

MDSGLGRSSETSLKALPSMASNATRNTDPDQQGVRFSSMDQPPCFARPGQSFPAFPPLFG

VQSSSLYLPDDIEAKIGNQFESNPSPNNPTMDWDPQAMLSNLSFLEQKIKQVKDIVQSMS

NRESQVAGGSSEAQAKQQLVTADLTCIIIQLISTAGSLLPSMKNPISSNPALRHLSNTLC

APMILGTNCNLRPSANDEATIPDISKTHDYEELMNSLNTTQAESDEMMNCQNPCGGEGSE

PIPMEDHDVKESDDGGERENLPPGSYVVLQLEKEEILAPHTHFCLICGKGFKRDANLRMH

MRGHGDEYKTAAALAKPSKDSSLESAPVTRYSCPYVGCKRNKEHKKFQPLKTILCVKNHY

KRSHCDKSYTCSRCNTKKFSVIADLKTHEKHCGRDKWLCSCGTTFSRKDKLFGHVALFQG

HTPALPMDDIKVTGASEQPQGSEAMNTMVGSAGYNFPGSSSDDIPNLDMKMADDPRYFSP

LSFDPCFGGLDDFTRPGFDISENPFSFLPSGSCSFGQQNGDS

>LOC_Os02g49480.1

MQLFQGEEPAHDFLSLRAGGGSSSPPFQHREEQHSSHSSRGYGMEIRRSLRPLDLAKQRG

RSSGNGTAAGSAVDGASPAGSDSEEHVLPGGVGTFSIRHASGTPSREEAGSHGGVRRSAF

AFAPALHGARMENAHETGGRSGSRAHRAPSTMWQDSAIDQRSIGQTPYEATRAEGRSSAS

SADQGPSTPRSKHSATEQRRRTKINDRLEILRELLPHTDQKRDKASFLSEVIEYIRFLQE

KVQKYEEADPERNHEDSKSMPWAKVYYRSCWRNTKNTSQVQGEDLSPSTQDMNNEQYGPK

HISAAQPALFNTQSVTSTTTSSSHMATGTPQNLEKNSTPSNQPPWLSMSTMRQESEPGNK

MPNKHEKQTLHDENHSISSAYSQGSVP

>LOC_Os01g06640.1

MAECQPLQLQEGKKLQELQPYDGCNPSVYRGPILLPRQANSAPPAVPPEMSSSSGSGRSA

TEARALKIHSEAERRRRERINAHLTTLRRMIPDTKQMDKATLLARVVDQVKDLKRKASEI

TQRTPLPPETNEVSIECFTGDAATAATTVAGNHKTLYIKASISCDDRPDLIAGITHAFHG

LRLRTVRAEMTSLGGRVQHVFILCREEGIAGGVSLKSLKEAVRQALAKVASPELVYGSSH

FQSKRQRILESHCSIMSI

>LOC_Os05g07120.1

MAACQQQIWQEGKQQQHLHHGGYDDLSSVYRGTVVLPRRQGGLAPEPPPPRPSSSSGRSA

AAQATAMTIHSEAERRRRERINAHLATLRRILPDAKQMDKATLLASVVNQVKHLKTRATE

ATTPSTAATIPPEANEVTVQCYAGGEHTAAARTYVRATVSCDDRPGLLADIAATFRRLRL

RPLSADMSCLGGRTRHAFVLCREEEEEEDAAAEARPLKEAVRQALAKVALPETVYGGGGR

SKRQRLMMESRYSTAVVHTHVDPQYCWYNSR

>LOC_Os01g11910.1

MGSAPFGDAVAGGGLYEYQGYHGGFAGGHGLGQPAGRAPALDDGETEGMDASAAAAVAAM

EMAKRNCGGGREEKAAMALKSHSEAERRRRERINAHLATLRTMVPCTDKMDKAALLAEVV

GHVKKLKSAAARVGRRATVPSGADEVAVDEASATGGGGEGPLLLRATLSCDDRADLFVDV

KRALQPLGLEVVGSEVTTLGGRVRLAFLVSCGSRGGAAAAAMASVRHALQSVLDKASSGF

DFAPRAASLLGSKRRKVSTFESSSSSS

>LOC_Os08g33590.1

MWEAVGGGDGTALLPWPGSAAAAPLYMPPAAAAAAPFAAGEQLPVEQPFYFDGGGGVAGH

NHHPHHHQYGMEAPPPMTMMQMGGGGSSSSRMVVSGLLGTLQAELGRMTAKEIMDAKALA

ASRSHSEAERRRRQRINGHLARLRSLLPNTTKTDKASLLAEVIEHVKELKRQTSAMMEDG

AAGGEAAAAPVVLLPTEDDELEVDAAADEGGRLVARASLCCEDRADLIPGIARALAALRL

RARRAEIATLGGRVRSVLLIAAVEEEDPDEAGNDDDGEHGYGVAASHRRHELVASIHEAL

RGVMNRKAASSDTSSSGAGGGGGSIKRQRMISAHDQQGSFNSSGW

>LOC_Os09g24490.1

MWEGGSHDAAAQLLPWFVGEPAAAAVGGYGGCVDVVGQGGVFGFGFEAAAAPVVTRQQRG

GAAAAEGSSRGGGGKPAVVSGLLGSLQAELGRVTAREIMDAKALAASRSHSEAERRRRQR

INGHLARLRSLLPNTTKTDKASLLAEVIEHVKELKRQTTAIAAAAAAGDYHGNDEDDDDA

VVGRRSAAAQQLLPTEADELAVDAAVDAEGKLVVRASLCCEDRPDLIPDIARALAALRLR

ARRAEITTLGGRVRSVLLITADEQQQQHCDDVDDDEDGHRLLLRHGIDGAGAAADDDDEC

AASHRRHECIATVQEALRGVMDRRAAASSGDTSSSGGAVVAGGGGGSIKRQRMNYGVHEQ

CSV

>LOC_Os03g15440.1

MGAHGDHRHHHHHQEAGVLVDEEEEEVIEQACGGPTSGVVEQEVGGDGGGVCQDAAGMVF

EATSSVGSVSATMGPPPIMCWPPPAQPVHGAIHHHHNLGGGGGQQSPFFPLLPPLPPQPP

PPPPFFADFYARRALQYAYDHSGGASSSSDPLGLGGLYMGHHGSHVAGMMMPPPFAPSPF

GDLGRMTAQEIMDAKALAASKSHSEAERRRRERINAHLARLRSLLPNTTKTDKASLLAEV

IQHVKELKRQTSEITEEACPLPTESDELTVDASSDEDGRLVVRASLCCDDRTDLLPDLIR

ALKALRLRALKAEITTLGGRVKNVLVVTGDDSAAAAACAGTDGDGEQQEEAMQAPMSPQH

TVASIQDALRAVMERTASATEESGGSGAGGGLKRQRTTSLSAILENRSI

>LOC_Os03g59670.1

MFPVEVAAAAAAAGRMQGEAVVPMMLPPFFMDSGIWPAAAGVVDVAASAEEEAAAAAAAA

QDRALAASRNHREAEKRRRERIKSHLDRLRAVLACDPKIDKASLLAKAVERVRDLKQRMA

GIGEAAPAHLFPTEHDEIVVLASGGGGVGGAGGAAAVFEASVCCDDRCDLLPELIETLRA

LRLRTLRAEMATLGGRVRNVLVLARDAGGAGEGGDGDDDRAGYSAVSNDGGDFLKEALRA

LVERPGAAAGDRPKRRRVVSDMNMQAAA

>LOC_Os08g37290.1

MVTDGECSAAAVRKGGSPAVRSHSEAERKRRQRINAHLATLRTLVPSASRMDKAALLGEV

VRHVRELRCRADDATEGADVVVPGEGDEVGVEDEDDDEGERDEGCYVVGGGDRRWRRRVR

AWVCCADRPGLMSDLGRAVRSVSARPVRAEVATVGGRTRSVLELDVVVASDAADNDRAVA

LSALRAALRTVLLNREELLAAAAAAATDGYKRPRFSPRCSSLT

>LOC_Os09g28900.1

MVAARSGDDAELRLDVECLAAAPRWTRARRSHSEAERKRRERINAHLDTLRGLVPSASRM

DKAALLGEVVRYVRKLRSEAAGSAAVVPGEGDEVVVEEEEVEVEGCSCDAGERQAARRVK

ASVCCADRPGLMSELGDAERSVSARAVRAEIATVGGRTRSDLELDVARTAAAGGGSNGAS

QLPALQAALRAVIMSQEELLAVESYKQRRFSADFA

>LOC_Os03g08930.1

MALEAVVFPQGHFGYGCGRDSPAYGMPWCDVLAAAGGGGGFGEFYGVDEWDDQLQVASVD

EWEVASKDNSDASTEGKAAAAERAEPVAAGRRKRRRTKVVKNKEEIECQRMTHIAVERNR

RRQMNEYLAVLRSLMPASYSQRGDQASIVGGAINYVKELEQLLQSLEVQKSLKNRSGAMD

AAGDSPFAGFFSFPQYSTSPRTGCSAAASAGSSGSASSVVMDDTAGSAESGRQSAAIADI

EVTMVEGHASLKVLARRRPKQLLKLVVGLQQLRIPPLHLNVTTVDAMVLYSFSLKVEDDS

KLGSVEDIATAVHQILGSIQQQEAVLSIS

>LOC_Os10g23050.1

MALEAVVYSHGGHFGGYGGGLMGGAPAAPWDVFAAAGGGGSWDDPGLFAAEASLDVIQGV

DEWEVDQDQHASSSSKVAARPPVKAAAAAGKRKRRRAKAAKNREEIESQRMTHIAVERNR

RRQMNEYLAVLRSLMPPSYAQRGDQASIVGGAINYVRELEQLLQTLEARRTIKDHIDGGA

GESPSPFAGFFAFPQYSTATSGHGGGGDAHSRIVVKPAETTTTAAGGGAGAAIADIEASM

VEGHASVKVQARRRPRQLLKLVAGLHQLGLTTLHLNVTTVAAMAMYSFSLKVEDGCKLGS

VEEIATAVHEILERMQEEQAFADAKTSL

>LOC_Os09g29360.1

MALEAVLLSRGDLFGRRRCAMEGGGGGWSSPFSGFEGVMDLDGGNWDAAACSSMLLHGFQ

ELEIPAAAAAAAAMAPPPPPVEPANCAENAGGVGGHQEDQAAVAAAATAVQSGRRKRRRA

RAAKNKEEVESQRMTHIAVERNRRKQMNEYLAVLRSLMPPSYAQRGDQASIVGGAINFVK

ELEQLLQSLEARKSSRQCAAHDAAAAAAPFASFFTFPQYSMSAAAAAAPVAPVVNELHGR

DDGGAGTAEAEASGSKPSAVADVEVTMVESHANLRVLSRRRPRQLLRLVVALQGHRLTVL

HLNMTSAGHMVLYSFSLKVEDDCQLTSVDEIATAAHQIIEKIQEEQGCSLD

>LOC_Os08g37730.1

MALEAVVFSEGYYFGCGGAMAAEAAAGGAWSWSHGYGGGVDQGKGAMELVVDDGVVNAFW

DGGGGGASSSPVMAAVSGFIEEPDGGNSSVADAAPPPEHGGASVVGRDGSAAAPAAAAGR

RKRRRARTVKNREEVESQRMTHIAVERNRRKQMNEYLAVLRSLMPASYVQRGDQASIIGG

AINYVKEMEQLLQSLEAHRHARRARTDAAAALPFAGFFTFPQYSMSAVPTTTTTTVAAAA

TENGNAVAGAGAGDDADADVSGSKPSSVADIEVTMVESHANLKVLSRRRPRQLLRMVAGL

QHHRLAVLHLNVASAGHMALYSLSLKVEEDCQLTSVDDIAAAVHGIVETIEQEQQQQQKQ

SCS

>LOC_Os02g52190.1

MALEAVVFPQEHLSCAAAMYAAPPSSLGCGVDMDEFEENGGVVLQEEAGVAVHGGGGGGA

GGMMSLWVNAATCARSIAASGAVEEFWDERQYPVSSPPAAPTPAASGGGGGHAKAAARRR

RRRPKVVKNKEEAESQRRNHIAVERNRRRQMNEYLAVLRSLMPPSYAQRGDQASIVAGAI

NFVKELEQLLQSLEAQKRRAEHAPPAPPFAGFFTFPQYSTTVGDNNAAGSGAADGEGGCG

ARPGAADIEVAMAESHANVRVLAPRRPRQLLRMVVALQCLGLTVLHLNVTTTADHLALYS

FSLKMEDECRLSSVDEIAGAVNQMVTKIAGECIS

>LOC_Os02g46560.1

MSTLVDALCAPCSDTALIYDTFNASAAASFLFDNAAAFCDADILGATATGEKEATSSAAA

AAAEAAPPRKKRRRRAKSCKSREETETQRMTHIAVERNRRRQMNEYLAILRSLMPEPYVQ

RGDQASIVGGAIEFVKELEQQLQSLEAQKRTLLPHHKARCDDATPMHNASGSNVGAGGCM

EPTTTTSNCSSSVTEDAPSADAPPFAQFFAYPQYVWCHSPRDSTTTTTAASASASASASS

SSPATVAAALQSEHRSGLADIEVSLVETHASVRVMSPRRPGQLLKMIAGLQALRLTVLHL

NVTTLDSLVLYTLSVKVEEGCSLTTVDDIAAAVHHVLCIVDAEAAASEHLLAAGQLATTA

TAVAKRELATYMY

>LOC_Os04g50090.1

MALDTLCSAGGDVLIYDTFNASAAAAAAVVPPASFLFGNNNAGGAAGTETRVQVAAGAVP

EVDQLLKQAQQPGRRKRRRRARSCKSREDAESQRMTHIAVERNRRRQMNEYLAVLRSLMP

ESYVHRGDQASIVGGAIDFVKELEQLLQSLEAQKRTLLMQPPPPPQQQREPKCDAADSTS

AADQETPAAAAAAAAADGPPFARFFTYPQYVWCHNPAQDGGGGGGAAAENRAGVADIEVS

LVETHASIRVMAARRPGQLLKMVAGLQALRLTVLHLNVTALGSLALYSISVKRFHGGKAI

ASLGPLGVFFLCALYLWYSTTVVEEGCGMATVDDIAAAVHHVLCIIDAEAASQMLLAGEA

SG

>LOC_Os05g50900.1

MGQAGYFLCSEITASKLLTVTATRELKPPPPPRPSGRVLSRRARRRRSSVLAWRHRRCFG

GLILQTWKKNHTISAVQQRSHLERFAALDEAPPPAPEETVVTGGEMVDYMLGQPPPTTPG

PQSQVSFDKLTFSDVLQFADFGPKLALNQPAASDNGGGGGDDGDDDDDSYFLRFQSLPSL

PAVPPPRGGAAAAHQVVDEQEGSKQTVDAGGVSESTTLVQQADGGGGRAEKAGEQGKSGR

RKRPRTVKTSEEVESQRMTHIAVERNRRRQMNEYLRVLRSLMPGSYVQRGDQASIIGGAI

EFIRELEQLIQCLESQKRRRLYGGDAPAPPARPVADGAVLPAPMQQPPPATPPFFPPSIP

FPASSGAGDGTGAGVAKVALDLDASGGGEVGGGVREEMAENKSCVADIEVRVVGVDAMIK

ILSRRRPGQLIKTVAALEEMHMSILHTNITTIDQTVLYSFNVKIAGDARFSAEDIAGAVH

QILSFIDVNYTL

>LOC_Os03g03000.1

MEQWAAAAAADQAAASAGAGQMPFLALLHGAMEADGGGGGDGRKRHAAAAAFASCCPCPP

VADLDLLESCVTQAAAPPVTAPATRAERRRKRPRPRPRAAPPPEKRKKPEEAENQRMTHI

AVERNRRRLMNDHLASLRSLIPSNYIPRGDQATVVGGAIDYVKQLEQQLVALQAAAAERS

GVGVVAAAATAASDGVFVSPQYTSYSEARGGSGVDVEATAAVGGHVRVRVAGRRWTGRLV

RAVAAMEDLRLTVLHLAVTSVGHDAVVYCFNLKVKTTTTLHHLLLHVPSFF

>LOC_Os02g15760.1

MADGGGGGLCELFSDDHRDIRHVADADLFRILETWEECINGGAGGGGGGVSLAGVADQGA

AAASTGAGGGARTTTTTTAANGRRREGRDEEKGGGGGGGPPAQKKQKGSSSSSSSPAALA

AAVGDGDGAAKMSHITVERNRRKQMNEHLAVLRSLMPCFYVKRGDQASIIGGVVDYIKEL

QQVLRSLEAKKNRKAYADQVLSPRPSPAAAALMVKPTPPISPRFAAAAAAGVPISPRTPT

PGSPYNKHAAAAATARPPHPAAATSSCSVAYSMSPAMTPTSSSSTTTTTTHELSPAPAFL

PILDSLVTELAARGGASCRPLVIPSSAAAIAGIVGVPDVRVEFAGPNLVLKTVSHRAPGQ

ALKIIAALESLSLEILHVSICTVDDATVLSFTIKIGIECELSAEELVQEIQQTFL

>LOC_Os06g33450.2

MGDALCDQLLLVDSDGGEFIPHHADADADDLFTILETWEGCANVVAGGAPATTTTLGSPI

AAAACISGVVGGQNHQQLPEPAAAKTVPATNNKRREEEVADRDGDGDDDDGSPQKRRKCC

SPESSTTDVAAATTPKTAHIAVERNRRKQMNENLAVLRSLMPCFYVKRGDQASIIGGVVD

YIKELQQVLHSLEAKKQRKVYTDQVLSPRPPATVAASCCSPRPPQLSPRLPPQLLKSTPP

LSPRLAVPISPRTPPTPGSPYRLLRLPPPPPPASGSNYASPAMTPTHHETAAPSLDAIAA

ELSAYASRQALGGGLLLPDVKVEFAGANLVLKTVSQRSPGQAVKIIAALEGRSLEILHAK

ISTVDDTAVNSFTVKIGIECELSAEELVQVIQQTFT

>LOC_Os05g51820.1

MSHIAVERNRRRQMNDHLKVLRSLTPAFYIKRGDQASIIGGAIDFIKELQTLLQSLEAQK

KRRQQPQAHLISPASISASGGGSPSPTPSPRSLITSCSPTAAAGSSAGSSSSISPKDENK

QQLQLVAELAACCNSPMADVEARISGANVLLRTLSRRAPPVRIIALLESLHLEVLHLNIT

TMDDTVLYSFVLKIGLDCHLSVDDLAMEVHQSFMPPPAAHPDNHLHS

>LOC_Os01g72370.1

MEQLFVDDPAFASSMSSLEADIFSGAGQLPSSPWLDLDLDDDVQDLSMAPTTANAVSSGY

GSGGSGSHRKLSHNAYERDRRKQLNELYSSLRALLPDADHTKKLSIPTTVSRVLKYIPEL

QKQVENLERKKKELTTTSTTNCKPGVLGSQLMSEGMAPIVSATCINDMEIMVQVSLLSNV

AGSVLPLSKCIKVLENEGLHFISSSTSSGFGNRTFYSIHLQRSEGTINEECPAFCERLEK

VVRNKAKL

>LOC_Os07g35870.1

MASPEGSTWVFDCPLMDDLAAAAGFDAAPAGGFYWTTPAPPQAALQPPPPQQQPVAPATA

APNACAEINGSVDCEHGKEQPTNKRPRSESGTRPSSKACREKVRRDKLNERFLELGAVLE

PGKTPKMDKSSILNDAIRVMAELRSEAQKLKESNESLQEKIKELKAEKNELRDEKQKLKA

EKESLEQQIKFLNARPSFVPHPPVIPASAFTAPQGQAAGQKLMMPVIGYPGFPMWQFMPP

SDVDTTDDTKSCPPVA

>LOC_Os05g38140.1

MSCGGAGQSGWLLEYGLVEEEIQGSDFIYMVDDPAAVSSVLLGFDVPRKEDGSGGQDNSA

SKKRSRPESSAPPGTKACREKLRRDRLNERFNELSAILEPGKPPRADKVSILSDAARLLS

QLRAEAQKLKSSNESLQDSIKSLKAEKSELRDEKTRLKAERERLEQMLKGVGAATPAAPA

PFVPHHAAAVAAAPAYHPAAFAQAGGKYVPYATSYAPPAAFWQWIPPTSLDTSKDPVMWP

PVA

>LOC_Os02g02480.3

MDGGGDPVDEFLIGGGGEDGDLGVFCDGVPTLPCDGGLGIDDVSGDTCCLDQSVLGKRGR

DESSSSGPKSKACREKIRRDRLNDRFLELSSVINPDKQAKLDKANILSDAARLLAELRGE

AEKLKESNEKLRETIKDLKVEKNELRDEKVTLKAEKERLEQQVKALSVAPTGFVPHLPHP

AAFHPAAFPPFIPPYQALGNKNAPTPAAFQGMAMWQWLPPTAVDTTQDPKLWPPNA

>LOC_Os08g04390.2

MSGTPADGGGGGGGGGGGSGDDWFLDCGILEDLPAAACGAFPWDASPSCSNPSVEVSSYV

NTTSYVLKEPGSNKRVRSGSCGRPTSKASREKIRRDKMNDRFLELGTTLEPGKPVKSDKA

AILSDATRMVIQLRAEAKQLKDTNESLEDKIKELKAEKDELRDEKQKLKVEKETLEQQVK

ILTATPAYMPHPTLMPAPYPQAPLAPFHHAQGQAAGQKLMMPFVGYPGYPMWQFMPPSEV

DTSKDSEACPPVA

>LOC_Os11g38870.1

MPQVRNQNLGMVADTESSGSLGGSSNAASDKAVDGSLDKRSQEKAPKKTHKAEREKLKRD

QLNDLFVELSSMLDPERQNSGKATVLGDAARVLRDLVSQVESLRKEQSALLTERQYVGSE

NNELQEENIMLRAQILELHNEICARMGNNHLNQSNLAMSQPVANNGSNSATQPVPHHIWG

NGPNLAMVHPTNTLSPLHNQHHQSAGASQVYASRPQELQLFPGTSVSTERERSRAGSGST

PATSSGLTDSLPGQLRLSLPQSSQEENSSGSKKGRKKG

>LOC_Os07g43530.2

MVPRDRVNAAAAGGGGEGRLVQSGIVNKKCDKKAPKRIHKSEREKLKRDKQNDLFNELGN

LLEPDRQNNGKACVLGETTRILKDLLSQVESLRKENSSLKNESHYVALERNELHDDNSML

RTEILELQNELRTRMEGNPVWSHVNTRPALRVPYPTTGVFPVQHLPHLPVTTTAAFPQQL

PVIIEQHYAATPRELQLFPESATSEDSEPSQEHGISDHVTRPQPRYPTPTATLPVNLFPV

FPGRQDQQCSSGTSGTNEEDRIGRS

>LOC_Os03g26210.1

MGSFLVDWFSAGNGRGRFLEEAMVPSERGDVATAIRPAAADKLVHGPISDKKCRKKVPRK

VHKSEREKLKRGHLNDLFGELGNMLEADRQSNGKACILTDTTRILRDLLSQVKSLRQENS

TLQNESNYVTMERNELQDENGALRSEISDLQNELRMRATGSPGWGHGATGSPLPVPPSPG

TVFPSQQPMQPSPMTTSTVFPLQQPLPQPTVIEPSARQPLELKLFLEAPPAEDPEPSEDQ

EAPNNVARPQPRYPTEASSWPISLGLPRMEDEQM

>LOC_Os02g23823.1

MDPRSSQAQEDGFFHPRDGACPADSSGKTECKTQGSIATRKVQKADREKMRRDRLNEQFQ

ELGSTLDPDRPRNDKATILSDAIQMLKDLTSQVNKLKAEYTSLSEEARELTQEKNELRDE

KVSLKFEVDNLNTQYQQRMRVLYPWTGMEPSVVIGPPLPYPFSVPVPVPVPIPSGAVPMH

PQLQAYPYFRNQTSGTVSNPCTPYMAYTQPIHPPTDQLSNQFSAPVQHSSSNRSHSMAQD

SRSKSSALQQVSCRGKHDDFDDVATDLELKTPGSSAPLQSEIANKDSSSDLKKKQQFIQE

TKGSSLTEGSSSSSRCSSSGPPDVSNSIEGGSVADDQRSTVQT

>LOC_Os04g41570.2

MMMTEVANHSKRNHNESYFTGKAAVTSSSEEFGSMTSKKPRNTSPRDAPVSPKEKKDKIG

ERVAALQQLVSPFGKTDTASVLQEASGYIKFLHQQLEVLSSPYMRAPPVPGAAPEDPDHY

SLRNRGLCLVPVDQTLQLTQSNGADLWAPANTTRRR

>LOC_Os03g55220.1

MVKEEEIITEAAGARGYMEMLGLGEEAADYLMCLSPSSYLSSPAASTTTAVASPTCASYL

APHPYHHLLSFSGQDQYHGDDVFGLQYYGGDQVIPAVVPQKSSPTTECSSSVSSMSSSPT

ATAISSSKSPAFKKKGSRGCDQRKATAPAAATTTNKRPRVRRERLGERIIALQQLVSPFG

KSDTASVLHEALGYIRFLHDQVQVLSSPYLQRLPPSARVPEQERGTPAAEEQPPALRPSD

LRSRGLCLVPISCTEHVAGAGAGTGHGNGADLWSVAAGMAKATATVTAAVERSKEAAAAA

AATAALLRADRPGQQLA

>LOC_Os05g42180.1

MADEHEWWNSPCSARTGDDEAACSTADADESAVGSTPMSFGHGGQPASLSDAAASSSSSS

FLLAGQHMDYWTQDFMGGRAAAAATASFDTLLQLQLQGGDAASRRLLLGDHAAAPPRHLV

VPGAPYGGGGGDDTAAPPRGLSPTPYEAADNLQQQQSFPGGHHVVSNTDRLHDHHQDAGS

PSPATRSSPGSPAAAKKPRIEAPSPMPTFKVRKEKLGDRITALQQLVSPFGKTDTASVLH

EAIEYIKFLHDQVASLSSPYLRCGRPVQLQHQQGSHKVNGNCEGKQLDLRSRGLCLVPVA

STYTVASETATEFWHPTFGGTFR

>LOC_Os04g53990.1

MGDHQMMHAAPAAMYNGGGGTTSSHGVWWSNAVGVPAAATCSTTTELAGYTAWSSALAAG

YDGMVADNGGKQAKSTTTASSESPGNNSSVTFQEPASIPDPAAVAAVPQPGLAGFTDWTQ

PFMNNGAGLHEFLQDGHHDMSASSLMNHSSNNLALQQAGHHHELLSSFGSDLLLSPTSPY

GGFQSSLLRSLMEPTAKQQQQQPALAGLQQYHQYQQQMGHAPAAAAKFAQAVGARDSLQF

TNDAPFWNPSAGFGMPAAVAVAAAAAQDQASVRSAKRSSPAPPRAAATLALKTAMEGVGD

SSSVITKKETAFKKPRLETPSPLPTFKVRKEKLGDRITALQQLVSPFGKTDTASVLHETI

EYIKFLHDQVGALSAPYLKNGAHQVPHLKNSSPDKSKHGEISLKGRGLCLVPISSTFAVA

SEVPVELWTPFGANFIR

>LOC_Os01g57580.1

MADEWWSASQRSHGTSACSAAPSPLTADRVSCGWTSPAAAAAAESTSSITFQDPSRSSAA

HHQPLSDAASSLGDPHMVDWTQAFLSGRSDASFQAVLQDDMAASTRPFRAQPTAADEAVM

TNPFRDMGVGQGLLLDQASAPLHGLSFDAGEPAVAPATHSITTSFGDYQHSASYDAAAAV

MQFSQTPRAPSLPAAAQMQFLSGSYQLPFGGAPPLPSQLLLQAMQPKPSCSSNANTLLAK

VRKEKLGDRITALQQLVSPFGKTDTASVLHEAIEYIKFLHEQVASLSSPYLKNGNPLQHF

QQKGSESTKDAEQPKPDLRSRGLCLVPVASTYTVASETVPEFWHPTFGGTFR

>LOC_Os08g08160.1

MYGGGAAAGGATTTTGSHGGGGGDWWSAAVSSCSAPAPETMQGFGGWSAAVVAVDGGGNT

SRSAAGNTASSESPGSLATGSSITFQEPAGGVADPAAIAVHAQTVAGGGGGGWNQQPFLD

GSGFHGYMSSSRNDHHTNHHHHQINTPSLMSNSSSNNGVMLQEHQHDQNYQFLSNLGFEL

LSSPTSPYGGGGGFRSSLLRSLTEPAAAAKPNDSPGFQQYHHHQPAMNLQPPAAAAGREP

LQFTNSTAAPFWNPSSRFTVAAEGTALGGAGASPAQPTPASLAAKRALEGVGDSSSIITK

KAKADSTPLKKSRTGTPSPLPTTFKACVYIYIIFNLSQMVIDFLPIKFHVRKEKLGDRVT

ALQQLVSPFGKTDTASVLHETIEYIKFLHDQVGALSAPYLKNRQQVPHLKNSTGVDNDGG

GGGGEATAASKRDLTGRGLCLVPISSTFAVASETPVDFWTPFGAAFR

>LOC_Os01g01600.1

MMASSSSSSSLCDNHLLQDDLIPWPSMPFAPAPNTFGLNHQWSQPPMLSSSTDQLSSYEL

ESLQSVESQLAAAAPPTLSPHLQAHQLSTVLMMQELGFQWSSCAAPADQHSIASSTNNNS

NVMMNEEELRPRPDQSLISNPRSCSATTLLPPPHLHLDGAVLPSINVSRLQKLAAGDEPL

QICCKRQAAAAAVVGHSSIRDEHVPCPYAGPPAHLIQGPSNTLQMKRNTNAAAQGRGGRH

GSSTEHRSSTALPPSSKKPRLESHSSSMLPSFKVRKEKLGDRIAALQQLVSPFGKTDTAS

VLMEAIGYIKFLQDQVETLSGPYLRSSKNSKKLACRAAQQQRKGTSNGGDAAAKLDLRSR

GLCLVPLSCTSYVTNENGVWPPPNFRGN

>LOC_Os02g17680.1

MDRGVLHKSSALVGEMGEGHGWWSVNNLRPPFVEQLHNPASLFLPSSTTTSTTPSSSSSP

LHSFSSLLLSNHYPLPSTATTSTAAAPWHDTGSRHGQHLQDSWNHILLGGLASGEEGYNK

NWEGQVLFPTTPAAAAAEADHGSNSYNNIYSTTTTSHGSSTSDDASQLAVAARPSSSPWG

GIHGHHPHHNALQQQASSPRSSCITSTTSLGSNGVLEFSNNTSPRECISTASGAAFKKAR

TQEPSPAQATVKVRKEKLGDRITALHQLVSPFGKTDTASVLLEAIGYIRFLHGQIEALSS

PYLGNGGSSSNGGGGGGGGSNSKLQHQPEASRVQGERNSIFPEDPGQLLHDNAVKKRGQP

DQDESCEEAKTMDLRSRGLCLVPVSCTVDVGVDAGPADYWAAPPAFGIGFGR

>LOC_Os05g14010.1

MNRGEFQSSLVQQMIWSGSSTGTATGVGSGGGAGSLMGSLKPCHEDQEASPNMPSLSSPS

LIFSQQFQHSSPGLVPMNGTAGAAASLPSLHDGGGGGHESSMPESWSQLLLGGLAGDQER

YSATAALLSKGLENWGDHAAAAAASACMVGGMKEEGSMAQAAATAAAAAYSFYGSHLAGD

HQHEIQAAAAGGGASNKSQLSQMLMASSPRSCITTSLGSNMLDFSNTAAPPELRSHHHNS

DNSSECNSTATGSALKKARVQASSSAQSTLKVRKERLGDRITALHQIVSPFGKTDTASVL

QETIGYIRFLLSQIEALSYPYMGDANGTGPMQNGPVGERNPGLFPEYPGQLLNHNGNTGA

QQPAAQPEQQGANDDGKKDLRSRGLCLVPVSCTSHFGGDNAADYWAPAPLGGILR

>LOC_Os01g13000.1

MLSSGGELKSSSLVQQMVWVGTGNSSSSSIMGSLRQLPCSEEQDAASSPASMLFLPQQLL

LHASSNSSPCLNIPEVNLSTGLHPLGSFHGDVQQQEIISGMPDQSWRQLLLGGLVGDHEK

YSVATALLSKGLDDEASMPHEASAAAYDFYGHGGGAGDGILQASPEASSCKSQLSQMLLQ

AAASSPRSCVTTSGLGSSMMDFSNTAAVAPAAEPELTRKHHAGQSDNSSECNSTETGSAL

KKARVQASSSAQSTLKVRKERLGDRITALHQIVSPFGKTDTASVLQETIGYIRFLLGQIE

ALSYPYLGQCCSANPMQQQTGIMAGERSTDGLFPEFPAGQDAEKDGKKQQAKKDDDLRSR

GLCLVPVSCMPHLAADNDVVVGSDFWAAAGGGGGGGAPPLAGMNLR

>LOC_Os04g47810.1

MHGCALHPAAYRRIFHNPRSTRSHGLVAAASPPLHQPAAACGETHRIIQMHALFAVARDT

AFSSSPAADEHLPNEHPGGHLWNQSVLSREEEHGSNDDSFRESLVSLLDNTRDYVMAPEV

FEGVPVACDYLKGMDSDGMAASAVAAYSLDNNGQHASVSSIEHGIASSPLLAYQLGKNSA

VVQRSIQQQEVGSPMAAFLQQLIPTSVLDQSGIGFGGVCLDGSALEASFCMRTSPDVSSF

SGHRSATAEELMSTDTREQEITRLARSCSSSGSDRNKKKLSEVRGGGKAKKFKSETSHST

SSPKHQSPKVKLGEKITALQQIVSPFGKTDTASVLLETITYIKFLHEQIQLFSQPYMTNS

TNKGHIHWGGEGKRKAGLEHDLRGRGLCLVPVSWTSQEYCDSILPECWAPAYRNYFYR

>LOC_Os02g45010.1

MRLHAGAVQLQYFMPQQGATAAAAADHHQADSATACSASTSPAAAAATMWEYHHQLSTHA

ALQPSSSFPYSYWSPYSGSTALAGSAFAADSSSSSTDVMRLPAAGEHAHGHGWSHGELSN

STTGGGYRENFLDLLASKNVTPEMFEEVPASEHYNVAPAGTTLTTTARSFDHHARSDVSP

IKYEIAGSPLYLGGTNTVLQVQDMTMMSSTPACYGEHHHHHHHQLTKEGSCNHQQQEQHE

LAISPMASFLQQISSGSASVGVHNSSLDYSGLGDQPDKICCQDGREMEASPFGMRSLPDL

GSFAGYTPAIESTSVQPYMRCANSSDSNRQEQETVPARSSSSGSGAAATDRKKRKSEERQ

ESTVKKSKQEASKASPPKQPVPKVKLGEKITALQQIVSPFGKTDTASVLFETIKYIKFLH

EQVQLLSEPYTNSSRSNKQGNSVPWGDQAEASKGETMEHDLRNRGLCLVPVSWTPEVYRD

GNAMDYWTPAYRGCLYR

>LOC_Os07g28890.1

MARGAAGGEVGRSMPAVHDAHDDSENFLELLNSRTLAPELFAEPPACDYLKKMEYGGGGG

GGGGGWPEHQFTAAAALEKHLSSAAAAGYGGALAHHHHAAGAPERLTANLSDLVSNWSIA

PPNHGHHVGGAAACDNPAVAAAMAAAHGGGNVKQSGSSFLDSGGGGGGAMLQQESSSSTG

TGGGGQDFLRPMGLAAGSSSYSSMLGLSSRMYGGGGTATMDVPWGSSNAGAARSLSDLIS

FGGGAMDKPPPPPPSSAPARTSSADYKKQQGQQEISSPVKTSSSGGGGKEGKKKRSEEAA

GSEGSTKKSKHEATSPTSSLKSQVPKVKLGDKITALQQIVSPFGKTDTASVLYEAINYIK

WLHEQVQLLSDPYMKSSSSKDYNAWGGLDRKEKADAEVDLRSRGLCLVPVSCTPQVYRDN

NGPDYWTPPYRSCLYR

>LOC_Os03g17130.1

MVIASNKHELMKYLSLTLERFRLQKQQLCRWIRHDDLDMLTDELERKRYRTLILMADTFL

ANDLRQFPSAKRKASLYTSTLANIFILMLKGSTGIQLLSLFSGYMKDDVSSAVETQLAES

CRLESGVETCRLLVSSVAVKCEASNSPSTTVGFDRMIAADYDLFHHMSFSPSLQNLQSPT

FFTTRSSESYLGESSIYGGGARPALAQFSYSQPIAATSAAHLVRWTAAGEPMTGDGGFRS

SKRLKTATTATTQPPRHGVKCHAKPRNQTTKATCKKRSQKLGDRITALQQLVSPYGKTDT

ASVLHEAAACIRQLHQQIQILTAPYPGTSSSSASSQQQDAGGGGGTATELRRRGLCVAAL

SPAVVSLAAEGGRRRTDVEDQKRIWFSNQ

>LOC_Os02g47660.1

MAQCGGGDVSRHRKGHLDTVESLCQGLLDDVMLDDDKCRAMFGYLQEWQDLASMCYGSLG

GEPPLAPEASNGSGSSGGGGSFRKRRPDDAKGESNSICKRQRGKQQQQQQPCHPDQMAAA

VGKGRPERARPGAKKKAEVASPKDSPATSASTVTAGQKTDYIHVRARRGQATDSHSLAER

VRRERISERMRYLQELVPGCNKVTGKAGMLDEIINYVQSLQKQVEFLSMKIAASNPVVNF

NIVEDLFGRQLSQAACNPAALPAMALPMAQVEPSCLQMSPLQQMQTSAGSSGYGLEMVVS

NQYSPPGGPMSVPAGASVEPCLNVNGAAGWDIGSHGLFSGFDAPFQSVQSDCLLDNLKME

M

>LOC_Os08g42470.1

MADFSPHHSLLLKATAAGAAIATTNDPNISSFFLYNHSHGSQAPQPANAAAAAIVEDASL

ESSVSAVLDTSPSVDRKRKAAEDSAHSKDSCKDGKSRRGKKASKEVEEKSTTEDEPPKGY

IHVRARRGQATDSHSLAERVRRERISERMRMLQALVPGCDKVTGKALILDEIINYVQSLQ

NQVEFLSMRIASMSPVLYGFGMDSDGLHDQKIGGMFQEALAMPNPVLNQSSPAPSQAIMD

TTSTTSYSLQSQHGAISFSQDNGSYLMQAVGEPRQQEMLNQLVFNNMCSFQ

>LOC_Os09g33580.2

MAGFSHLPQQMEHGLITNNGFLFCHGSHGGAATTTAPAIPEDASMETSSVVLDTSPQDKK

RKPREEDTASLNSAHSKEAKENGRKRGGKKHSRDQMEEEAPQGFIHVRARRGQATDSHSL

AERVRRERISERMRMLQALVPGCDKVTGKALILDEIINYVQSLQNQVEFLSMRIASLSPV

LYGFGIDSDAFSDHSQKMEGMFHEAVAIPASVLNRGSSPAQSHAIMDTSNTSPTPYTLQV

QGGSNNNSLSQDNGSYIMQTVGEPRQELFNQVVLNNYMCSFQ

>LOC_Os09g33580.2

MAGFSHLPQQMEHGLITNNGFLFCHGSHGGAATTTAPAIPEDASMETSSVVLDTSPQDKK

RKPREEDTASLNSAHSKEAKENGRKRGGKKHSRDQMEEEAPQGFIHVRARRGQATDSHSL

AERVRRERISERMRMLQALVPGCDKVTGKALILDEIINYVQSLQNQVEFLSMRIASLSPV

LYGFGIDSDAFSDHSQKMEGMFHEAVAIPASVLNRGSSPAQSHAIMDTSNTSPTPYTLQV

QGGSNNNSLSQDNGSYIMQTVGEPRQELFNQVVLNNYMCSFQ

>LOC_Os05g01256.2

MESLCGQLEHEHEQQLHQSSSSSLLPYCFLSAPEPLPVPGFPAGCSNLEEKAAAAMAAYE

YESSSCSSLDPTSMPMVYSPIVLQPQECPLSFVFDNAAAAAGDNKWVPGIQGSCPCSLGS

TQDMDASWGKSRKHKRSNVGLKGLEEKKARRVVLHQHDDDVKKKAKEAAGGEPPAGYIHV

RARRGQATDSHSLAERVRREKISERMKMLQSLVPGCDKVTGKALMLDEIISYVQSLQNQV

EFLSMKLASLSPLMYEFGPGIDMHPDVLRQLAKMPHEMVQCMGQMGSTGISLQGLGGGPT

GFAQDGSSHINMVVMQVGEQGQQQGSLHQVEMSSHCFFH

>LOC_Os03g51910.1

MYSGQQSDQCPGPNSGKEFLEVNWDSVALHQKMGYNSGAFGFQAYPMVLEDREGLYRSPN

GTFCQNIQLSDDHSSGAKRRKGIDDHIALLNPSASSRIQNVGDQQTEVSSQQERISMEED

NQKSCSKMQSKEDSSDGDGTKEDYVHVRAKRGQATNSHSLAERLRRKKISERMKLLQDLV

PGCSKITGKAVMLDEIINYVQSLQRQVEFLSMKLATVNPELSFDIEQILSKQMMLSQDRH

LAFYGVDPGSSALVAHFNQGIMQPEMLCNVSNPADVLQGTTIQDISTVNQIPAMWEGLQN

IPHLNYNPGGAMAEGSTNNSGSMKIEK

>LOC_Os09g29830.1

MLCSAGYYYALGIRSLEPRLGDSMDMSESSEKGMESNASSGPGNGIPVEWQSQFSSAFAC

QPSVAAQHQQHAMMDSFAAASAGLWASSDVVSAMSSAAPPRGAGFLAPVPGFLQQGLGHF

PVDSGFIERAARSTCFGGGMMAGGPYGAADQAMGDAFGGTAEGLMDHHRNVGNDKAEEFA

GNGHDEVPSSEVAGGDCSSKGSDSKKRRRPNEVMGTDQVHSSNLPSDSANESVHSKDKGE

ESSPATTNGGKSKGKGAKETSESQKEEYIHVRARRGQATNSHSLAERLRREKISERMKLL

QDLVPGCSKVTGKAVMLDEIINYVQSLQRQVEFLSMKLATVNPRLDLNIEGLLSKDLLRF

PGVPSSSIGFSPEMMHPQLQLSQPGLIHGGTAGMANPDVFRRIIQAQLGAKDGSQMPHSL

NGSFSDVSQMAYPSLGSQDLSIRPSQDGFQM

>LOC_Os06g16400.1

MNEKDATDLEERSEASEHGQALSFHGGAMFLQEAQIASPAAANNALTSMANPFPIPPGLW

NPPSHNMGLGETSFSSLLGMLSAGAPPPFVATPGFVDSTAGFPCYNGGNLGAMINHPFPG

IHQPLGDFQNGVEPCREIEDIEIEGSKNVSQTGEKQQGDGETTHAVDSSSKELSMPGRNG

GAGHDEGTRVSCSKKRKRSGQDGGVKHAEGGEQLATVGSAQKNEDDEKGEPKRSSVASGK

SSGKQIKDNAGSPKEDYIHVRARRGQATNSHSLAERVRREKISERMKYLQDLVPGCSKVT

GKAVMLDEIINYVQSLQRQVEFLSMKLASVNPTLDFNIERILSKDIFQCRGTTASSAFGF

FPDIVHPRLHPPKYTQVGMPSIVNPTDAFGRVIHAPLGTNSAFKEPKHQMPNNLNGEFQD

VIEMPFTHDHHGSNDQP

>LOC_Os08g38080.1

MDANGGNGFGVESNASAVHGGMAMAWQWHGGQMSGGSSCAAAPPPVQQPAMDSLAWSSTV

SPSTGAATAASGAGFLLPPAAVRGGFGSFPVNSGGIVEPAGDCSSESKKRRSDEIAGTDH

ANASNALADSGNETECSKDVNGEVIGPPATAAAGGKSKGKGAKDAGEAQKEGYSHVRARK

GQATNNHSLAERLRREKISERMKLLQDLVPGCSKVTGKALMLDEIINYVQSLQRQVEFLS

MKLSAVNPRIDLDIESLVNNSKDVLRFPGQPSSAPMGFSFSTEMMPGLQLSRPGILQGGV

HGMINPDVFTSLMQKQQQNDKGAFREPQMHQTLDGSFRNTAQMPYPQVMSSEELSIRQDQ

DGFHM

>LOC_Os03g12940.1

MKLRLRSMDQRGGAGGAAETHRVQLPDTATLSDVKAFLATKLSAAQPVPAESVRLTLNRS

EELLTPDPSATLPALGLASGDLLYFTLSPLPSPSPPPQPQPQAQPLPRNPNPDVPSIAGA

ADPTKSPVESGSSSSMPQALCTNPGLPVASDPHHPPPDVVMAEAFAVIKSKSSLVVGDTK

REMENVGGADGTVICRLVVALHAALLDAGFLYANPVGSCLQLPQNWASGSFVPVSMKYTL

PELVEALPVVEEGMVAVLNYSLMGNFMMVYGHVPGATSGVRRLCLELPELAPLLYLDSDE

VSTAEEREIHELWRVLKDEMCLPLMISLCQLNNLSLPPCLMALPGDVKAKVLEFVPGVDL

ARVQCTCKELRDLAADDNLWKKKCEMEFNTQDTCGCMMCKCIYSDQRKDIVLADKYTCGN

YMQKPVTQPGRWLIILVYHSLLCQYITIGLSLLWYHLVDLVQDAPAAGIHFDCIIPLPIN

PYQLPPSAGACCSTTQASASAKDGGNMYSPPCSAAASSQGHCFAVGANQLASLDLAMDFD

EPILFPVHNASLQEGIQFYNPTGDTQLSRNMSIDKCLKGSKRKGSGEGSSSLHSQEETGE

MPQRELSMEHAGEKAGDADASREEYVHVRAKRGQATNSHSLAERFRREKINERMKLLQDL

VPGCNKITGKAMMLDEIINYVQSLQRQVEFLSMKLSTISPELNSDLDLQDILCSQDARSA

FLGCSPQLSNAHPNLYRAAQQCLSPPGLYGSVCVPNPADVHLARAGHLASFPQQRGLIWN

EELRNIAPAGFASDAAGTSSLENSDSMKVE

>LOC_Os03g58830.1

MDPAPTLAAELWRTPYLGGGGGGGGGGRGLEAAASGVTEQSNGSRGGGGGGGAGRRRQRE

APALEDDSSRIVSTSGGGGGGQDLTDSEAKRFKASKSSGDNSSLRTEAETDSRNASKSGD

QNPPPPEPPKQDYIHVRARRGQATDSHSLAERARREKISERMKILQDLVPGCNKVIGKAS

VLDEIINYIQALQRQVEFLSMKLEAVNAHVNNGIEAFPPKDFGAQVYNTAPGLTFDPQTP

REYAQGSTPSEWLHMQIGGTYERVT

>LOC_Os01g68700.1

MNCGPPDQLPPATAPSCFLNLNWDQSMDAAAGGHLDPALSSMVSSPASNSTGALHGISPQ

PHYGGGTPLSSPPKLNLSMMGQFHHYAAPPQVGGGGGGGGGLPILENLMPMGHLDQFLAD

PGFAERAARLSGFDARGGGGGGGYGGAGPAQFGLPDAGAAGASKEMELGNTRDESSVSDP

APGGAEIPPKGASDGNARKRKASGKGKGKDSPMSTSAAKEDSSGKRCKSTEESNAAAEEN

SGKGKAAQSNSENGGGKKQGKDSSSKPPEPPKDYIHVRARRGEATDSHSLAERVRREKIS

QRMKLLQDLVPGCNKVVGKAVMLDEIINYVQSLQRQVEFLSMKLATVNPQLDFNNLPNLL

AKDMHQSCSPLQSSHFPLETSGAPLPYINQPQQGNPLGCGLTNGMDNQGSMHPLDPAFCR

PMGSHHPFLNGVSDAASQVGAFWQDDLQSVVQMDMGQSQEIATSSNSYNGRIVANSPHEN

GALT

>LOC_Os08g41320.1

MDGGRVGGDYISSLLSSSPRLDFGVPVLDAIVAPGGGGGGGDCGLDKLCGDPGFAERAAR

LSSFNNGGGGVGQRYGGAGAGLFGMPPPAPGDFAGGGSREASSVSDPASSAMKDAAANAK

KRKSTAAAAAAAKGKGKEPPVGEEKESDGKRCKTGNGEKESSVKPKAEQAGSDSSVEDGG

GGGQKQGKGKNAKPVEPPKDYVHVRARRGQATDSHSLAERVRRERISQRMKVLQDLVPGC

NKVIGKALMLDEIINYVQSLQRQVEFLSMKLATVNPLDFSNLPTLLQKDMFQACGPSASS

VFSLESSNSAFRFAEQGDVFQQFAQNSMESQCTLNQLDLALSQATNAAQYAFQDGTAGAN

LQQRNFWEDDLQSVFHIENGQSQENGVSAPNFHGQQQAGHMKMEF

>LOC_Os09g32510.1

MDSDYVAGLLMSAAAAGLDLGVLDGGGGAFLETLCGGPGFAERAARLCGGGAGLFGLPAV

GNAERGGCSREGSSVSDPAWAHATGGGGDNARKRKAPASAAAGKDKDAVVGGGSSPCEVG

EAKAPDSKKCKAEVNPKVEEAASDGSVGDRVQKQGKGKNSSKPAAEPPKDYVHVRARRGQ

ATDSHSLAERVRREKISQRMKVLQDLVPGCNKVVGKALMLDEIINYVQSLQQQVEFLSMK

LATVNPQLDFGNLSTLLQKDMFQSCGPSVNSVFPLESAGTAFPFCDQADFFQSFGLGAME

NQCSLDLANTALPHTGSTQYAFQKQVIAALKNLQSTEQMVLTCIYDDNTVCLWFSFQQRD

LWEDNTFQYNDEQSQEDAVSAPNFDGQLQAADHTEIEF

>LOC_Os04g28280.1

MNPTTAAAADQPSKPSAAAAARKRKSSAKPKASSSSLPTATATTNASPKRSKVAAGAGDD

GDADADAAEEKPEPAKDYIHVRARRGQATDSHSLAERVRRERISERMKLLQSLVPGCNKI

TGKALMLDEIINYVQSLQRQVEFLSMKLATMNPQLDFDSHYMPSKDMSHMPVPAYPSSDP

TTTTAFSYTGSPATADPFTVYNCWELDLHTAMQMGATTGLSQDGPIATMAPSPSPLPHHP

PLHGFYGGQQQQGTTVNHMKAEP

>LOC_Os07g09590.1

MDPAPSLAAELWRPHHHRHHFEASSVVTDQGSGSRGGGGSGRRRPRSDAGPEDDDLSKVV

STSAASGGGGGGGQDSDAPEAKRLKPMKSSDKNDSLRTEAGTDSGNSSKAADKNATPPEP

PKQDYIHVRARRGQATDSHSLAERARREKISERMKILQDLVPGCNKVIGKASVLDEIINY

IQSLQHQVEFLSMKLEAVNSHMINGIVAFPSKDFGAQPYNTAAGLTFDPQTTREFAQGST

SEWLHMQIGNAYERVT

>LOC_Os06g41060.1

MELLKELVPGCSKVSGTALVLDEIINHVQSLQRQVEYLSMRLAAVNPRVDFGGLDNFLTT

ECGRITGLNYKNGMDLEQVTWPDMGVHGARNLMQLQQQFWHGDLAHPLQPPSQWEKRTDT

NPPVFSNSSSSLFGYDLASSGAPAQTGNKLKTEL

>LOC_Os06g09370.2

MDYSAGSYMWPGNSGSENYNFVDGSSESYAEEGSLPPSGYFMGAGSDRSLKITENERNPT

MLANGCLPYNTQAHPLSGQILPKGELPNNLLDLQQLQNSSNLRSNSIPPGVLQCNSTSGT

FDAKLDTPGLAELPHALSSSIDSNGSDISAFLADVHAVSSAPTLCSAFQNVSSFMEPVNL

DAFGFQGAQNVAMLNKTSLPNGNPSLFDNAAIASLHDSKEFLNGGSIPSFGTVLQALGAG

GLKAAQQEQNIRNIPLPTFTSGSHLAVTDAQGPPLPSKIPPLIHDHNSEYPINHSSDVEP

QANSAPGNSANAKPRTRARRGQATDPHSIAERLRREKISERMKNLQVLVPNSNKADKASM

LDEIIDYVKFLQLQVKVLSMSRLGAPGAVLPLLRESQTECHSNPSLSASTISQGPPDMPD

SEDSSAFEQEVVKLMETSIISAMQYLQNKGLCLMPIALASAISNQKGMAAAAAIPPEK

>LOC_Os02g35660.1

MGGFAYPFTPSPAWSRDAVFAGSPWAAGGVSSLADALVSYGAVDDEEAAFLGKTAASSPS

TARLHEQQQLLLEAELLRHGDGLGFAAMDDDGGAAMLGALEPCAMPLTDSGGPPVICSSS

SNDSSGSEHSAAMPAGGGFLVGEQQQHVPPAAYAAGGVLPSMAAGEETPQSFGFGSLFNG

DLLQEATVSKYHHHQQQQQLGVVPSSQPHHLNDDIDFNTGKLMSFASGQQHVTPSIDSLQ

IDQKEFSSGLHHLNLSSLISGPLASFNATQSHRQPAEACGGKNGGAAPFVNLSEVLPKGN

GSGSAGNGAPKPRVRARRGQATDPHSIAERLRREKISDRMKDLQELVPNSNKTNKASMLD

EIIDYVKFLQLQVKVLSMSRLGAAEAVVPLLTETQTESPGFLLSPRSSSGERQAGAGAVT

GGLPGDQPELLDGGAMFEQEVVKLMEDNMTTAMQYLQSKGLCLMPVALASAISAQKGTSS

AAVRPEKKKNGDGDGGGDEEDVKGEFDAPRRPPVGRPKEMRSRV

>LOC_Os03g58330.1

MAGQQPQQQGPPEDDFFDQFFSLTSSFPGAAPGGRAAGDQPFSLALSLDAAAAAEASGSG

KRLGVGDDAEGGGSKADRETVQLTGLFPPVFGGGGVQPPNLRPTPPTQVFHPQQSKQGGA

AVGPQPPAPRPKVRARRGQATDPHSIAERLRRERIAERMRALQELVPNTNKTDRAAMLDE

ILDYVKFLRLQVKVLSMSRLGGAGAVAQLVADIPLSVKGEASDSGGNQQIWEKWSTDGTE

RQVAKLMEEDIGAAMQFLQSKALCMMPISLAMAIYDTQQTQDGQPVKHEPNTPS

>LOC_Os07g08440.1

MRALQDLVPNTNKTDRAAMLDEILDYVKFLRLQVKVLSMSRLGGAGAVAQLVADIPISVK

GEASDSGSKQQIWEKWSTDGTEKQVAKLMEEDIGAAMQFLQSKALCMMPISLAMAIYDTQ

HSQDGHSVKPEPNTPS

>LOC_Os09g25040.1

MAAAAAAGQISLDDLRNGGGVAANAGGGGGVHDDFLDQMLSSLPPSAWPDLAAGKAAEDD

AEGMHHHHHQQQQQFGGPYDESAMLASRLRQHQISGGGGGGGGGAAAVKQMVLQQLADLR

QGHHMMLQGLGGRSPAGGGGGGGDGGLLLPLTLGSGGSGGDVQALLKAAAANSAGGGDAG

GVYGGGFAGSLHQQQQHFQPHPQTAPTIPTQSFGGGGGGGGGGTASGGGAAQPQAGAAGG

GAPAPPRQRVRARRGQATDPHSIAERLRRERIAERMKALQELVPNANKLMQTDKASMLDE

IIDYVKFLQLQVKASTYTKLLIHVLSMSRLGGAAAVAPLVADMSSEGRGGGAANGGAPAA

AGSDSLTVTEQQVAKLMEEDMGTAMQYLQGKGLCLMPISLASAISSATCHLRPPVVAAAQ

QFPAGLGAAAAAAHHHQLSAAAAAAAMRGHLPGLNADGSVPASPSMSVLTAQSAMANGGG

GAADGEGSQLKDAASVSKP

>LOC_Os04g52770.1

MEARRPTPTRRSRSAEFHNFSERRRRDRINEKLKALQELLPNCTKTDKVSMLDEAIDYLK

SLQLQLQMLVMGKGMAPVVPPELQQYMHYITADPSQIPPIRPSEPRPFQITHATQQRQSN

VESDFLSQMQNLHPSEPPQNFLRPPKLQLYTPEQQRRGLASSSGHNSGWITERNSSYNFL

E

>LOC_Os12g41650.2

MNQFVPDWNTTSMGDGFAPLGEDDGLVELLWCNGHVVMQSQAPRKPPRPEKTTAAAAAAM

AEDESASWFQYPVDDVLEKDLFTELFGEMTAAGGGGGDVRRAACKEERGAVAAFQSRMMP

PPWPARGKAEFGDVDDVCGVSEVVMAKMDGAAAAETVGESSMLTIGSSICGSNHVQTPPV

GNGKAGAGTAGAARRAHDTATVASSSMRSRSCTAKAEPRDVAAAGVGGKRKQRGGAAMES

GSPSEDVEFESAAATCSPAQKTTTAKRRRAAEVHNLSERRRRDRINEKMKALQELIPHCN

KTDKASMLDEAIEYLKSLQLQLQMMWMGGGMAPPAVMFPAAGVHQYMQRMGAVGMGPPHM

ASLPRMPPFMAPPPAAVQSSPVVSMADPYARCLAVDHLQPPPPMHYLQGMSFYQLAAAKN

LQQQQNTAEAPPPPPAGGNRAAADS

>LOC_Os03g43810.1

MNQFVPDWSNMGDASRTLGEDDNLIELLWCNGHVVMQSQNHHRKLPPRPPEKAAAAAVQE

DEAGLWFPFALADSLEKDIFSDLFYEAPVAATAEAAPAGPGAGADGEGKTCKGDAAMAEE

ERGGPGAASEAPRELMPPPKSTNASCSRQQTMSLADGGDNAGDLSELVRARRSSGGAARR

KAEAGGGGGGASSSMLSAIGSSICGSNQVQVQQRTASEPGRRGAPPSAVGSANAIPCGGR

DHGHGHEATTVASSSGRSNCCFGTTTTTEPTSTSNRSSKRKRLDTTEDSESPSEDAESES

AALARKPPAKMTTARRSRAAEVHNLSERRRRDRINEKMRALQELIPHCNKTDKASMLDEA

IEYLKSLQLQLQMMWMGSGMAPPVMFPGVHQYLPRMGVGMGAAAAAMPRMPFMAAPQPVV

PTPPVNHLDLGVNHLQPPPTQGVGYYPLGAKAVQQQQNPPLHVPNGSIMPPPENAPNTGS

GMGSFYFYFYFSSD

>LOC_Os07g05010.2

MDAGATARSSSSSAMMMNQKKPLLSDGELVELLWQDGGVVAHAQTRHRSSDVLARSGVTG

EEETASAWFADGGGGGGGDDDALGLGMGRDIYSQLWHSFANVDGHAAGALALATPTPTPR

AAARSDDVSSRLDEADLSICGSNAVVAPALPADDDDDIDAAAPREEEEEEEEGPGAARAA

GASSSGGSGSGSGSYPLFKRGREELVDSLSEVADETRPSKRPAAKRRTRAAEVHNLSERR

RRDRINEKLRALQELVPHCNKTDKASILDEAIEYLKSLQMQVQIMWMTTGIVPMMFPGTH

QLMPPMGMGLNTACMPGAQAQGLNQMQRTTYYMNNSLPNQMPQIPSPAMNAPSVPDDMQN

DNRIRGPRNPFLHCNDTLTATAQVPGLFTYGSQIAEQNEIQELLSGAVIPSSSDGTIK

>LOC_Os05g04740.2

MLRGNDTGSDLAELLWDNGAPAPLRPPPPPPFQPFTCSAAATTSPPAHDYLFIKNLMRGG

GAANHHHHDDDDDDDDDVPWLHYHPVVDDDDDADADTAPLPPDYCAALLSGLSDHLPPPA

AAASRVDPDPCSSSHGAVVPSTSAAAAKQARTSGGGGGGVMNFTFFSRPLQQRPSGGETA

SASASAAATSTVPVESTVVQAATNRLRSTPLFSDQRMAWLHPPKPSPRAAAPPPPPPLAP

TTRHRLDTAAATATVAQRLPPSEARAPDAPPPAATATATTSSVCSGNGDRRQLNWRDSHN

NQSAEWSASQDELDLDDELAGVHRRSAARSSKRSRTAEVHNLSERVSTHTMHHPQHPRRV

MTSSHLAIELLSSSQRRRDRINEKMRALQELIPNCNKIDKASMLEEAIEYLKTLQLQVQM

MSMGTGMFVPPMMLPAAAAAMQHHHMQMQQMAGPMAAAAHFPHLGAAAAMGLAGFGMPAA

AQFPCPMFPAAPPMSMFAPPPPPPPFPHAAATAVEQTPSPPGAADAGNAPAVKQA

>LOC_Os02g56140.1

MMDGRGSQEEEHLDLIMRHHASMGLDRCESEEALGSSESEQPTRPARPRGKRSRAAEVHN

LSEKRRRSRINEKMKALQSLIPNSSKTDKASMLDDAIEYLKQLQLQVQMLSMRNGLYLPP

VNLSGAPEHLPIPQMSAALDQNSAKASDPSVVLQPVNQTSGALLPFELASQHKPLFLPGV

PNATALEPRFLVESSRSNLQSLRFTEPAEMIYPDEMMLKHRLTSASESTIVPGTDEKSVR

QNTYMMNADRFDRYALSKDQLQHIMPKNTESVLDMPHLQR

>LOC_Os06g06900.1

MDEQRGRGGFDELVLLHQQQEQRRRREQQQEEEEEEEVRRQMFGAVVGGLAAFPAAAAAL

GQQQVDCGGELGGFCDSEAGGSSEPEAAAGARPRGGSGSKRSRAAEVHNLSEKRRRSKIN

EKMKALQSLIPNSNKTDKASMLDEAIEYLKQLQLQVQMLSMRNGVYLNPSYLSGALEPAQ

ASQMFAALGGNNVTVVHPGTVMPPVNQSSGAHHLFDPLNSPPQNQPQSLILPSVPSTAIP

EPPFHLESSQSHLRQFQLPGSSEMVFHGEIMPKHHLSSHQESLPGNEMNSIRKESSMLNT

NNFDGVSLSKEQS

>LOC_Os01g67480.1

MNRMTAPHGGMPPPPMPAAGGLARYGSAPGSLLASIADSVIRGRGVGVVDQLHHHQHQHQ

LPPPPPPQQQQMVGRYFSAESSGLTSCESSCRTTTTTSTAAAADVGRHPLERAYGGSGEI

HVDASSAAVPLFRHSSSPAGLLSRLMADPHGNGMAATRGMGGYSGGGGDAGAMAHRRLSS

QWSFSRQDLPQISEMGGLIPDIGESIVTGGGGNSSSDGAGHGAQSSSFLSSRNFSMSSWD

DTNSIMFSPPSSSKKARVAAAAAGDHGDDMVSSFSNIDSQFGLSKQSSLEMAGMDDFLQL

QPDSVACRARAKRGCATHPRSIAERERRTRISKRLKKLQDLVPNMDKQTNTSDMLDIAVT

YIKELQGQVEKLKHDQANCTCSGKHDC

>LOC_Os02g39140.1

MMRRFLPVGGGGGGGVEPSSSSTTPQRGEAEAAGLRFGGGDISLGPHGGGGGGGGGGGGH

GHQLQDGSVDLLARHSSSPAGFFSNLMASNGFPGSKGGGGSGAEAHHHPSMAGSGSGSSS

GGRKMKSQLSFTAGPPHLSHIAEDGAFPDRAGAEASVPRTFSAGGSSGGGGFSIVGPWEE

SRDIISTLGGYESQFGGMASTSALEMAGMDRYLQLQHDQVPFKVRAKRGCATHPRSIAER

ERRTRISEKLRKLQELVPNMDKQTSTADMLDLAVEHIKGLQSQLQALKHEQEKCTCCSRP

>LOC_Os04g41229.1

MNPAPSRAPQRQQRGGEMSARYGGGLQFFADAPPAGVEGGAATARTFFPVPGGGGEQQPP

ERAMRQQHYGGGGSGAAEISLGHGHGHGGKHHFHQFGVEAKDGGGGGDQSGFLTRHNSSP

PGFFSSPVMDNGFSSSARPAGSSLGEVRHGAMSSSSNNNKKMKAPLSFASSRQGSGGLSQ

ISEDGIPDLTDSIHGAAHHHGRSEENVSTHDHVVRSFSSGGFSIGSWEDSNSIVFSTSTG

KSGAHGNDDIIATLSNYESQLVAPREMAGVEKYLQMQHDQVPFRVRAKRGCATHPRSIAE

RERRTRISEKLRKLQALVPNMDKQTSTSDMLDLAVDHIKGLQSQLQTLKEDKEKCTCSCK

QASRNRPAD

>LOC_Os08g39630.1

MYGSPVSKDLNLPVQPPAMSSSGLLRYRSAPSTLLAEFCDDFLPPAAAPRAASPDADNVF

SRFLADHQIRDKSPPATAAAAAAAAHFPDDPTMATQHHHQQQMMFQHHPQQMASVEGLYR

TVSSTGIDAATAAANAAGGGGGGLLRQSSSPAGFLNHLNMDNGYGSMLRAGMAAAGGGVG

FRNGANAAAAADSPGGSGGRLKGQLSFSSRQGSLMSQISEMDSEELGGSSPEGAGGGGGG

GGRGYLSGYPMSSGWEESSLMSDTNISGVKRQRDSSEPSQNGGGGGGLAHQFSLPKTSSE

MAAIEKFLQFQDAVPCKIRAKRGCATHPRSIAERVRRTRISERIRKLQELVPNMDKQTNT

ADMLDLAVDYIKDLQKQVKGLNDSRANCTCSAKHQQYSG

>LOC_Os10g40740.1

MNQCVPSWDLDDPVGGGGIGGGGGGGHRVVSGGGGGFMPVAVPTSDQYNEVAELTWEKGN

ISSHGLLLNRPAPPKFPPHQQLQAAMGGGGGGGVVGDRETLEAVVGEAAARSSSSSHLAA

RARPVPAPWLGSVGVVAAADALVPCDADAAEGRSKRPREVVGEDGRRACASQGSAAPGRR

GESTLLTLDACCGTAADDVCGFTTTTNNSTSLEDRTEDKGSPETENTSIAGGASDSRCFS

RRSQSQRGGMCDEDEHVVIRGEGAMRSSISTKRSRAAAIHNESERKRRDRINQKMKTLQK

LVPNSSKTDKASMLDEVIDYLKQLQAQVQVMSRMGSMMMPMGMAMPQLQMSVMAQMAQMA

QIGLSMMNMGQAGGYAPMHMHTPPFLPVSWDAAASSSSAAAADRPPQPTGAATSDAFSAF

LASQAAQQNAQQPNGMEAYNRMMAMYQKLNHQQQQQQDQPSNSRQ

>LOC_Os02g55250.1

MQPNARQMRGGGGGGGGGQDDFFDQMLSTLPAVWSELGSGKPAWDLTAGAVGGGGGASDD

HSAAAFDDSALLASRLRQHQIDGGGDKPIMLQLSDLHRHHGLAAGDDSGGAAGFLPLSLF

ADRSQDDIDAAFKSPNGARGDHALYNGFGAAGMHGAAAMQPPPFGQGGSMPAQSFGGGAA

ASGGGGGGSASAAAAAGASSGGGAAAPPRQRQRARRGQATDPHSIAERVYHSPTTFPFSP

PFFIASMPCCLRRERIAERMKALQELVPNANKTDKASMLDEIIDYVKFLQLQVKVLSMSR

LGGASAVAPLVANMSSESNGNGNATSSSGNGEAANGSSNGDNNGGGTLRVTEQQVAKLME

EDMGSAMQYLQGKGLCLMPISLATAISSATSSSLLPRTGGGAGGSLHEGGNGTSPPLVNG

TATGCDDAGVFFSVKFVVELSFLLLNEDCRGKEESKLLVQKGP

>LOC_Os06g08500.1

MQPSSRDTVAGGGGEGTQDDFFDQMLSTLPSAWADLGGGGGGAAGKSPWEVDPAAAAAAS

QVFDESALLASRLRHHQIGGAGGGGGEKPVMLQLSELHRQAGGGEEDGSGAFSPLPLFTD

RTNVPPREEMEGGFKSPNAAAGGEHALFNGFGVHGGSGGAGQPPFGQLRRERIAERMKSL

QELVPNANKTDKASMLDEIIDYVKFLQLQVKVLSMSRLGGAAGMAPLVASMSSEGNSNGS

SNGGGGKASKGGTGGEGGGGGGGGGGGGTGGGMRVTEQQVAKMMEEDMGTAMQYLQGKGL

CLMPISLASAISSATSSASLLSRPSIRHAGAPPQTMLDAAGPTSPAAMSNGDDPRHAKAD

GGAGGTQ

>LOC_Os12g40730.1

MWQGGGGGGASSFDQPPPPVSYHRLSPPPPAMGTTTTTLLPEQPLVDDHHLPAAAAAPPS

EEEMAAWLYPIVSGHEVAGGGWRSPEAQDDRRAAPAPEKKQMEKMPAAASPTTTMNKDET

SDDSGERKKKKASSAAGKSKQASPRGCRSSQPYRKSGDSIDELFTKFHRRRFKITERFRT

LQRLVPGCDKSNQASTLDQTIQYMKSLQHQLKAMSVVGSPPALLYPAAVHPQSYMHPPPP

PPPVTMPMHPGMVLAAPPPGAAPPPGPPAMVPFGAMLPYPPYPAVLLPPPAAATLYGRPP

APAPGVAARRHGSSGGGRISKSSSSSLCKKL

>LOC_Os01g38610.1

MDRGFNEMILSESVWNGGGGGGDGDGGAVVLPPEVGGVNAVDGSGMTMLERLVLDEALAA

AILELQGIQAPGCGGGGGKVAVVPPPTAGDGGVEAAVAFAAMATGTPAYADVDADVLQRQ

RQHHHRHQGAMGMAAEYDVAPATPAVTLSAVPPPPPPPAFATAAASVDGGGAMDATVFSG

IGNDDVVDAVSATVAMTTATATTSQCERVRGGGGGGGCGRKQRRPGRKRKAAEPTAAAAA

ADMSSQDNPLCSLLASNTAGADGGIQIAFSTSAPASKRAKPSLSSSSSSISFDGRGPGGN

CGGGGGDDPLYEPDTEALAQVKEMIYRAAAMRPVTLGAEDAGERPRRRNVRISSDPQTVA

ARQRRERISERLRVLQKLVPGGAKMDTASMLDEAANYLRFLKSQIRELQTLDRRNYPNAA

MSINTAAATTMATSSSPTYNNSNAAMPAFAFPETLGCGGGGVEQLI

>LOC_Os01g51140.1

METFGWAGGGQLMHHDDIYLPRSVGCGRPFELDDAFLGACFGAQLQCDGGVGGGDGGGCL

QGTSGFGAVAGDPLGLLCSGDVFASVAEGAGGAHDDGLLDAALAFSRNQLGGAACDGSDG

GAVSNGAMLSSYSGTTGGNISSGESNNYSGGGGGYDAEVVSPTSTMSAATQSLHPKRKLY

DDHHHPAGIAAAAAAPPLAPCPRPTTGAVAAKRRASTSATSITFGHQPHHHHAGATTAGY

EPDMEAMAQVKEMIYRAAAMRPVHLGTEAAADKPRRKNVRISSDPQTVAARLRRERVSDR

LRVLQKLVPGGNKMDTASMLDEAASYLKFLKSQVQKLETLGTTTTTSKLPQQYYSGNINS

SNNHHGFLGFAANNNTISAGYANSNAGNATKLL

>LOC_Os05g46370.1

MYMDAFGWSAPAAPCQPSCGPGGDDDDDVLLAAVLGASFELHSLVDGGGNGAAGAVRSDD

AYGLDVDLPSHQMSLLRCQDGLSALHGDASPTAAAAAFLDSVDVLPVPAIAGATHDDGGL

LDRFAFPNVAETTTVQAAASNTAFSGYSSNTTGGGNISSGESNTYTEVASTPCAVSTTTT

TTALPPSKRKLPEKYPVVGTSPTTKTTTTSETAAERRSTKRGAGGSSSITFGGGCHGAGA

AAALLGYGRGYEPDTEAIAQVKEMIYRAAAMRPVTLGGPASASDPSSRPPPPPQRPRRKN

VRISSDPQTVAARLRRERVSERLRVLQRLVPGGSKMDTATMLDEAASYLKFLKSQLEALE

TLGNGNGNGNLLHHGYYTGSRNATATAATGSSNSTVLAFGRDGLAGFVKSNRNLQL

>LOC_Os09g28210.1

MDFDLFNSYPESQLDLMSTMLQLEQLTALSDQSLFMAAPTSPPVSPMGTPSPQFSPPPQM

SVTTTTAGGGYQDQYNSMPATYGAGAGVHQLDFAMSSPGSDSGAPQGSSSSSSSEAMREM

IFHIAALQPVEIDPEAVRPPKRRNVRISKDPQSVAARLRRERISERIRILQRLVPGGTKM

DTASMLDEAIHYVKFLKSQVQSLERAAAATGAAAHRAAAFGAAYPAALPMQHHAPW

>LOC_Os08g36740.1

MEFDMLNSNPEAQLELMNTMLQLEQLSAFPDHHGMVVPCSPTSPCMGAQGGHHHFSSVNH

QPAHGVVSSGGANTGDGYRDQYYTQLLPAAAYSNAAGGGRGSEYHTTTTTRPASGGGGDG

GVGPAAMREMIFHIAALQPVNIDPETVRPPKRRNVRISTDPQSVAARMRRERISERIRIL

QRLVPGGTKMDTASMLDEAIHYVKFLKTQVQSLERAAAANGHRPPPPTATSAAAATVAYP

GLNGQW

>LOC_Os06g10820.1

MEFDMAMDMMSQEQLMHIISQLDSALASSPSPSTSPSASPPRQSPAAHVPVPPGLLNTTM

VSTSRAQAAPSAPLHPVAATAAVQSSSRGIMYTTTRQGVIDAAEEEEAAAPRPRRRNARV

SSEPQSVAARLRRERVSQRMRALQRLVPGGARLDTASMLEEAIRYVKFLKGHVQSLERAA

AALHMHGGHAAAAGFAGDAGDAVYSCPSYYA

>LOC_Os01g61480.1

MHDPRGFPIHPQPYHLHPTAGGLGEGRMRGGGRRRPGAKLSTDPQSVAARERRHRISDRF

RVLRSLVPGGSKMDTVSMLEQAIHYVKFLKAQVTLHQAALVQHEEGCQHADVAAAFSAAD

ADLALELNHRHGGAGDDDAGMTTLEMAPMQEAVGYGDGPAHQMMQQALDPAGQLMMGGAH

QLPPLPCCVFVQETDPSCYSVCNVHGEESGAQGSY

>LOC_Os08g01700.1

MDNMTHNSSSSSSWDLDMSLGSHHHPLLFDQPPPPPPPPPPPPLPFHLHHHPLDPSPSSS

LFPPPPHHHHHAHHLHHPLDLDQRRGHHDYGGGDQGGDEELRLQQEAAAGGGGGGQDGGG

GGDQDADEELGAMKEMMYRIAAMQPVDIDPATIKKPRRRNVRISDDPQSVAARHRRERIS

ERIRILQRLVPGGTKMDTASMLDEAIRYIKFLKRQVQELQHQPGPPPPPYPAGAAPAAGP

STSAVGPPGRPFLPLGGGGPMIDWVGLTRPVDIHGPTSSSSSSSMGGALGFGFGCGGGGQ

SSHGMH

>LOC_Os01g02110.1

MMAAQASSKRGMLLPREAVLYDDEPSMPLEILGYHGNGVGGGGCVDADYYYSWSGSSSSS

SSSVLSFDQAAVGGSGGGCARQLAFHPGGDDDDCAMWMDAAAGAMVENTSVVAGGGNNYC

HRLQFHGGAAGFGLASPGSSVVDNGLEIHESNVSKPPPPAAKKRACPSGEARAAGKKQCR

KGSKPNKAASASSPSPSPSPSPSPNKEQPQSAAAKVRRERISERLKVLQDLVPNGTKVDL

VTMLEKAINYVKFLQLQVKVLATDEFWPAQGGKAPELSQVKDALDAILSSQHPNK

>LOC_Os02g48060.1

MRMALVRERAMVYGGGCDAEAFGGGFESSQMGYGHDALLDIDAAALFGGYEAAASAGCAL

VQDGAAGWAGAGASSSVLAFDRAAQAEEAECDAWIEAMDQSYGAGGEAAPYRSTTAVAFD

AATGCFSLTERATGGGGGAGGRQFGLLFPSTSGGGVSPERAAPAPAPRGSQKRAHAESSQ

AMSPSKKQCGAGRKAGKAKSAPTTPTKDPQSLAAKNRRERISERLRILQELVPNGTKVDL

VTMLEKAISYVKFLQLQVKVLATDEFWPAQGGKAPEISQVKEALDAILSSSSPLMGQLMN

>LOC_Os06g30090.1

MAMVAGDEAMSVPWHDVGVVVDPEAAGTAPFDAGAGYVPSYGQCQYYYYYDDHHHHPCST

ELIHAGDAGSAVAVAYDGVDGWVHAAAAATSPSSSSALTFDGHGAEEHSAVSWMDMDMDA

HGAAPPLIGYGPTAATSSPSSCFSSGGSGDSGMVMVTTTTPRSAAASGSQRRARPPPSPL

QGSELHEYSKKQRANNKETQSSAAKSRRERISERLRALQELVPSGGKVDMVTMLDRAISY

VKFMQMQLRVLETDAFWPASDGATPDISRVKDALDAIILSSSSPSQKASPPRSG

>LOC_Os07g39940.1

MAQFLGAHGDHCFTYEQMDESMEAMAAMFLPGLDTDSNSSSGCLNYDVPPQCWPQHGHSS

SVTSFPDPAHSYGSFEFPVMDPFPIADLDAHCAIPYLTEDLISPPHGNHPSARVEEATKV

VTPVATKRKSSAAMTASKKSKKAGKKDPIGSDEGGNTYIDTQSSSSCTSEEGNLEGNAKP

SSKKMGTRANRGAATDPQSLYARKRRERINERLRILQNLVPNGTKVDISTMLEEAVQYVK

FLQLQIKLLSSDDTWMYAPIAYNGVNISNIDLNISSLQK

>LOC_Os03g10770.1

MEDSEAMAQLLGVQYFGNDQEQQQPAAAAPPAMYWPAHDAADQYYGSAPYCYMQQQQHYG

CYDGGAMVAGGDFFVPEEQLVADPSFMVDLNLEFEDQHGGDAGGAGSSAAAAAAATKMTP

ACKRKVEDHKDESCTDNVARKKARSTAATVVQKKGNKNAQSKKAQKGACSRSSNQKESNG

GGDGGNVQSSSTNYLSDDDSLSLEMTSCSNVSSASKKSSLSSPATGHGGAKARAGRGAAT

DPQSLYARKRRERINERLKILQNLIPNGTKVDISTMLEEAVHYVKFLQLQIKLLSSDDMW

MFAPIAYNGVNVGLDLKISPPQQQ

>LOC_Os12g39850.1

MEGGGLIADMSWTVFDLPSHSDESEMMAQLFSAFPIHGEEEGHEQLPWFDQSSNPCYYSC

NASSTAYSNSNASSIPAPSEYEGYCFSDSNEALGVSSSIAPHDLSMVQVQGATEFLNVIP

NHSLDSFGNGELGHEDLDSVSGTNKRKQSAEGEFDGQTRGSKCARKAEPKRAKKAKQTVE

KDASVAIPNGSCSISDNDSSSSQEVADAGATSKGKSRAGRGAATDPQSLYARKRRERINE

RLKTLQNLVPNGTKVDISTMLEEAVHYVKFLQLQIKLLSSDEMWMYAPIAYNGMNIGLDL

NIDT

>LOC_Os03g42100.1

MESGGVIAEAGWSSLDMSSQAEESEMMAQLLGTCFPSNGEDDHHQELPWSVDTPSAYYLH

CNGGSSSAYSSTTSSNSASGSFTLIAPRSEYEGYYVSDSNEAALGISIQEQGAAQFMDAI

LNRNGDPGFDDLADSSVNLLDSIGASNKRKIQEQGRLDDQTKSRKSAKKAGSKRGKKAAQ

CEGEDGSIAVTNRQSLSCCTSENDSIGSQESPVAAKSNGKAQSGHRSATDPQSLYARKRR

ERINERLKILQNLVPNGTKVDISTMLEEAMHYVKFLQLQIKLLSSDEMWMYAPIAYNGMN

IGIDLNLSQH

>LOC_Os11g41640.1

MDARCANIWSSADARSEESEMIDQLKSMFWSSTDAEINFYSPDSSVNSCVTTSTMPSSLF

LPLMDDEGFGTVQLMHQVITGNKRMFPMDEHFEQQQKKPKKKTRTSRSVSSSSTITDYET

SSELVNPSCSSGSSVGEDSIAATDGSVVLKQSDNSRGHKQCSKDTQSLYAKRRRERINER

LRILQQLVPNGTKVDISTMLEEAVQYVKFLQLQIKLLSSDDTWMFAPLAYNGMNMDLGHT

LAENQE

>LOC_Os12g32400.1

MECSSFEAICNESEMIAHLQSLFWSSSDADPCFGSSSFSLISSEGYDTMTTEFVNSSTNV

CFDYQDDSFVSAEETTIGNKRKVQMDTENELMTNRSKEVRTKMSVSKACKHSVSAESSQS

YYAKNRRQRINERLRILQELIPNGTKVDISTMLEEAIQYVKFLHLQIKLLSSDEMWMYAP

LAFDSGNNRLYQNSLSQE

>LOC_Os03g55550.1

MAVDWIWERRRREEEYNHQMSQDELQQPGQVQWTPAPEEKSEIAVQFFTAPYPCQNGQLD

HGEHHALGGIGACSSVHWQPDRGTCYWPPPLSGDGGGGSGSGSSGTGEGSYIGERCYYVG

EPDVPIGLNLLVGDNDGAGVVLRDAAPQAKRRTQAGHGGDLGRQKKKARVSDKRNQESMQ

SGSCSDNESNCSQVNRRKVDRVAGGGNGKVPARRRSATIAQSLYARRRRERINGRLRILQ

KLVPNGTKVDISTMLEEAVHYVKFLQLQIKVEVQIVCHDQMLSSDELWMYAPIVYNGMDL

GIDLNISPPR

>LOC_Os12g40590.1

MMSFPYSSGDLGEATTAAAAAVDMITLDQMFRDYDASTGDDLFELVWESCGGGEIDSGAG

EVQPAGVPCCRRLLPGSSPEPTSEDEMAAWLSTIVTGSGGGGGDDVAAGGDHQDPAVKKP

DGEPLTEKMDKKLPTRTEERRRVKHKARRNPGYAETHGLTEKRRRSRINEKFKMLQRLVP

GCDKCSQSSTLDRTIHYMKSLQQQLQAMYPTMVRPAAVYPVVQPPPAFAAGGPPAASQGG

LHRHRRLMVVVSVHHRCRCFRLGRQ

>LOC_Os12g40710.1

MAAWLYPIVGGHQVAAGGWLSPEAGDDRRAAPPAPEKKQMDNMPAVASPTMNKDEASDDS

GERKKKKKKASSAAGKASRHRHAAGAHNLTEKRRRFKITERFRTLQRLVPGCDNKSNQAS

TLDQTIQYMKSLQHQLEATSAVGSPAAAVLYPAAVHPQSYMHPPAPPPPVAGAAAPVTMA

PMPAGMVLAAAPPGAAPPPGPPATMMPFGAMLPYPPYPEVLLPPPPATLYGRPPAAAPSV

AARRHGSTSGGGRISKSSSSSLRKEL

>LOC_Os12g40630.1

MEMTSYNDVDAGDMFAAADYYSAGAGGDLFDMVWPGRRRREEDNTSGCLPLSPPPPPELA

VDDQLLAGGDGGGGKPVAVAVAEDDDSGERWTEDQVPTDEGICVMQKRRCKINENLKTLQ

QLVPVCDKSNNQASTLDKTIRYMKSLQQHAQAMSVGCSMKTAEAAGVTCPFLPPPPSSAL

LVIVEEGPTRPWDATARSTSAAATSFDHHHHGALIEKLLQVVQPYMLSLKTSVRENNAMV

MQKPNFYHS

>LOC_Os03g27390.2

MGEKVNPWCHWSNPPWTESSANNLHPPDVSLDNTNSVALPTYLNSDGYIYSGVAASMPSI

AASVTDRPVSFSSRFVTTLVPSVGLSTAETLRKRPLVFFHNVNNTFTVGPLLSKGTLDTV

PELQGSNETNVTDVGAQNTECMHENTEEIDALLCSDSDEGCLKVQELNNRVRKYPMQNDT

MSVESVASAGASQPAKKRRLSSGTDRSVVDTASSARPDHSVDQKHLSHDDDAQSCCIGEV

ESDHQFALREGEEAEGDDGPDDRKRRRERIQETVAALRKIVPGGIAKDATAVLDEAICYL

KYLKLKVKTLGAVSL

>LOC_Os02g21090.1

MQGDHGYYGGRDSPPQGYGYGGGYGYGGGYGYDAGGYYSGGGGGYPSAGAAAAAYEDPMV

GRRTHDFPAPMNELEFQPSATCPKNYVIFDQTCTKSRVMFHPSLAHKFGGGSSAYDNNVY

GGGGAHDAAKGAYRDSVGYDDDDSCSVRQKEDTDEIDALLSSEDGDEDDVVSTGRTPGYR

DGSSPDSTCSSSYGGGQARPGRKKERMKKMVRTLKGIIPGGNQMDTPAVLDEAVRYLKSL

KVEVKKLGVRGSDN

>LOC_Os03g39432.1

MQGDPGYGYGGYGYGYGYGYGGAGYDMAGYGGGGGGYYTSDPYNAAPAAYEDPLAVAGRR

QHDFPAPLTGVEFQPSDTCPKNYVIFDQTYDRSRVMFHPSLANNLGNSGGGYDHHHHCGY

GGFEQDYASKSAYYGVEDDGGGGCSIRQKEDTDEIDALMSTEDGEEEDDVLSTGRTPGCR

AGGSPDSTCSSGASRSDCGGGRKPEAGGGERKKERMKKMMRTLKGIIPGGDRMDTPAVLD

EAVRYLKSLKVEVKKLGVRGSSS

>LOC_Os04g51070.1

MIVGAGYFEDSHDQSLMAGSLIHDSNQAPASSENTSIDLQKFKVHPYSTEALSNTANLAE

AARAINHLQHQLEIDLEQEVPPVETANWDPAICTIPDHIINHQFSEDPQNILVEQQIQQY

DSALYPNGVYTPAPDLLNLMQCTMAPAFPATTSVFGDTTLNGTNYLDLNGELTGVAAVPD

SGSGLMFASDSALQLGYHGTQSHLIKDICHSLPQNYGLFPSEDERDVIIGVGSGDLFQEI

DDRQFDSVLECRRGKGEFGKGKGKANFATERERREQLNVKFRTLRMLFPNPTKNDRASIV

GDAIEYIDELNRTVKELKILVEQKRHGNNRRKVLKLDQEAAADGESSSMRPVRDDQDNQL

HGAIRSSWVQRRSKECHVDVRIVDDEVNIKLTEKKKANSLLHAAKVLDEFQLELIHVVGG

IIGDHHIFMFNTKVSEGSAVYACAVAKKLLQAVDVQHQALDIFN

>LOC_Os01g18870.1

MYHPQCELLMPLESLEMDVGQSHLAAAVAAAMPGELNFHLLHSLDAAAAAASSTAASASS

QPTVDYFFGGADQQPPPPAAMQYDQLAAPHHHQTVAMLRDYYGGHYPPAAAAAAATEAYF

RGGPRTAGSSSLVFGPADDESAFMVGPFESSPTPRSGGGRKRSRATAGFHGGGPANGVEK

KEKQRRLRLTEKYNALMLLIPNRTKEDRATVISDAIEYIQELGRTVEELTLLVEKKRRRR

EMQGDVVDAATSSVVAGMDQAAESSEGEVMAAAAMGAVAPPPRQAPIRSTYIQRRSKETF

VDVRIVEDDVNIKLTKRRRDGCLAAASRALDDLRLDLVHLSGGKIGDCHIYMFNTKIHSG

SPVFASAVASRLIEVVDEY

>LOC_Os09g31300.1

MYGAPVSKDLSLQPAGVRTPPQMSSPGLLRYRSAPSTLLGEVCGDFVLPGGGGGGGQLQL

QLQQQRPGSPDHAADTVLARFLAGHGGHDNKPPRPAAHFAPPEDSMASHQQQLMYQSHQQ

QQQMASAMEGLYRTVSSGGTESTAAAAGNSLLRQSSSPAGFLNHLTMDNGYGNMLRAGMG

GGGGGGDPRLKGQLSFSSRQGSVMSQISEMGSEDEELAGGGGSPEAGSNGGGAARGGYGG

GYAMGSSAWEEPSPPATSLLPDSSLPSKRPRDDLPRQLSLPAASKNSSKPPSSASAAASP

EMAAIEKFLQFQDAVPCKIRAKRGCATHPRSIAERVRRTRISERIRKLQELVPNMEKQTN

TADMLDLAVDYIKELQKQVKVLNDSRSSCTCSASKQKHFAG

>LOC_Os04g35010.1

MSQEGADLSQDVDESPYHTAVVTTNNLVRSIKAEKSNSSSSSGKPVETDIGLKVASPTMF

GFNTKIEGTGKNMAVKREEGEGGGRPGVSSGVSTRDTNGKGKNAMDMEHALHIWTERERR

KKMKNMFSTLHGLLPKIPGKTDKASIVGEAIGYIKTLEDVVQKLETIKTERVRAHQWAAA

AAAAVAANGGGEGSSHSHSQPPRHATAVTVAVAEPAPVAAAVNAQAPQKKAAAAAAPTLQ

TWSAPNITLTMAGVDAFINMCLPRQRASFTTVAFVLEKHQIDVVTSTISADHDKSLFSVH

VRLNEASLQSTEGLTPEAKYKLAVSELMVRLAE

>LOC_Os04g35000.1

MEMLKVEREHAIALATAATATAAASADTALQAPPPSEEENEEHDSVVAAATREMALADMV

HAWEQQQEAAATGGSHGGHAVPPPPPAASLQTWTGPNMTASLTGDDGFITLSLPHQGGQK

NLVAGAVSVLERHHIDVVTATVSASEQGDNLISLHCHLSPGSSSSQNLTPLDKFKLAMSE

LMLWVISV

>LOC_Os02g34320.1

MAQEGTSSNAPPAAASMGSGDGDNKEGTGESGNNQLLLPAIAASADKGKGVVAGTGNVDA

KGKTTAMAPAASSTNAPNNQGGGGGGGGRSRERMHIFAERERRRKIKNMFTDLRDLVPSL

TNKADKATIVGEAISFIRSLEETVADLERRKRERNSLAARCARLGLGGSSSSSAPPPPPP

PAAADDTAAVMPPAPAVPPPDAAAVTAGPEPAPAPAPGTLMVWSGPSVVLNLCGGDQAFI

NVSVARRPGVLTMIVDVLERHSIDVVTAQIASDLSRSLFTIHTSVDRERGMFMDTATAEE

IYQLAVSEIM

>LOC_Os02g34370.1

MSQEEQRHGRQLPLATSGVITFNGVPLSPLTAGAESDGDDDDVVIVDACNNGKRKVGDGE

EEGRGSQGGDDDDDVVAVHGGGGGGGNRACMFAVRERERRRRMNDMFAGIRRLVPNLPEK

GEEVRMEAQKQELQRERDRLAMEVAAAAGGAGASSSSSSAAAAAVTVAAYGPAGAVGSSS

SSAAAAARANTVVPVPQRAARGATAPTPRPVAPPQAGTWPLPAPAAMPPPPPPPTGAAAV

APPPSLKTWSWGDNVVVSVLGNIGNMTVRAPLRRTGVLAMASAALKRYNITAVTSLSGTD

ASRTQNMFMFYTIIDMPDRQPQFVHPKVFEAMYRAAAAEIAAWINCY

>LOC_Os03g53020.1

MQMESYYGAFHADEAAFFFPHHVPASPELPFGLIASPEPEPEPEQAAAEARQSAFQEYGG

AVHAGAPAAAGAVTTGGTNIHRRVMDVLGRMGGGGGGGEKGEGEEMEEEEEVPQRRRRGQ

GADVESSRGFRHMMRERQRREKLSQSYADLYAMVSSRSKGDKNSIVQSAAIYIHELKVAR

DQLQRRNEELKAQIMGHDEQQPCVTVQFEVDEPSSSIDSMIAALRRLKGMSVKARGIRSS

MSGNRLWTEMNVETTIAACEVEKAVEEALKEVERNQPDSDAPFPGSKGWTQTSHVQNVF

>LOC_Os01g64560.1

MAGAAPLRDSLRRLCTDVGWSYAVFWRATRAADSQRLKLVWGDGHYERAAGAPSISGFEA

MDLLLKEKAAALRSGTGRGGGGGEGHAADGAAGHSHDRVDALVHKAMAQQVHVVGEGVIG

QAALTGLHRWIVHDIVDECEEEDEVLLEMKGQFCAGIQTIAVIPVLPRGVIQLGSTKMVM

EEAAFIDHVRSLFQQLGSSTAVVPCGSFVQDSIMRTPFHKSLGVPTSSHSEDLAGGGNTY

NDDMINHQFRHQKSPASTIQSFNPVQQFYAGPTFCRPVTIASRCDLFQPDHGSTFTLNSQ

SEDNRSTALLKNSVSHSKTSNDAFSHAFNPLNEPNVSISGRRECVSIEQHGSCRNGEMEI

TIGRTASSSCTGKTNIINKVDDLLSQDCLVGCQASNATSVNRKFQTMSIVDNTKLQDGSY

AIPHAALVDSTQYSDCFQSLLGTIQGSSSSNSNAIHVDTSHNAVHGKSNFCPLGDRNAAN

SSDLAELLASPIPLELTGGNDLFDVLQLQQKPNGSNNSEVNNRQSMPYGSEQAVKSLIGC

VDDDFTGLITEADPDQLLDAIVSKIITGHKQNVDTSASCSTTVAGFDRPLHSDCHLYTTG

PSSGPIFCNFASVAPVAIKTEGPAAGSRQSSSSIDKSAGCSQTQESYKSQIRLWVENNHS

VGSDSLSTGQASDSLSTGQCKRSDEIGKSNRKRSRPGESARPRPKDRQMIQDRIKELREI

VPNSAKCSIDTLLEKTIKHMLFLQNVAKHADKLKGSGEPKIVSHEEGLLLKDNFEGGATW

AFEVGTRSMTCPIIVEDLNPPRQMLVEMLCKERGIFLEIADQIRGLGLTILKGVMEVRKD

KIWARFAVEANKDVTRMEIFLSLVHLLEPSTGSSILSAGVENTSLPRDSFFPSSIPASGF

SNCL

>LOC_Os12g06330.1

MAVGDALRRLCEEARWSYAVFWKAIGAADPVHLVWEDGFCGHASCSAGSEASEAGCESGG

AVCTLVRKIMASQVHVVGEGTIGRAAFTGNHQWIVHETANDHGLRSEVVAAEMNNQFRAG

IKTIAIIPVLPRGVLQLGSTSVILENISSVQQYKKLCCQLNNRSSMVASASAKNDLSQKV

QSRSLHGLPSIHPYEQCYGHDARALSSSTSANTGRNTSLLKVAQRNDQAIREQVLYAPDM

RFRQQLPYSDRRVDINTHSSAMSSGFISSISASVEKYPLLTNNIGQVEHGNMEESSGPRN

VLLKSLSCRNPVVHENTNTSLFHGGDEVPAFLNSHGSFDFLQAGPRVVEANLYNNGTSSQ

VLDQRCSSTSGMAGYKPSVSYKFPHSAQFIVKMENPRRQSFQDPAAPSSGSDVQVSSGLK

TTTRQFNPEHMCQNKKTNEVNDSSAAVSTQDVKNMDRHKILDISNERTSSFVMDPSTEND

LFDIFGTDFHQLHRSLDGDLSWNTAKPQSSDRDAPESSIYLDSSPAFGAQEDEFSYSGIF

SLTDTDQLLDAVISNVNPGGKQISGDSASCKTSLTDIPSTSYCGSKETKQCKSSGAPPLL

IKNELAVSNFVKQPCFLEKAEDGCLSQNNGVQKSQIRLWIESGQNMKCESVSASNSKGLD

TANKANRKRSRPGESPKPRPKDRQLIQDRIKELRELVPNGAKCSIDALLEKTIKHMVFLQ

SVTKHADNLKDSNESKIHGGGENGPLLKDYFEGGATWAFDVGSQSMTCPIIVEDLDRPRQ

MLVEMLCEDRGIFLEIADFIKGLGLTILRGVMEARKNKIWARFTVEANRDVTRMEIFLSL

MRLLEPSCDGGGGGVGDNPNNVKIPPGIVQHPVIPATGHLR

>LOC_Os11g06010.1

MAVGDALRRLCEEVGWSYAVFWKAIGAADPVHLVWEDGYCGHASCPAGSDPSEALPTDVG

CAAAADTMTMCSLVNKVMASQVHVVGEGTVGRAAFTGNHQWIIHGTANDHGIPSEVAAEM

SYQFRVGIQTIAIIPVLPRGVLQLGSTGVVLENKSFMTHAKKLCSQLNNRSSMAVSSSVK

NSSSQQGRSRPLHGASNVQSTENRSKLFSQFPVTCEQYNHPDTMAVSGSTSLNACMNGSL

LKIAQLNGQAVREHIVYSKPDVRFIQQVYRDGQLGSNAQSIAMSSDLISSSLRSVQKQPL

LMNNISQLEYGDGAETSADLRKNVLLKPPVCLDPFIHDRNINISHGITEVSNVINDHGNF

DFLSGGARVVRANLCTSATSQVLDRRSHSVSGMLLHREPIVSCEVPQSSEFSTKMGSLER

GSFQISSAPSSESDVQISNGLNTSISRENQLSVSNHICQDQKINGVNDLSATLSTERMNN

MDGCKPPGLSLERTSPLFMEQSVENDLFDILGPQFHHLCHNAGADLVPWTDAKPESSDRD

VPESSIHADSAPLFSSRDNELYSGIFSLTDTDQLLDAVISNVNPAGKQSSDDSASCKTSL

TDIPATSYLCSKEMKQCGSSGVPSVLIKNESAQFIKQPCLAENAEDGCLSQNNGMHKSQI

RLWIESGQSMKCESASASNSKGLDTPSKANRKRSRPGESPKPRPKDRQLIQDRIKELREM

VPNGAKCSIDALLEKTVKHMLFLQSVTKHADKLKDSTESKILGNENGPVWKDYFEGGATW

AFDVGSQSMTCPIIVEDLDRPRQMLVEMICEDRGIFLEIADFIKGLGLTILRGAMEARKS

KIWARFTVEANRDVTRMEIFLSLVRLLEPNCDSSGAAENANNVNMPLGLVHQPVIPATGR

IQ

>LOC_Os03g56950.2

MDGNARSAANQTKQIVTDNELVELLWHNGGVVAQPQAAQARVVSSSGRGQSASVLTGDDT

ETAAWFPDTLDDALEKDLYTQLWRSVTGDAFPAAAAAGPSSHHAPPPDLPPPAARPPMRS

GIGSSWTGDICSAFCGSNHIPETAAQRCRDAGAALPPERPRRSSTHDGAGTSSSGGSGSN

FGASGLPSESASAHKRKGREDSDSRSEDAECEATEETKSSSRRYGSKRRTRAAEVHNLSE

RRRRDRINEKMRALQELIPHCNKTDKASILDEAIEYLKSLQMQVQIMWMTTGMAPMMFPG

AHQFMPPMAVGMNSACMPAAQGLSHMSRLPYMNHSMPNHIPLNSSPAMNPMNVANQMQNI

QLREASNPFLHPDGWQTVPPQVSGPYASGPQVAQQNQIPKASASTVLPNSGAEQPPTSDG

I

>LOC_Os03g07540.1

MSSRRGGGGGGGRITDEEINELISKLQALLPESSRSRGASRSSASKLLKETCSYIKSLHR

EVDDLSDRLSELMSTMDNNSPQAEIIRSLLR

>LOC_Os04g54900.1

MSSSRRSRSRRAGSSVPSSSSSSRTSISEDQIAELLSKLQALLPESQARNGAHRGSAARV

LQETCSYIRSLHQEVDNLSETLAQLLASPDVTSDQAAVIRSLLM

>LOC_Os06g50900.1

MELDEQAFLEELFSLRRDAWEYNAMGDFFSPACAAMDGFQERHQSTTTVSVLPTFTASYE

QPPPAPAAGFDCLSEVYGNAAAAFGPNAGGGGGEYGGGGDMGFLDVVEPKASMVVDGGGL

GVCKVEPGLQAEGGFSAAAAAPASKKKRVEGMPSKNLMAERRRRKRLNDRLSMLRSVVPK

ISKVYRVLSESFCVAIGIASAREMRLDDVCVCE

>LOC_Os04g31290.1

MEHHHLLLQLSPPPPPPPLPAAHLMMSPSFFDAGVFADVGGDWMEDLMHLGELFGVGVGG

DDDDNGGVDGGVGGGDDRMQEWQNNCEGAGSPDHQPSCGDGDGDGDGDVSPRDGELGDGD

GDNSATRKRRDRSKTIVSERKRRVRMKEKLYELRALVPNITKMDKASIIADAVVYVKDLQ

AHARKLKEEVAALEEARPIRPPPPSAAAQRPQRQPRRVAAAAAQLARAADAAAVTTAAAA

PHGARVAHVGAAQVGEGRFFVTVECEPAAAAARGGGGGVAAPVCAAVESLSCFTVESSTV

GCSPDRVVATLTLKVSEAEEDVSAISECTVKLWVMAALLKEGFRPQPTVQIS

>LOC_Os02g08220.1

MRGPSTTTMATFKQSFLKNLLSSLKSSSKNKAAMSTLSERKRAIKSSADIAMATARTGIA

GAARWPHAILASSSSSSSSSSSSSSSSSMPRTTFPCKMMQGKVRRRCKSIVRRRTPLMSS

SSEVARRLVKKRDKVLRRMIPGGELIADEISLLHEAMDYVVHLHAQVDVLRRVSRAAVAR

RSSASSSSSGGLAQLKERTVQISGETENPC

>LOC_Os06g44320.1

MGGPGASSTMSFEQAFLKNLLLSLQDCSTTKPLDAMSLHERKRAVKSSADFAMATARGGG

ARWPKAIVLQQQPASTTARARRCGRIVRRCCGRKTRSGAGGGGEMARRLQVRRRAMALRK

VIPGGGDAMDEAALLREAMDYVVHLRAQVDVLRRVSEAVQLQRRYTSSTSLRDYSWSKCA

LKGEEDSSMNMKR

>LOC_Os05g06520.1

MPPPASNHGAACKNSGSNKERRRMRRCVEVRRKMEALRRLVPGGGGGEDSGEELLFRAAD

YIARLQVQVKVMQLMVDVLEQTKD

>LOC_Os11g02054.1

MSGKDQKKATTALEEKLELLRDVTKSSAANETSILVDASKYIKELKDKVSQEPEQLGSTS

SSMPMPRVSVSSVELEKKIGFRINVSMEKSQPELLTSVLEAFEELGLDVLDADVSCADDT

AFRLEALGSSQSEAAETSVDEQMVRHAVLQAIKKCIDGSSI

>LOC_Os12g02020.1

MSGKDQKKAAALEEKLELLRSVTKSSAANETSILVDASKYIKELKDKVSQEPEQLGSTSS

SMPMPRVSVSSVELEKKRGFRINVSMEKSQPELLTSVLEAFEELGLDVLDADVSCADDTA

FRFEAFGSSQSEAAERSVDEQMVRHAVLQAIKKCMDGSSI

>LOC_Os05g27090.1

MVSREQKRASLHEKLQILRTLTHSHAVNKMSIISDASTYIKDLKQKIAALNKELGCAKNM

NICEEPSPVVRVQVLDKGFLINVFMDKSSPGLLSSILQAFDELGLTVIEARASCSNSFRL

EAVGGEHEEADGGIDANAVELAVMQAIKSTPGK

>LOC_Os07g47960.1

MMSRERKKAAALHEKLQILRSITHSHALSNTSIITDASEYIKELKQKVVRLNKEIACAEA

AALRQNSIPTVTVETLGHGFLINVFSDKSCPGLLVSILEAFDELGLNVLEATASCDDTFR

LEAVGGENQVDEHVIKQTVLQAISNSNCSESSGDQEG

>LOC_Os07g36460.1

MPRRARARGGGGGGGEEVKVEDDFIDSVLNFGGGGGGEEDGDDGEEEQQQQQAAAAAMGK

EFKSKNLEAERRRRGRLNGNIFALRAVVPKITKMSKEATLSDAIEHIKNLQNEVLELQRQ

LGDSPGEAWEKQCSASCSESFVPTENAHYQGQVELISLGSCKYNLKIFWTKRAGLFTKVL

EALCSYKVQVLSLNTISFYGYAESFFTIEVKGEQDVVMVELRSLLSSIVEVPSI

>LOC_Os01g39580.1

MSRVPTSWGFVYSRVNKVSEEPNVVNRITTAFWELQLPACSDEPISSGTPSSPSSPPTKE

TGDANTVVIDDLFLAHSDAILPAGGDQKEEHQLGDDLGQQQAATAMEIDDDVIYSLIRNW

DNDSSSSWIELLDHAIVSPASCFVPWKRTELDKEAVAGGEAAQRLLKKVVGGGGAWMNRA

AGSCSIKNHVMSERRRREKLNEMFLILKSLVPSIDKVDKASILSETIAYLKELERRVQEL

ESGKKVSRPAKRKPCSETIIGGGGGGGGAGAVKEHHHWVLSESQEGTPSDVRVIVMDKDE

LHLEVQCRWKELMMTRVFDAIKSLRLDVVSVQASAPDGLLGLKIRAKYASSAAVVPAMIS

ETLRTAVAGY

>LOC_Os03g21970.1

MMSSRERKKAAALQEKLQILRSITHSHALSNTSILMDASKYIKELKQKVVRLNQEIACAQ

DALRQNRVTVETLRHGFLVNVFSGKSCPGLLVSILEAFDELGLSVLEATASCTDTFRLEA

IGSENLMEKVDEHVVKQAVLRAIRSCSGSGGDHHDDDDDDDDE

>LOC_Os09g34330.1

MDELLSPCSSFSPPSPSSMFSTGAAAAAAHAVLEFTSCEVPDEWLMGDVVMAKNEEDVGG

GELWPVFAGGSLSPDSELSELPRSFEAAAAQRPAKRRGRKPGPRPDGPTVSHVEAERQRR

EKLNRRFCDLRAAVPTVSRMDKASLLADAAAYIAELRARVARLESDARQAAAARFEPSSC

GGGGNASYHGGGGGGGAAPGLDEAVEVRKMGRDAAAVRVTTTGARHAPARLMGALRSLEL

PVQHACVMRVHGATTVQEVLVDVPAALQDGDALRAALLQRLQDS
