# Supplementary material for: Systematic identification and expression analysis of bHLH gene family reveal their relevance to abiotic stress response and anthocyanin biosynthesis in sweetpotato
Source: BMC Plant Biol. 2024 Mar 1;24:156. doi: 10.1186/s12870-024-04788-0 (PMC10905920; doi:10.1186/s12870-024-04788-0)
Supplement: Supplementary file 5 — Supplementary Material 5 [file 12870_2024_4788_MOESM5_ESM.docx]

| Primer code | Primer sequences (5' →3') | Application |
| --- | --- | --- |
| IbbHLH5-Clone-F | TGTCAACTTTTTCTTCTCAGCTC |  |
| IbbHLH5-Clone-R | GTTGAACGATTATGTTTTTGGAA |  |
| IbbHLH5-BD-F | TGGCCATGGAGGCCGAATTCATGATGAATCTTTCCCAGGC | |
| IbbHLH5-BD-R | CGCTGCAGGTCGACGGATCCGGATGTTTGTGTGGCATGTT | |
| IbbHLH5-AD-F | CATGGAGGCCAGTGAATTCATGATGAATCTTTCCCAGGC | |
| IbbHLH5-AD-R | GCTCGAGCTCGATGGATCCGGATGTTTGTGTGGCATGTT | |
| IbbHLH106-Clone-F | CCAATCATCTTCTCCCCTACT |  |
| IbbHLH106-Clone-R | TAAAAAATAGAAAAAGAAAATCAGG |  |
| IbbHLH106-BD-F | TGGCCATGGAGGCCGAATTCATGGAGAATTCATTCAGCGG | |
| IbbHLH106-BD-R | CGCTGCAGGTCGACGGATCCATGATAATGTGCAGGCAAGAA | |
| IbbHLH106-AD-F | CATGGAGGCCAGTGAATTCATGGAGAATTCATTCAGCGG | |
| IbbHLH106-AD-R | GCTCGAGCTCGATGGATCCATGATAATGTGCAGGCAAGAA | |
| IbbHLH123-Clone-F | CCACCCCACTCCTCTGTCT |  |
| IbbHLH123-Clone-R | TAAGTTCCAAGTTCTACTAGGCATG |  |
| IbbHLH123-BD-F | TGGCCATGGAGGCCGAATTCATGGATCCTCCGATAATCAAT | |
| IbbHLH123-BD-R | CGCTGCAGGTCGACGGATCCTGTCGCTCTCTCGAAGCTG | |
| IbbHLH123-AD-F | CATGGAGGCCAGTGAATTCATGGATCCTCCGATAATCAAT | |
| IbbHLH123-AD-R | GCTCGAGCTCGATGGATCCTGTCGCTCTCTCGAAGCTG | |
| IbbHLH212L-Clone-F | TTGAAACTGATCCAAGAGACG |  |
| IbbHLH212L-Clone-R: | TACAAGATTTTGAGAGAAGGGTT |  |
| IbbHLH212L-BD-F | TGGCCATGGAGGCCGAATTCATGGCTCATGCTACCGAA | |
| IbbHLH212L-BD-R | CGCTGCAGGTCGACGGATCC TTCTTGTCTAGCTGGCAATTG | |
| IbbHLH212L-AD-F | CATGGAGGCCAGTGAATTCATGGCTCATGCTACCGAA | |
| IbbHLH212L-AD-R | GCTCGAGCTCGATGGATCCTTCTTGTCTAGCTGGCAATTG | |
| IbbHLH215-Clone-F | CCAAAACTATTGTAGAACTGGTATG |  |
| IbbHLH215-Clone-R | TGACAAACCATCGGGAAGTA |  |
| IbbHLH215-BD-F | TGGCCATGGAGGCCGAATTCATGGAAAAGGACTTCAATTCTTG | |
| IbbHLH215-BD-R | CGCTGCAGGTCGACGGATCCGAGCGCATCAAGCCTCAG | |
| IbbHLH215-AD-F | CATGGAGGCCAGTGAATTCATGGAAAAGGACTTCAATTCTTG | |
| IbbHLH215-AD-R: | GCTCGAGCTCGATGGATCCGAGCGCATCAAGCCTCAG | |

**Additional file 16**. Specific primer sequences used for gene cloning and vector construction.
