# Supplementary material for: Systematic identification and expression analysis of bHLH gene family reveal their relevance to abiotic stress response and anthocyanin biosynthesis in sweetpotato
Source: BMC Plant Biol. 2024 Mar 1;24:156. doi: 10.1186/s12870-024-04788-0 (PMC10905920; doi:10.1186/s12870-024-04788-0)
Supplement: Supplementary file 7 — Supplementary Material 7 [file 12870_2024_4788_MOESM7_ESM.docx]

**Additional file 13**. The 141 seed sequences of bHLH (PF00010) from the Pfam database (<http://pfam.xfam.org/>).

>D2A1T6_TRICA/49-101

YRTAHATRERIRVEAFNVAFAELRKLLPTLPPDKKLSKIEILRLAICYIAYLN

>HLH15_CAEEL/33-85

YRNLHATRERIRVESFNMAFSQLRALLPTLPVEKKLSKIEILRFSIAYISFLD

>B4JNN8_DROGR/169-221

RKVFTNTRERWRQQNVSGAFAELRKLVPTHPPDKKLSKNEILRSAIKYIKLLT

>TCFL5_HUMAN/401-451

RRERHNRMERDRRRRIRICCDELNLLVPFCNAETDKATTLQWTTAFLKYIQ

>HEY1_CANLF/51-105

KRRRGIIEKRRRDRINNSLSELRRLVPSAFEKQGSAKLEKAEILQMTVDHLKMLH

>BHE40_MOUSE/53-108

YKLPHRLIEKKRRDRINECIAQLKDLLPEHLKLTTLGHLEKAVVLELTLKHVKALT

>Q9VJ16_DROME/51-115

KRTNKPLMEKRRRARINQSLAILKALILESTKTQNAKNGEGQAKHTKLEKADILELTVRH

FQRHR

>DPN_DROME/41-98

RKTNKPIMEKRRRARINHCLNELKSLILEAMKKDPARHTKLEKADILEMTVKHLQSVQ

>H9G5B5_ANOCA/35-92

RKSSKPIMEKRRRARINESLGQLKTLILDALKKDSSRHSKLEKADILEMTVKHLRNLQ

>HES2_HUMAN/14-71

RKSLKPLLEKRRRARINQSLSQLKGLILPLLGRENSNCSKLEKADVLEMTVRFLQELP

>G3IJV5_CRIGR/19-75

RKISKPLMEKKRRARINMSLEQLKSLLERHYSHQIRKRKLEKADILELSVKYMKSLQ

>ESM7_DROME/14-69

RKVMKPLLERKRRARINKCLDELKDLMAECVAQTGDAKFEKADILEVTVQHLRKLK

>ESMD_DROME/16-73

RKVTKPLLERKRRARMNLYLDELKDLIVDTMDAQGEQVSKLEKADILELTVNYLKAQQ

>ESM3_DROME/12-69

RKVMKPLLERKRRARINKCLDDLKDLMVECLQQEGEHVTRLEKADILELTVDHMRKLK

>F1RDU0_DANRE/41-97

KKVSKPLMEKKRRARINKCLNQLKSLLESACSNNIRKRKLEKADILELTVKHLRHLQ

>F1QJS9_DANRE/16-73

RRVPKPLMEKRRRDRINQSLETLRMLLLENTNNEKLKNPKVEKAEILESVVHFLRAEQ

>A3KQ56_DANRE/13-70

KRILKPVIEKKRRDRINQRLEELRTLLLDNTLDSRLQNPKLEKAEILELAVEYIRTKT

>A3KQ57_DANRE/14-71

RKLLKPQVERRRRERMNRSLENLKLLLLQGPEHNQPNQRRLEKAEILEYTVLFLQKAN

>HES6_MOUSE/27-78

KARKPLVEKKRRARINESLQELRLLLAGTEVQAKLENAEVLELTVRRVQGAL

>Q90466_DANRE/19-74

KLRKPMVEKIRRERINSSIEKLKTLLAQEFIKQQPDSRQEKADILEMTLDFLRRSQ

>Q28F58_XENTR/22-78

NKIRKPVIEKMRRDRINHSIEQLRILLERNFQTHHPHSKLEKADILEMAVSYLQQQK

>HES5_MOUSE/18-73

RLRKPVVEKMRRDRINSSIEQLKLLLEQEFARHQPNSKLEKADILEMAVSYLKHSK

>F1QI81_DANRE/20-74

RKLRKPLIEKKRRERINSSLEQLKGIMVDAYNLDQSKLEKADVLEITVQHMENLQ

>B4K528_DROMO/63-118

DPLSHRIIEKRRRDRMNSCLADLSRLIPPQYQRKGRGRIEKTEIIEMAIRHLKHLQ

>Q9VXW7_DROME/278-331

GREARNRAEKNRRDKLNGSIQELSTMVPHVAESPRRVDKTAVLRFAAHALRLKH

>CYCL_DROME/31-84

RKQNHSEIEKRRRDKMNTYINELSSMIPMCFAMQRKLDKLTVLRMAVQHLRGIR

>BMAL1_HUMAN/73-126

AREAHSQIEKRRRDKMNSFIDELASLVPTCNAMSRKLDKLTVLRMAVQHMKTLR

>H3CFF9_TETNG/56-109

SRENHSEIERRRRNKMTQYITELSDMVPTCSALARKPDKLTILRMAVSHMKSMR

>BH057_ARATH/113-164

QRMTHIAVERNRRRQMNEHLNSLRSLMPPSFLQRGDQASIVGGAIDFIKELE

>SPCH_ARATH/100-151

QKMSHVTVERNRRKQMNEHLTVLRSLMPCFYVKRGDQASIIGGVVEYISELQ

>BH099_ARATH/100-151

QRMNHIAVERNRRKQMNHFLSILKSMMPLSYSQPNDQASIIEGTISYLKKLE

>BH067_ARATH/176-227

QRINHIAVERNRRRQMNEHINSLRALLPPSYIQRGDQASIVGGAINYVKVLE

>NUC1_NEUCR/646-736

KRTSHKIAEQGRRNRINSALQEIATLLPKAPAKEGGDGDGDGHSSSGGGGGSGGADREDK

REKDKDKAGGGIPNSKASTVEMAIEYIKQLQ

>BH036_ARATH/2-54

EKMMHRETERQRRQEMASLYASLRSLLPLHFIKGKRSTSDQVNEAVNYIKYLQ

>BH125_ARATH/74-126

KKMKHRDIERQRRQEVSSLFKRLRTLLPFQYIQGKRSTSDHIVQAVNYIKDLQ

>BH055_ARATH/75-127

KRAKHKELERQRRQENTSLFKILRYLLPSQYIKGKRSSADHVLEAVNYIKDLQ

>BH101_ARATH/66-118

KKLNHNASERDRRRKLNALYSSLRALLPLSDQKRKLSIPMTVARVVKYIPEQK

>BH100_ARATH/62-114

KKLNHNASERERRKKINTMFSSLRSCLPPTNQTKKLSVSATVSQALKYIPELQ

>ORG3_ARATH/77-129

KKLNHNASERDRRRKINSLFSSLRSCLPASGQSKKLSIPATVSRSLKYIPELQ

>BIM2_ARATH/46-96

IRSKHSVTEQRRRSKINERFQILRELIPNSEQKRDTASFLLEVIDYVQYLQ

>BIM3_ARATH/34-86

CRSKHSETEQRRRSKINERFQSLMDIIPQNQNDQKRDKASFLLEVIEYIHFLQ

>BH131_ARATH/92-141

AAKKHSDAERRRRLRINSQFATLRTILPNLVKQDKASVLGETVRYFNELK

>GL3_ARATH/438-487

ETGNHAVLEKKRREKLNERFMTLRKIIPSINKIDKVSILDDTIEYLQELE

>BH104_ARATH/131-182

GGGTKACRERLRREKLNERFMDLSSVLEPGRTPKTDKPAILDDAIRILNQLR

>ILR3_ARATH/72-123

ATSSKACREKQRRDRLNDKFMELGAILEPGNPPKTDKAAILVDAVRMVTQLR

>PIF5_ARATH/257-306

AAEVHNLSERRRRDRINERMKALQELIPHCSRTDKASILDEAIDYLKSLQ

>RTG1_YEAST/12-97

SCGANFKNDRKRRDKINDRIQELLSIIPKDFFRDYYGNSGSNDTLSESTPGALGLSSKAK

GTGTKDGKPNKGQILTQAVEYISHLQ

>RTG3_YEAST/286-345

KREFHNAVERRRRELIKQKIKELGQLVPPSLLNYDDLGKQIKPNKGIILDRTVEYLQYLA

>MITF_HUMAN/312-365

KKDNHNLIERRRRFNINDRIKELGTLIPKSNDPDMRWNKGTILKASVDYIRKLQ

>HMS1_YEAST/267-342

GRVSHNIIEKKYRSNINDKIEQLRRTVPTLRVAYKKCNDLPITSRDLADLDGLEPATKLN

KASILTKSIEYICHLE

>SREBP_SCHPO/261-333

KKTAHNMIEKRYRTNLNDRICELRDAVPSLRAAAALRCGNSLDDEDLGGLTPARKLNKGT

ILAKATEYIRHLE

>SRE2_SCHPO/427-496

KRSAHNMIEKRYRSNLNDKIAELRDAVPTLRSGYNSTTADELKGTYVPLSRKLNKATILS

KATEYIKSLQ

>Q6CIW9_KLULA/180-254

QRVTHNMIEKRYRININTKIGKLQKIIPWVACEDTAFVVDNKVLSAGEDSLPLKKVKLNK

SMILEKAVDYILYLQ

>TYE7_YEAST/181-266

QKQAHNKIEKRYRININTKIARLQQIIPWVASEQTAFEVGDSVKKQDEDGAETAATTPLP

SAAATSTKLNKSMILEKAVDYILYLQ

>H0ZAF3_TAEGU/305-355

KRTAHNAIEKRYRSSINDKIVELKDLVVGTEAKLNKSAILRKAIEYIRFLQ

>Q9XX00_CAEEL/356-406

RRTAHNLIEKKYRCSINDRIQQLKVLLCGDEAKLSKSATLRRAIEHIEEVE

>B4QQY0_DROSI/284-334

KRSAHNAIERRYRTSINDKINELKNLVVGEQAKLNKSAVLRKSIDKIRDLQ

>MLX_MOUSE/130-188

RRRAHTQAEQKRRDAIKRGYDDLQTIVPTCQQQDFSIGSQKLSKAIVLQKTIDYIQFLH

>Q9VB47_DROME/72-128

RREAHTQAEQKRRDAIKKGYDSLQELVPRCQPNDSSGYKLSKALILQKSIEYIGYLN

>MLXPL_MOUSE/662-716

RRITHISAEQKRRFNIKLGFDTLHGLVSTLSAQPSLKVSKATTLQKTAEYILMLQ

>B4Q4D1_DROSI/244-298

RRAGHIHAEQKRRYNIKNGFDTLHALIPQLQQNPNAKLSKAAMLQKGADHIKQLR

>WBS14_CAEEL/804-857

KRILHLHAEQNRRSALKDGFDQLMDIIPDLYSGGVKPTNAVVLAKSADHIRRLQ

>Q9TZ70_CAEEL/48-102

KKATHLRCERQRREAINSGYSDLKDLIPQTTTSLGCKTTNAAILFRACDFMSQLK

>ESC1_SCHPO/335-386

LRTSHKLAERKRRKEIKELFDDLKDALPLDKSTKSSKWGLLTRAIQYIEQLK

>MGAP_HUMAN/2424-2475

YRRTHTANERRRRGEMRDLFEKLKITLGLLHSSKVSKSLILTRAFSEIQGLT

>Q19918_CAEEL/21-73

QTRKSVSERKRRDEINELLENLKTIVQNPSDSNEKISHETILFRVFERVSGVD

>HLH26_CAEEL/16-66

HGHRSETEKQRRDDTNDLLNEFKKIVQKSESEKLSKEEVLFRIVKLLSGIQ

>Q9W4J8_DROME/255-343

RRATHNEVERRRRDKINSWIFKLKEMLPSLSSSSSFSEASTSPSTSGSTSTNGSSHSKGN

ASSSSGRAPPNDSKSQILIKACEYIKSMQ

>MXL3_CAEEL/48-99

RRAHHNELERRRRDHIKDHFTILKDAIPLLDGEKSSRALILKRAVEFIHVMQ

>MAX_HUMAN/24-75

KRAHHNALERKRRDHIKDSFHSLRDSVPSLQGEKASRAQILDKATEYIQYMR

>MAX_DROME/40-91

KRAHHNALERRRRDHIKESFTNLREAVPTLKGEKASRAQILKKTTECIQTMR

>MYC_DROME/626-678

KRNQHNDMERQRRIGLKNLFEALKKQIPTIRDKERAPKVNILREAAKLCIQLT

>MYC_BOVIN/355-407

KRRTHNVLERQRRNELKRSFFALRDQIPELENNEKAPKVVILKKATAYILSVQ

>MYCL_HUMAN/282-334

KRKNHNFLERKRRNDLRSRFLALRDQVPTLASCSKAPKVVILSKALEYLQALV

>MYCP1_HUMAN/276-328

KKKYHSYLERKRRNDQRSRFLALRDEVPALASCSRVSKVMILVKATEYLHELA

>MNT_HUMAN/221-272

TREVHNKLEKNRRAHLKECFETLKRNIPNVDDKKTSNLSVLRTALRYIQSLK

>E1JJD1_DROME/247-300

TREVHNKLEKERRAQLKECYDLLKKVLPMGDEDRKKTSNLTILDTAHKYVNSLS

>MAD3_RAT/58-110

GRHVHNELEKRRRAQLKRCLEQLRQQMPLGVDHTRYTTLSLLRGARMHIQKLE

>MAD4_HUMAN/54-106

NRSSHNELEKHRRAKLRLYLEQLKQLVPLGPDSTRHTTLSLLKRAKVHIKKLE

>MAD1_HUMAN/57-109

SRSTHNEMEKNRRAHLRLCLEKLKGLVPLGPESSRHTTLSLLTKAKLHIKKLE

>Q9NDP3_CIOIN/57-109

NRTSHNELEKNRRAHLRNCLDGLKAIVPLNQDATRHTTLGLLTQARALIENLK

>MXI1_HUMAN/68-120

NRSTHNELEKNRRAHLRLCLERLKVLIPLGPDCTRHTTLGLLNKAKAHIKKLE

>HLH11_CAEEL/113-164

RRQIANCNERRRMQSINAGFLALRALLPRKEGEKLSKAAILQQTADMVHQLL

>Q9JIZ5_MOUSE/49-100

RREIANSNERRRMQSINAGFQSLKTLIPHTDGEKLSKAAILQQTAEYIFSLE

>Q18056_CAEEL/93-150

RRKVKTEREKIRRKKQDDCYAELKFFILNKQMGSYEQRLKLERITILEIIIDYIKHNS

>ID4_HUMAN/53-105

AEAAADEPALCLQCDMNDCYSRLRRLVPTIPPNKKVSKVEILQHVIDYILDLQ

>ID1_HUMAN/56-106

LLDEQQVNVLLYDMNGCYSRLKELVPTLPQNRKVSKVEILQHVIDYIRDLQ

>ID2_HUMAN/24-76

RSKTPVDDPMSLLYNMNDCYSKLKELVPSIPQNKKVSKMEILQHVIDYILDLQ

>DA_DROME/555-608

RRQANNARERIRIRDINEALKELGRMCMTHLKSDKPQTKLGILNMAVEVIMTLE

>ITF2_CHICK/8-61

RRMANNARERLRVRDINEAFKELGRMVQLHLKSDKPQTKLLILHQAVAVILSLE

>Q9W7E6_ORYLA/51-103

KREMVNAKERLRIRNLNTMFSRLKRMLPLMQPDKKPSKVDTLKAATEYIRLLL

>FIGLA_MOUSE/60-112

RRRVANAKERERIKNLNRGFAKLKALVPFLPQSRKPSKVDILKGATEYIQILG

>Q9VL05_DROME/59-111

KRNTANKKERRRTQSINNAFSYLREKIPNVPTDTKLSKIKTLKLAILYINYLV

>HAND1_CHICK/84-136

KGVGGPKKERRRTESINSAFAELRECIPNVPADTKLSKIKTLRLATSYIAYLM

>SCX_MOUSE/79-131

QRHTANARERDRTNSVNTAFTALRTLIPTEPADRKLSKIETLRLASSYISHLG

>TWIST_CAEEL/21-72

QRACANRRERQRTKELNDAFTLLRKLIPSMPSDKMSKIHTLRIATDYISFLD

>TWST2_RAT/67-118

QRILANVRERQRTQSLNEAFAALRKIIPTLPSDKLSKIQTLKLAARYIDFLY

>PTF1A_RAT/163-215

LRQAANVRERRRMQSINDAFEGLRSHIPTLPYEKRLSKVDTLRLAIGYINFLS

>FER3_DROME/87-139

QRRAANIRERRRMFNLNEAFDKLRRKVPTFAYEKRLSRIETLRLAITYIGFMA

>HLH13_CAEEL/42-94

ERQTASIRERKRMCSINVAFIELRNYIPTFPYEKRLSKIDTLNLAIAYINMLD

>B4HLD1_DROSE/155-206

RERKRIQRSAPTGSINSAFDELRVHVPTFPYEKRLSKIDTLRLAIAYISLLR

>DEI_DROME/95-154

RRKTANARERTRMREINTAFETLRHCVPEAIKGEDAANTNEKLTKITTLRLAMKYITMLT

>ATOH8_MOUSE/232-284

RRLLANARERTRVHTISAAFEALRKQVPCYSYGQKLSKLAILRIACNYILSLA

>B4Q5L5_DROSI/274-326

RRIEANARERTRVHTISAAYETLRQAVPAYASTQKLSKLSVLRVACSYILTLS

>Q9VHG3_DROME/180-233

YRRTACDRERTRMRDMNRAFDLLRSKLPISKPNGKKYSKIESLRIAINYINHLQ

>HLH4_CAEEL/5-57

VAKRNARERTRVHTVNQAFLVLKQHLPSLRQFTKRVSKLRILNAAITYIDTLL

>HLH6_CAEEL/175-226

VWKRNERERCRVRNVNDGYERLRKHLPVHFDEKRISKVDTLRLAIRYIKHLD

>ASCL3_MOUSE/93-145

FIRKRNERERQRVKCVNEGYARLRRHLPEDYLEKRLSKVETLRAAIKYISYLQ

>HLH14_CAEEL/5-57

NQVARNERERKRVHQVNHGFDVLRNRLQPKNHTKKWSKADTLREAVKYIQQLQ

>HLH3_CAEEL/28-80

KQKRNERERKRVDQVNQGFVLLQERVPKAAGNKAKLSKVETLREAARYIQELQ

>A0A212ENF6_DANPL/75-137

IARRNARERNRVKQVNDGFNALRRHLPASVVAALSGGARRGSGKKLSKVDTLRMVVEYIR

YLQ

>AST5_DROME/26-91

VIRRNARERNRVKQVNNGFSQLRQHIPAAVIADLSNGRRGIGPGANKKLSKVSTLKMAVE

YIRRLQ

>AST8_DROME/161-224

VARRNARERNRVKQVNNGFALLREKIPEEVSEAFEAQGAGRGASKKLSKVETLRMAVEYI

RSLE

>AST3_DROME/85-146

VARRNARERNRVKQVNNGFVNLRQHLPQTVVNSLSNGGRGSSKKLSKVDTLRIAVEYIRG

LQ

>F7CZE1_XENTR/46-98

SERRNERERNRVKLVNLGFAKLRQHVPQAQGPNKKMSKVETLRSAVEYIRALQ

>AST4_DROME/101-163

VQRRNARERNRVKQVNNSFARLRQHIPQSIITDLTKGGGRGPHKKISKVDTLRIAVEYIR

RLQ

>Q18277_CAEEL/14-66

RRSRANERERQRVSEMNGMFDVLLNLLPPSHFKTRLSRVQILREATSYIIRLH

>HND1_CAEEL/24-78

RKEKSREKEHRRAQCINSAFEILQQHIPYLKSEERKSLPKIKTLRLAMQYIDHLK

>F6YKF5_CIOIN/394-445

RRRAATLRERRRLKRVNQAYDALKRCACANPNQRLPKVEILRNAITYIYNLQ

>H2R2K4_PANTR/107-158

RRRAATLREKRRLKKVNEAFEALKRSTLLNPNQRLPKVEILRSAIQYIERLQ

>TAP_DROME/155-207

RRMKANDRERNRMHNLNDALEKLRVTLPSLPEETKLTKIEILRFAHNYIFALE

>NDF1_CAEEL/20-72

RRVKANGRERARMHGLNNALDMLREYIPITTQHQKLSKIETLRLARNYIDALQ

>LIN32_CAEEL/73-125

RRSAANERERRRMNTLNVAYDELREVLPEIDSGKKLSKFETLQMAQKYIECLS

>G3TPA8_LOXAF/124-176

RRLAANARERRRMHGLNHAFDQLRNVIPSFNNDKKLSKYETLQMAQIYINALS

>ATOH7_CHICK/40-92

RRLAANARERRRMQGLNTAFDRLRKVVPQWGQDKKLSKYETLQMALSYIMALT

>AMOS_DROME/139-191

RRLAANARERRRMNSLNDAFDKLRDVVPSLGHDRRLSKYETLQMAQAYIGDLV

>ATO_DROME/256-308

RRLAANARERRRMQNLNQAFDRLRQYLPCLGNDRQLSKHETLQMAQTYISALG

>BHA15_RAT/73-125

RRLESNERERQRMHKLNNAFQALREVIPHVRADKKLSKIETLTLAKNYIKSLT

>O45320_CAEEL/41-95

LRNSINSRERRRMHELNDEFETLRECLPYPNEANSRRMSKANTLLLASNWIKQLA

>OLIG3_MOUSE/85-139

LRLKINGRERKRMHDLNLAMDGLREVMPYAHGPSVRKLSKIATLLLARNYILMLT

>OLIG1_MOUSE/95-154

LRRKINSRERKRMQDLNLAMDALREVILPYSAAHCQGAPGRKLSKIATLLLARNYILLLG

>B4NNG8_DROWI/32-84

QRNAANARERMRMRVLSSAYGRLKTKLPNIPPDTKLSKLDTLRLATLYIKQLI

>MUSC_HUMAN/108-160

QRNAANARERARMRVLSKAFSRLKTSLPWVPPDTKLSKLDTLRLASSYIAHLR

>TCF23_MOUSE/76-128

ASPENAARERTRVKTLRQAFLALQAALPAVPPDTKLSKLDVLVLATSYIAHLT

>HLH10_CAEEL/122-173

RRYEANARERNRVQQLSKMFDQLRVCLPIEDDAKISKLATLKVASSYIGYLG

>Q28DS7_XENTR/96-150

VRNSASEREKMRMRNLSSALQNLRRYLPPAVAPIGKTLTKIETLRLTIRYISHLS

>MSGN1_CHICK/96-150

RRRKASEREKLRMRTLADALHTLRNYLPPAYSQRGQPLTKIQTLKCTIKYISELT

>R4GGT1_CHICK/68-122

PRQSASEREKLRMRRLAQAMHRLRHYLPPALAPAGQSLTKIETLRLATRYIAHLS

>F1Q9Z5_DANRE/67-121

QRQNASEKEKLRMRDLTKALHHLRSFLPASVAPVGQTLTKIETLRLTIQYISFLS

>Q9IAJ4_DANRE/88-142

KRQTASEREKLRMRDLTKALHHLRTFLPASVAPVGKTLTKIETLRLAIKYISCLS

>MESP1_MOUSE/77-131

QRQSASEREKLRMRTLARALHELRRFLPPSVAPTGQNLTKIETLRLAIRYIGHLS
