# Supplementary material for: Systematic identification and expression analysis of bHLH gene family reveal their relevance to abiotic stress response and anthocyanin biosynthesis in sweetpotato
Source: BMC Plant Biol. 2024 Mar 1;24:156. doi: 10.1186/s12870-024-04788-0 (PMC10905920; doi:10.1186/s12870-024-04788-0)
Supplement: Supplementary file 10 — Supplementary Material 10 [file 12870_2024_4788_MOESM10_ESM.docx]

## **Additional file 15**. Specific primer sequences used for the qRT-PCR analysis.

| Primer code | Primer sequences (5' →3') | | Application | |
| --- | --- | --- | --- | --- |
| IbARF-Q-F | | CTTTGCCAAGAAGGAGATGC | Internal standard gene | |
| IbARF-Q-R | | CTTGTCCTGACCACCAACA | for qRT-PCR analysis | |
| IbbHLH5-Q-F | | TTCGTCAAGAAGTCCCAGGTATC | qRT-PCR analysis | |
| IbbHLH5-Q-R | | TGCTCATCGGAGTTCCCATT | for *IbbHLH* genes | |
| IbbHLH25-Q-F | | CAATCGTAATAATAGGCGTGAATG |  | |
| IbbHLH25-Q-R | | AGCGTTGTTGAACTCTATTGGTATC |  | |
| IbbHLH69L-Q-F | | GCCGAGAGGACATCAGATAACT |  | |
| IbbHLH69L-Q-R | | TGTTTCGCAGTCCAGATGTAGAT | |  |
| IbbHLH106-Q-F | | TGACGAGTTTAGGCGGCAG | |  |
| IbbHLH106-Q-R | | CCTTTGCCTCTTACTTTTGATACG | |  |
| IbbHLH123-Q-F | | TTCCAAGGTTGTCTCCGCTA | |  |
| IbbHLH123-Q-R | | GCGGCTCGGGCTTATTACTA | |  |
| IbbHLH43L-Q-F | | AACGGCGGTGAAGGAAGA | |  |
| IbbHLH43L-Q-R | | GATGAGATAAACGGGCAGGAG | |  |
| IbbHLH148-Q-F | | AAGATGGAGGCTAAAGACGAACA | |  |
| IbbHLH148-Q-R | | GAGGATTTGGTGGGTGGTGT | |  |
| IbbHLH154L-Q-F | | CACCCCTACCACCACTCCAA | |  |
| IbbHLH154L-Q-R | | TCACTTCCACATCCGCCAG | |  |
| IbbHLH180L-Q-F | | AGACCTCAAACCAACCCGAT | |  |
| IbbHLH180L-Q-R | | TCAGCGGTAATGTTGCTTGTG | |  |
| IbbHLH206-Q-F | | GAAAAATGGTTGTTCAGTCTCTCA | |  |
| IbbHLH206-Q-R | | GAAATCGGAACTCGTCTCGG | |  |
| IbbHLH212L-Q-F | | TGAACACTTTAGAGGTTTATGAGAGG | |  |
| IbbHLH212L-Q-R | | AAGATTTTGAGAGAAGGGTTGCT | |  |
| IbbHLH214-Q-F | | CGAAGAAGAAATCAGCACAGGTC | |  |
| IbbHLH214-Q-R | | GCTGGCTCATCGGAACTACC | |  |
| IbbHLH20-Q-F | | GTGGGCGATTACGCAGTG | |  |
| IbbHLH20-Q-R | | AACCACGCTTTGCTCTAACC | |  |
| IbbHLH65-Q-F | | CTAGATGTCACCCATGCCAGTGT | |  |
| IbbHLH65-Q-R | | TTGGATGTCAATGCTAACCGAAG | |  |
| IbbHLH74-Q-F | | GCCTGATGAAGTTACAAACCCTA | |  |
| IbbHLH74-Q-R | | AGCTTCTAGTGTGCTAACTGTTGAC | |  |
| IbbHLH124L-Q-F | | CGCCTCCAGATGTATAACTGTGAG | |  |
| IbbHLH124L-Q-R | | GTCCAGTTTTCTTTGATGATCTCG | |  |
| IbbHLH131L-Q-F | | TCCCTTCCCCCTCAATGTCT | |  |
| IbbHLH131L-Q-R | | ATCCTCGGGGTGCAACATAGT | |  |
| IbbHLH135L-Q-F | | GTGAAAATAATCGGCTGGGAC | |  |
| IbbHLH135L-Q-R | | GCTGCCCATCTTGACCGT | |  |
| IbbHLH151L-Q-F | | GCTGTTTCAATGAGTTCGCAA | |  |
| IbbHLH151L-Q-R | | GGCACCTTCCTCCATATCCT | |  |
| IbbHLH153L-Q-F | | GAGATGTGGAAAGTGAATGGCA | |  |
| IbbHLH153L-Q-R | | TCATTACCACAACTAGATCCTGCA | |  |
| IbbHLH175-Q-F | | CTCTCTTAGCCTCAAGGTGGAA | |  |
| IbbHLH175-Q-R | | CTTGAATCCGAGCCATAATCC | |  |
| IbbHLH176L-Q-F | | CTCCTCTCCTAGCATGTCGGT | |  |
| IbbHLH176L-Q-R | | GGCTTAGAAACGGAAGTGGC | |  |
| IbbHLH196L-Q-F | | TAAGATGGAGGGGACAAAAGC | |  |
| IbbHLH196L-Q-R | | CACTTACATACAAGGGTGTGACAGA | |  |
| IbbHLH2-Q-F | | TTGGAGGTCATGCAGATGCTTA |  | |
| IbbHLH2-Q-R | | GCCTTTTTCACTTCCATGATTGT |  | |
|  |  | | |  |
|  |  | | |  |
