# Supplementary material for: Systematic identification and expression analysis of bHLH gene family reveal their relevance to abiotic stress response and anthocyanin biosynthesis in sweetpotato
Source: BMC Plant Biol. 2024 Mar 1;24:156. doi: 10.1186/s12870-024-04788-0 (PMC10905920; doi:10.1186/s12870-024-04788-0)

**Additional file 1**. Chromosomal map showing the uneven distribution of 227 *IbbHLH* genes on 15 sweetpotato chromosomes. The chromosome numbers are indicated to the left of each chromosome as LG1-LG15. The scales were indicated the genome size of sweetpotato genome (Mb), , and tandem duplicated genes are indicated as green arcs.


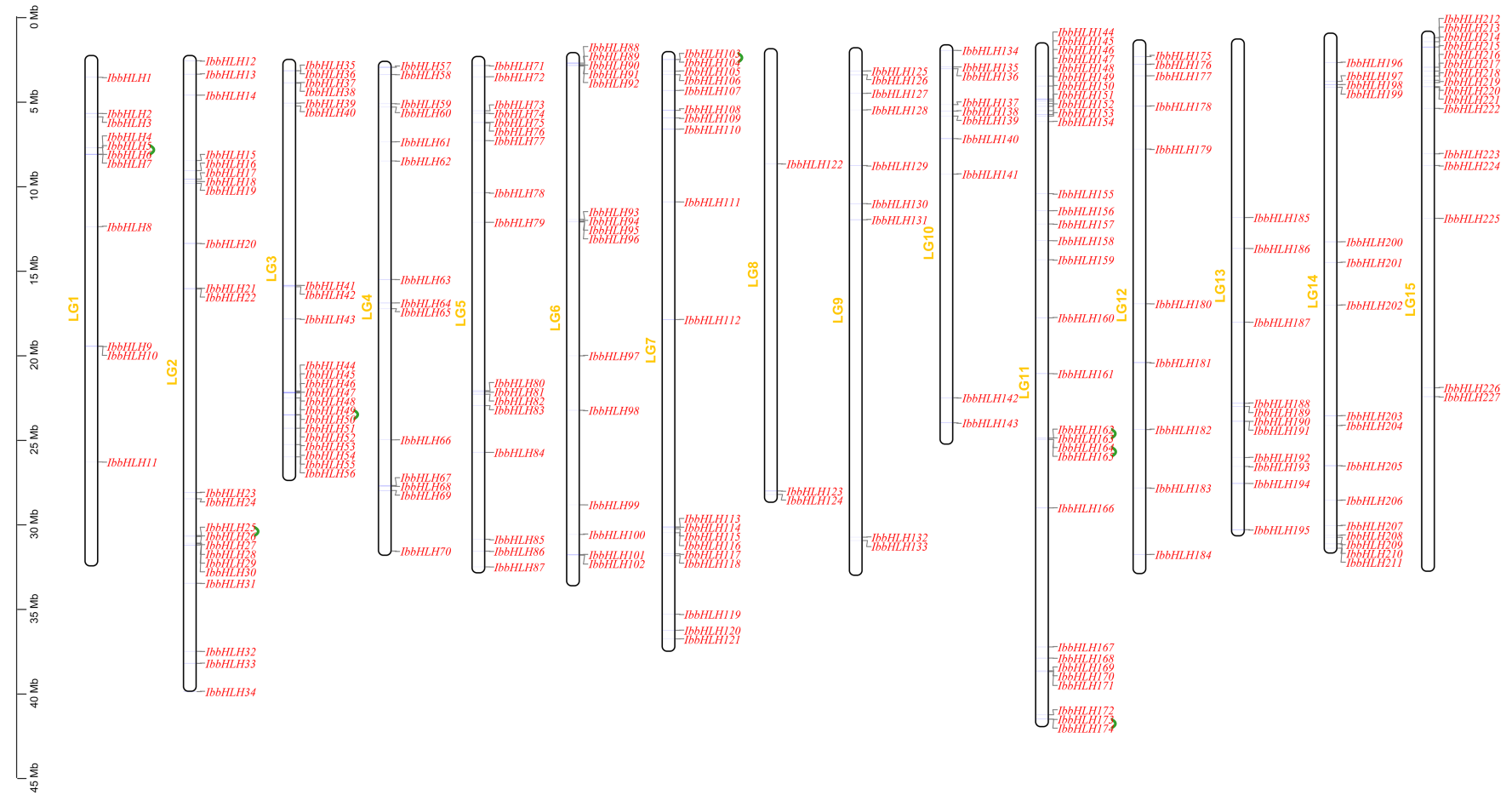


**Additional file 5**. Phylogenetic relationships and conserved motif compositions in sweetpotato IbbHLHs. (A) The phylogenetic tree of 227 IbbHLHs was constructed by MEGA X based on the consistent parameters used in Fig. 1. (B) Conserved motif compositions detected by MEME analysis in sweetpotato IbbHLHs. Boxes of different colors present different motifs. The length of IbbHLHs can be estimated by the scale at the bottom.


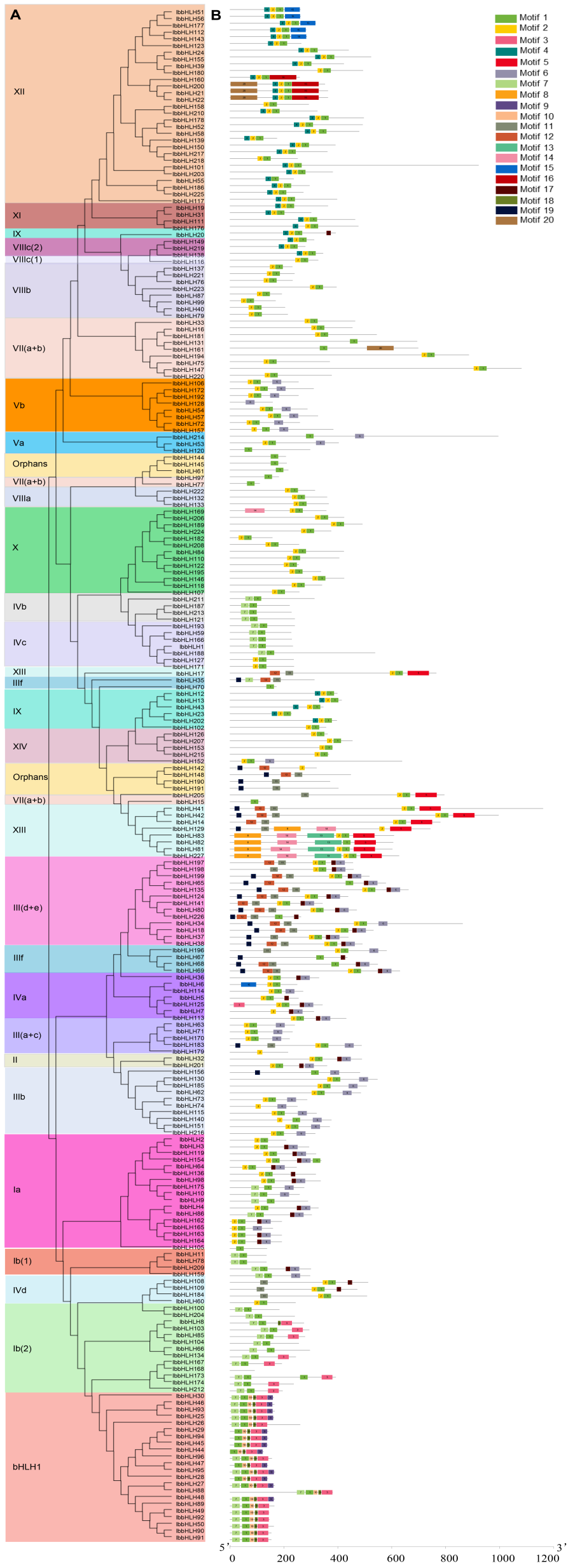


**Additional file 10**. Phylogenetic clustering and predicted stress- and hormone-related cis-elements in the promoters of *IbbHLH* genes. **A**. The phylogenetic tree of 227 IbbHLHs was constructed by MEGA X based on the consistent parameters used in Fig. 1. Different subgroups and their IbbHLH members are displayed in different colors. **B**. Predicted cis-elements in the *IbbHLH* promoters. 2000 bp promoter sequences of each *IbbHLH* gene were scanned by PlantCARE database. Different colors represent different cis-elements.

**
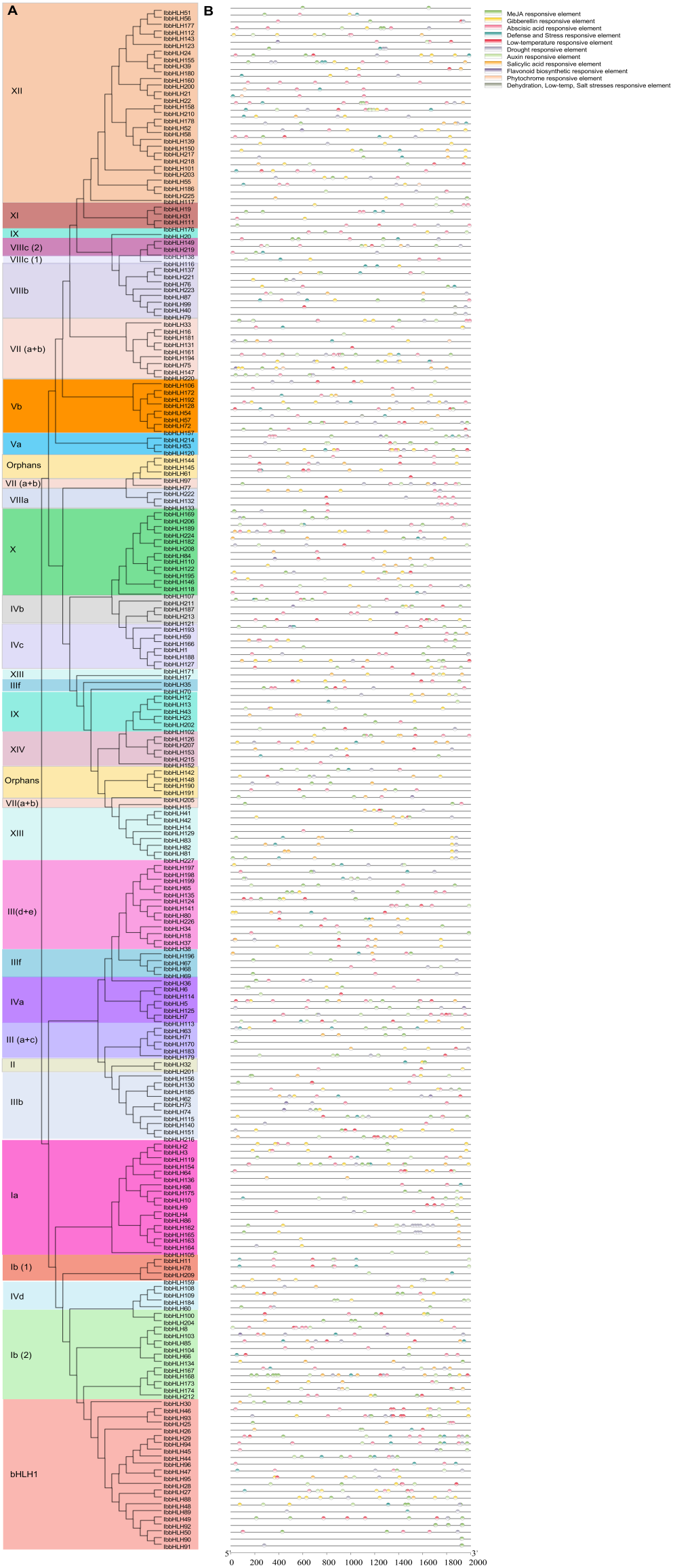
**

**Additional file 11**. Interaction networks of IbbHLH proteins in sweetpotato according to the orthologues in Arabidopsis. The amino acid sequences of IbbHLHs were employed to search the STRING database, network node represents proteins, and edge represents protein–protein associations. The colored lines between the nodes indicate the different kinds of interactions. The numbers (IbbHLH gene number) in brackets represent the corresponding orthologues in sweetpotato.


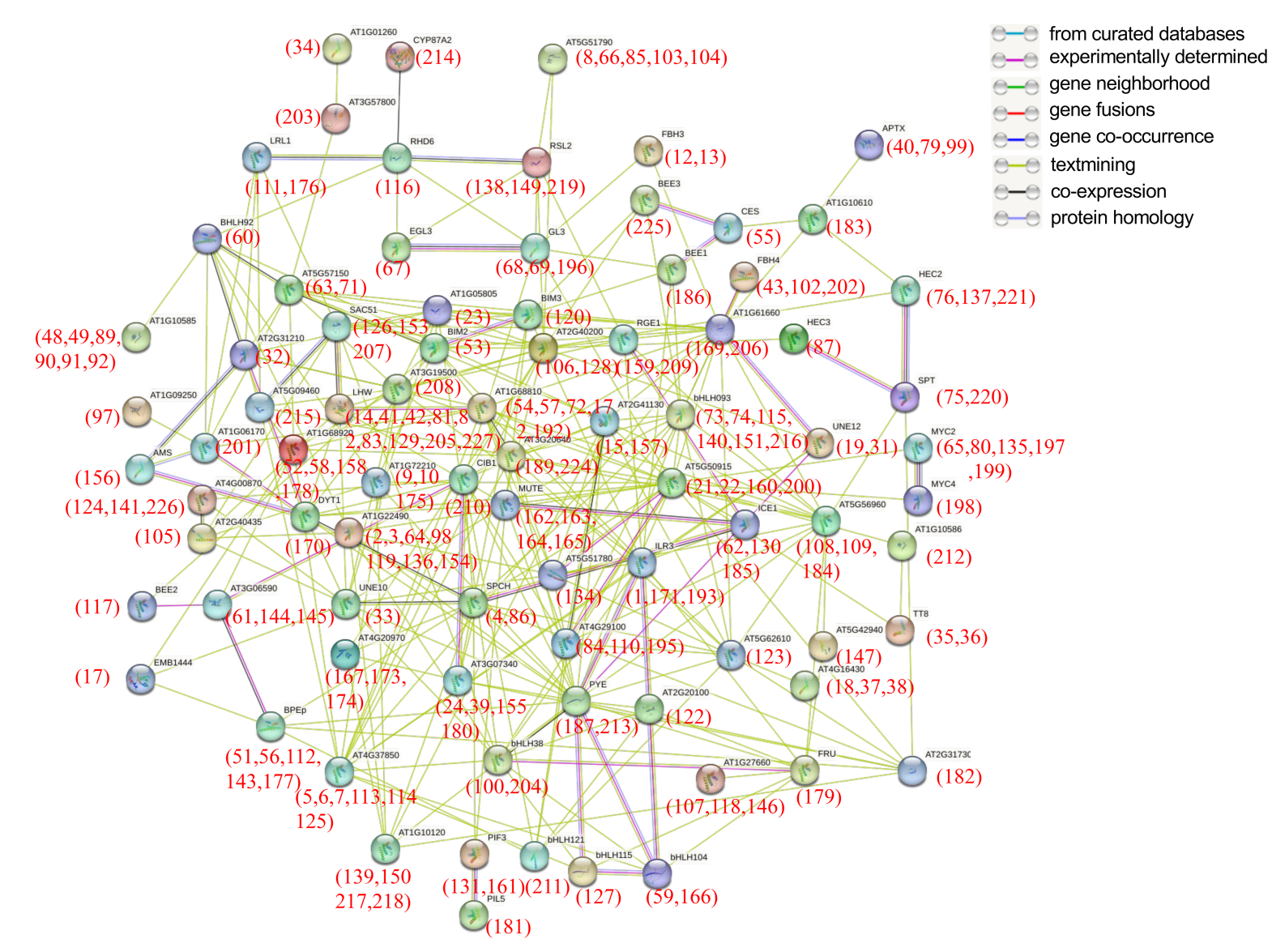

Supplement: Supplementary file 12 — Supplementary Material 12 [file 12870_2024_4788_MOESM12_ESM.docx]
